# Efficient aerobic oxidation of benzyl alcohols using a magnetically recoverable Ni/nitrogen-containing carbon nanocomposite

**DOI:** 10.1039/d6ra02052f

**Published:** 2026-05-21

**Authors:** Dinesh Babu Arumugam, K. Santhakumar

**Affiliations:** a Department of Chemistry, School of Advanced Sciences, Vellore Institute of Technology Vellore – 632014 India ksanthakumar@vit.ac.in

## Abstract

A magnetically recoverable nickel nanoparticle catalyst supported on polyaniline-derived carbon (Ni/PDC) was developed for the aerobic oxidation of benzyl alcohols. Polyaniline-derived carbon provides a nitrogen-containing carbon framework that facilitates the stabilization and uniform dispersion of nickel nanoparticles. The catalyst was synthesized *via* a hydrothermal method, and the reactions were carried out in isopropanol using molecular oxygen as the oxidant. Structural and surface characterization confirmed the presence of metallic Ni^0^ together with oxidized nickel species within the nitrogen-containing carbon framework, which likely facilitates oxygen activation and promotes the oxidation process. The catalyst exhibited good activity toward a variety of substituted benzyl alcohols and showed tolerance to different functional groups. Moreover, the catalyst could be readily separated from the reaction mixture using an external magnet and reused for seven consecutive cycles without significant loss of catalytic activity. Gram-scale reactions further demonstrated the practical applicability of this catalytic system. Overall, the Ni/PDC catalyst provides a cost-effective and reusable nickel-based system for the synthesis of value-added aromatic aldehydes such as vanillin, anisaldehyde, and indole-3-carboxaldehyde under mild aerobic conditions, and its sustainability profile was further assessed using the CHEM21 green metrics toolkit.

## Abstract 2

A magnetically recoverable nickel nanoparticle catalyst supported on polyaniline-derived carbon (Ni/PDC) was developed for the aerobic oxidation of benzyl alcohols.

## Graphical abstract

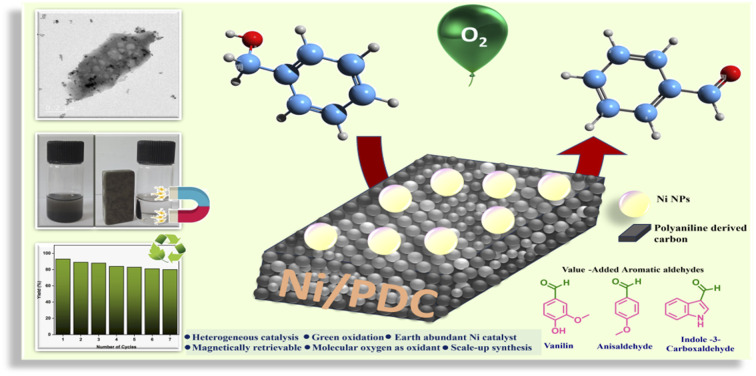

## 1. Introduction

Converting inexpensive and readily available feedstock chemicals into value-added products remains an important goal in chemical synthesis, particularly for industrial and research applications. In particular, the oxidation of benzyl alcohols to benzaldehydes is an important reaction due to the broad utility of benzaldehydes as synthetic intermediates. Benzaldehyde derivatives are key intermediates widely employed in the manufacture of fragrances, cosmetics, food flavorings, dyes, agrochemicals, plastic additives, and pharmaceutical compounds.^1,2^ Consequently, the development of efficient, selective, and environmentally benign methods for this oxidation reaction remains an active area of research.

Conventionally, benzyl alcohol to benzaldehyde has been accomplished utilizing stoichiometric amounts of chromium(vi)-based oxidants^3,4^ such as pyridinium chlorochromate (PCC), pyridinium dichromate (PDC), dichromates, and CrO_3_. Manganese-based oxidants such as MnO_2_ ^5^ and permanganates^6^ have also been widely used. In addition, hypervalent iodine reagents^7^ and DMSO-based systems, including Dess–Martin periodinane^8^ and Swern reagents, are commonly employed for this transformation.^9^ Although effective, many of these methods suffer from poor atom economy and generate hazardous waste, raising significant environmental and health concerns.^10^ The increasing emphasis on sustainable chemistry has therefore driven the search for greener oxidation strategies that minimize hazardous byproducts and improve overall process of sustainability.

Among the various alternatives explored, utilizing molecular oxygen as a terminal oxidant has emerged as an attractive and environmentally friendly approach. Oxygen is abundant, inexpensive, and when efficiently utilized, produces water (H_2_O) as the primary byproduct, thereby significantly reducing waste generation.^1,11–13^ However, the effective activation of molecular oxygen under mild conditions typically requires the presence of suitable catalysts.

**Scheme 1:**
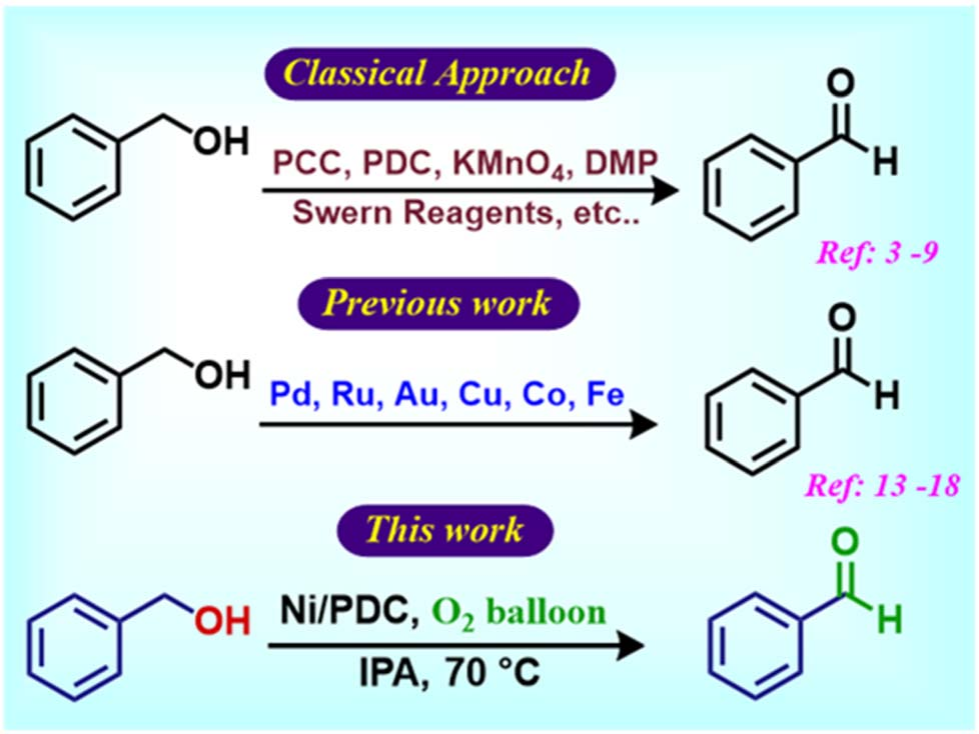
Benzyl alcohol oxidation reactions.

Both homogeneous and heterogeneous catalytic systems based on transition metals such as Ru,^14^ Pd,^15^ Au,^16^ Cu,^17^ Co^18^ and Fe^19^ have been widely investigated for aerobic alcohol oxidation.^20–23^ While homogeneous catalysts often exhibit high activity, however, their practical applicability is limited by difficulties in catalyst recovery, recycling and potential metal contamination of the products. In contrast, heterogeneous catalysts provide several benefits including easy catalyst recovery, improved reusability, greater compatibility with large-scale processes, making them more suitable for sustainable industrial applications (Scheme 1).^24,25^

In the past few years, significant efforts have been made toward the development of non-noble metal heterogeneous catalysts, particularly those base-metals such as Fe, Co, Mn, and Ni.^26^ Several catalytic methods have been reported for the selective oxidation of benzyl alcohol to benzaldehyde. For instance, Fereshteh and co-workers reported Fe nanoparticle-based nanocomposites as efficient catalysts for this transformation.^27^ Li *et al.* developed a palladium-doped cobalt magnetic catalyst exhibiting high selectivity toward benzaldehyde.^28^ Aimin Zhang and co-authors described Mn composites as effective catalysts for benzyl alcohol oxidation^29^ while Yanhui Yang and colleagues reported carbon-supported manganese catalysts showing promising catalytic performance in the same reaction.^30^

Nickel is considered an attractive transition metal in catalysis because of its natural abundance, relatively low cost, and diverse reactivity. Since its early application in hydrogenation chemistry in the late nineteenth century, nickel has been employed in numerous organic transformations, including hydrogenolysis, cycloaddition, reduction, oxidation, amination, and cross-coupling reactions.^31,32^ In recent years, nickel-based catalysts have also been explored for aerobic oxidation processes. Their adaptable electronic structure and strong interaction with oxygen-containing substrates allow efficient activation of both alcohols and molecular oxygen. In particular, supported and bimetallic nickel systems, such as Ni–Co oxides, Ni–Fe materials, and other Ni-based composites, have demonstrated encouraging activity in the selective oxidation of benzyl alcohol to benzaldehyde.^33–36^

Catalytic performance is often influenced by the nature of the support, as it governs metal dispersion, surface characteristics, and the activation of reactants such as oxygen. Careful selection and design of support materials can therefore significantly affect overall activity. Among the various options, nitrogen-doped carbon materials have attracted attention because they can alter the electronic structure of supported metal species and promote stronger metal–support interactions. Polyaniline (PANI), a nitrogen-rich conducting polymer, is frequently used as a precursor for preparing such N-doped carbon frameworks. Its relatively low cost, straightforward synthesis, and high nitrogen content make PANI-derived carbons practical alternatives to traditional supports like graphene, carbon nanotubes, and activated carbon.^37^ Furthermore, the hydrothermal synthesis method is recognized as an efficient and versatile approach for preparing supported metal catalysts with controlled particle size, morphology, and crystallinity. This synthesis method is cost-effective, rapid, simple, environmentally benign and well suited for the uniform incorporation of metal species into polymer-derived carbon matrices.^38^

The present study reports the synthesis of polyaniline-derived carbon supported nickel (Ni/PDC) composite catalysts through a combined hydrothermal and subsequent pyrolysis approach. These ensuing materials have ferromagnetic characteristics and effectively use molecular oxygen as an environmentally safe oxidant to catalyse the selective oxidation of several benzyl alcohol derivatives. An external magnetic field can be used to separate the catalysts from the reaction mixture due to their magnetic characteristics and utilized again for seven cycles in a row without experiencing any appreciable loss of activity. To clarify the structural, morphological, and surface properties of the catalysts, a thorough evaluation was carried out using XRD, FE-SEM, EDS, TEM, N_2_ surface adsorption–desorption BET study, VSM, and XPS. Furthermore, correlation between catalyst structure and catalytic performance was systematically examined, highlighting the crucial role of the PANI-derived carbon support in promoting nickel-based aerobic oxidation.

## 2. Experimental section

### 2.1 Synthesis of Ni/PDC nanocomposite

**Fig. 1:**
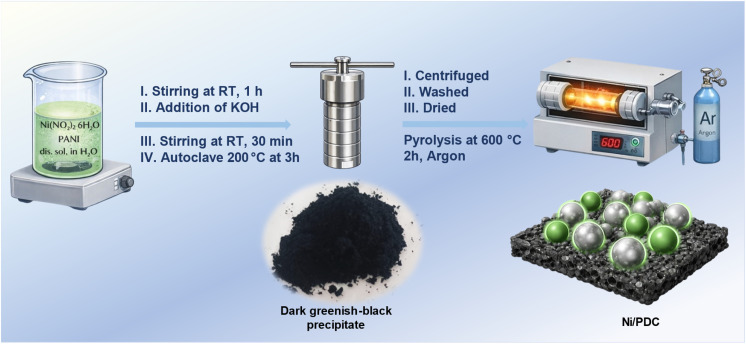
Ni/PDC catalyst schematic representation.

Ni(NO_3_)_2_·6H_2_O (1.2 g) and polyaniline (1.0 g) were dissolved in 50 mL of deionized water and agitated for one hour at room temperature. After adding KOH dropwise and stirring for 30 minutes, the pH was brought to 10. After that, the mixture was hydrothermally treated in an autoclave lined with Teflon for three hours at 200 °C. After the reaction concluded, it was allowed to cool to room temperature. Centrifugation was used to recover the solid product, which was then repeatedly cleaned with ethanol and water to obtain removal of any remaining impurities and dried for 12 hours at 60 °C. To obtain the Ni/PDC nanocomposite catalyst, the dried sample was then pyrolyzed at 600 °C for two hours under argon at a heating rate of 5 °C min^−1^ (see Fig. 1).

### 2.2 Ni/PDC catalyst for oxidation of benzyl alcohols

Benzyl alcohol (0.5 mmol) and Ni/PDC catalyst (15 mg) were added to a 50 mL round-bottom flask fitted with a magnetic stir bar and 3 mL of isopropanol was then added. To keep the temperature constant, the reaction was conducted in an oxygen environment and stirred in an oil bath at 70 °C for six hours. After the reaction was complete, the catalyst was removed using an external magnet. The crude product was then refined using column chromatography utilizing silica gel (60–120 mesh) and hexane/ethyl acetate as the eluent to get the pure corresponding aldehydes Fig. 10 (2a–2v).

### 2.3 Characterization

Powder X-ray diffraction (XRD) patterns were recorded on a Bruker D8 Advance diffractometer (Germany) equipped with a 2.2 kW Cu anode, a ceramic X-ray tube, and a LynxEye silicon strip detector. The structural features and morphology of the catalysts were examined using a FEI QUANTA 250 FEG field-emission scanning electron microscope (FE-SEM). High-resolution transmission electron microscopy (HR-TEM) images were obtained on a FEI Tecnai G2-20 Twin instrument operated at an accelerating voltage of 200 kV. Surface chemical composition and oxidation states were analyzed by X-ray photoelectron spectroscopy (XPS). The specific surface area and pore size distribution were determined using a Quantachrome BET surface area analyzer (USA). Metal leaching was quantified by inductively coupled plasma mass spectrometry (ICP-MS). Raman spectra were recorded on an Anton Paar Strabe 20 spectrometer (Graz, Austria). Magnetic measurements were carried out using a Lake Shore 7410 vibrating sample magnetometer (VSM).

## 3 Results and discussion

### 3.1 Powder X-ray diffraction (XRD)

**Fig. 2:**
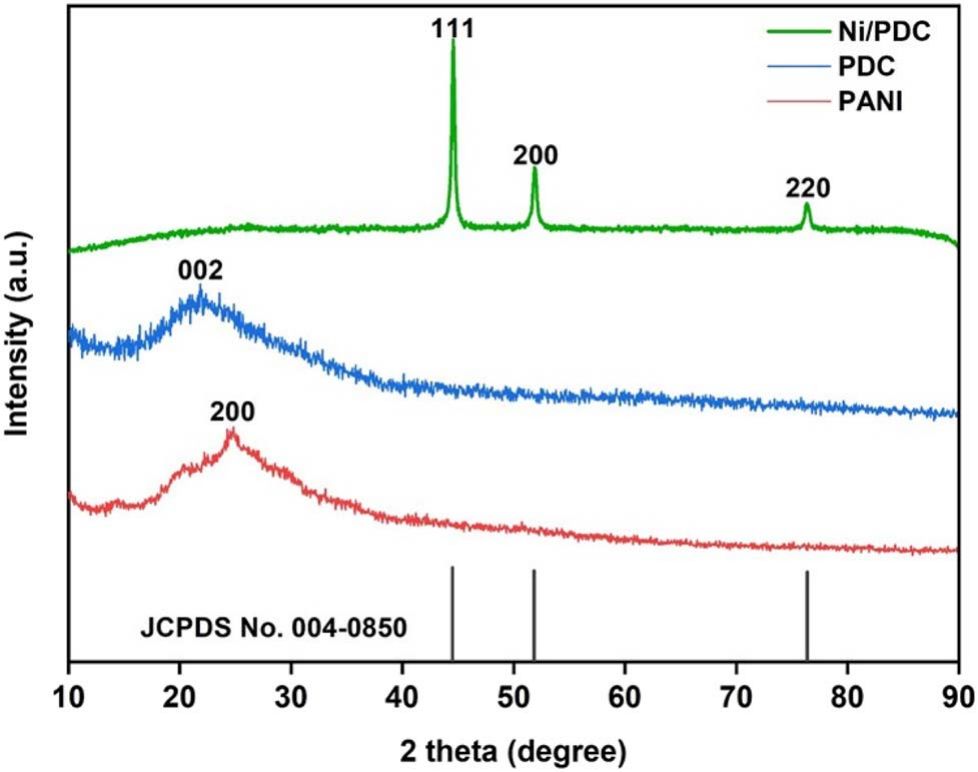
XRD spectra of synthesized Ni/PDC catalyst, poly aniline derived carbon (PDC) and PANI.

Powder X-ray diffraction (XRD) analysis was carried out to investigate the crystalline structure of polyaniline (PANI), polyaniline-derived carbon (PDC), and Ni/PDC nanocomposites (Fig. 2).

The XRD pattern of PANI exhibits characteristic diffraction peaks at 2*θ* values of 14.5°, 20.3°, and 24.7°, corresponding to the (001), (020), and (200) planes, indicating its amorphas nature. Upon carbonization, these characteristic PANI peaks completely disappear, confirming the transformation of PANI into carbonaceous material. The PDC sample shows a broad diffraction peak centered at around 2*θ* ≈ 25.8°, which is attributed to amorphous carbon structure. In contrast, the Ni/PDC catalyst displays distinct diffraction peaks at 2*θ* values of 44.5°, 51.8°, and 76.5°, which can be indexed to the (111), (200), and (220) crystallographic planes of face-centered cubic metallic nickel (Ni^0^). These crystal diffraction peaks are in good agreement with the standard JCPDS card no. 04-0850, confirming the formation of crystalline metallic nickel nanoparticles supported on PDC. The average crystallite size, calculated to be approximately 18 nm from XRD peak using the Scherrer equation, indicates the formation of nanoscale crystalline structure and shows close agreement with the particle size (21 nm) observed from transmission electron microscopy, suggesting the uniform formation of particles with high crystallinity.

### 3.2 Raman analysis

**Fig. 3:**
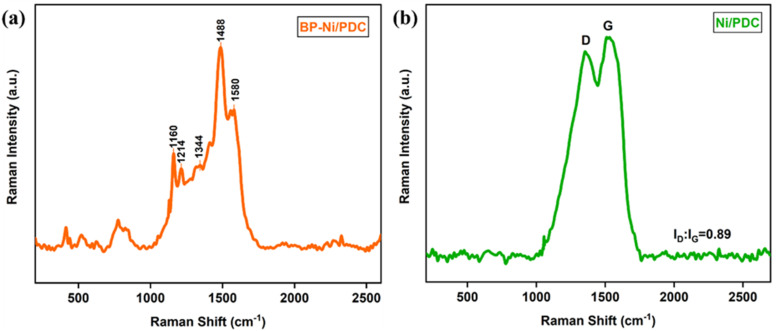
(a and b) Raman spectra of BP-Ni/PDC and Ni/PDC.

Raman spectroscopy was used to examine the structural changes before and after pyrolysis (Fig. 3). The spectrum of the PANI-Ni composite before pyrolysis (Fig. 3a) exhibits multiple bands at ∼1160, 1214, 1344, 1488, and 1580 cm^−1^, corresponding to characteristic vibrational modes of the polyaniline backbone.^39^ After pyrolysis (Fig. 3b), only two prominent peaks appear at ∼1355 and 1580 cm^−1^, assigned to the D and G bands of PANI-derived carbon, respectively. The disappearance of PANI-related peaks and the appearance of D and G bands confirm successful carbonization. The *I*_D_/*I*_G_ ratio of 0.85 suggests a defect-rich carbon structure, which is beneficial for catalytic activity. This heavily defective state is consistent with the broad, amorphous carbon peak previously observed in the XRD pattern, confirming that the high-temperature pyrolysis and simultaneous nitrogen doping significantly disrupted the perfect graphitic lattice.^40,41^

### 3.3 Analysis of morphology

**Fig. 4:**
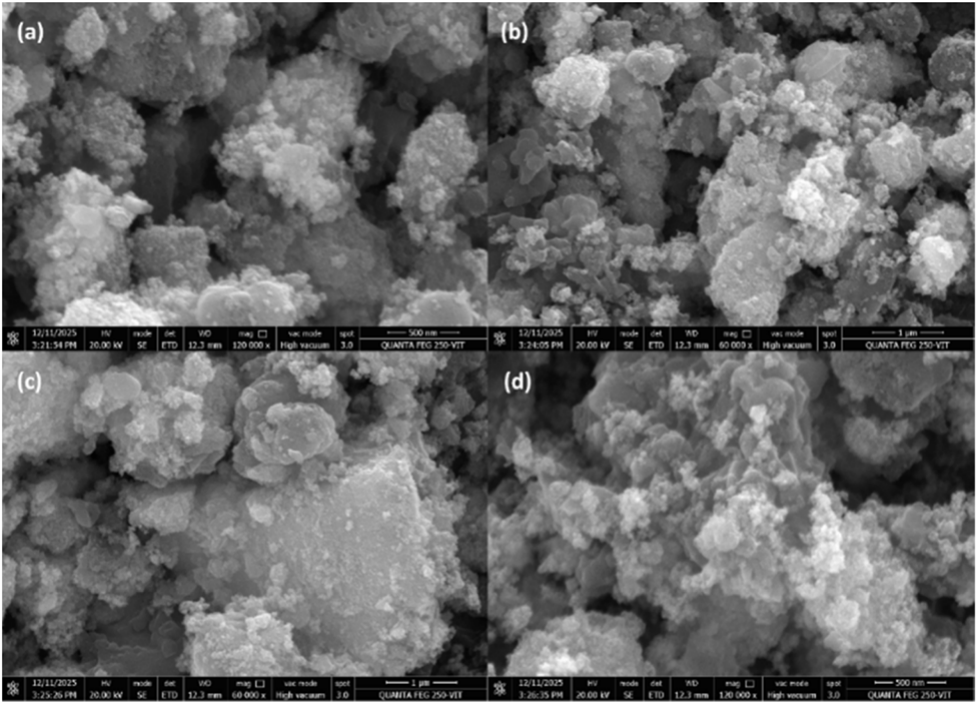
(a–d) FE-SEM images of Ni/PDC.

Field-emission scanning electron microscopy (FE-SEM) images show that the Ni/PDC material consists of aggregated quasi-spherical particles, giving rise to a porous and irregular network-like structure. The observed surface roughness is consistent with the dispersion of nickel nanoparticles across the carbon framework (see Fig. 4a–d).

**Fig. 5:**
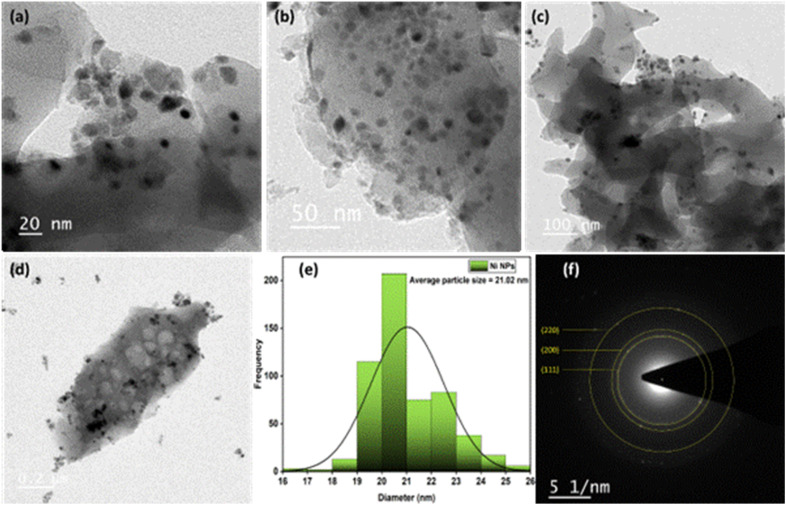
(a–d) TEM images of Ni/PDC. (e) Particle size distribution Curve. (f) SEAD pattern of Ni/PDC.

The structure and dispersion of Ni nanoparticles within the carbon matrix were examined using transmission electron microscopy (TEM). The TEM images (Fig. 5a–d) reveal that nearly spherical Ni nanoparticles are uniformly distributed over the carbon sheets. The particles are well dispersed with slight aggregation and are clearly embedded within thin carbon sheets. The particle size distribution histogram (Fig. 5e) reveals an average nickel nanoparticle size of 21.02 nm. This value is marginally higher than the crystallite size of 18 nm estimated from XRD using the Scherrer equation, indicating a close correlation between particle and crystallite dimensions, reflecting high crystallinity and uniform size distribution. This also highlights the effective control over nucleation and growth processes during catalyst synthesis. The development of face-centered cubic (fcc) crystalline Ni is confirmed by the clear concentric diffraction rings indexed to the (111), (200), and (220) planes in the selected area electron diffraction (SAED) pattern (Fig. 5f).

The concentric ring pattern confirms the highly crystalline nature of the nanoparticles and their random crystallographic orientation within the support matrix. These results confirm the successful formation of well-dispersed crystalline Ni nanoparticles over the polyaniline-derived carbon framework. Successful formation of well-dispersed crystalline Ni nanoparticles over the polyaniline-derived carbon framework. These SAED rings perfectly mirror the distinct fcc metallic nickel peaks identified in the macroscopic XRD analysis, cross-validating the highly crystalline nature of the nanoparticles at both the bulk and single-particle scales.

**Fig. 6:**
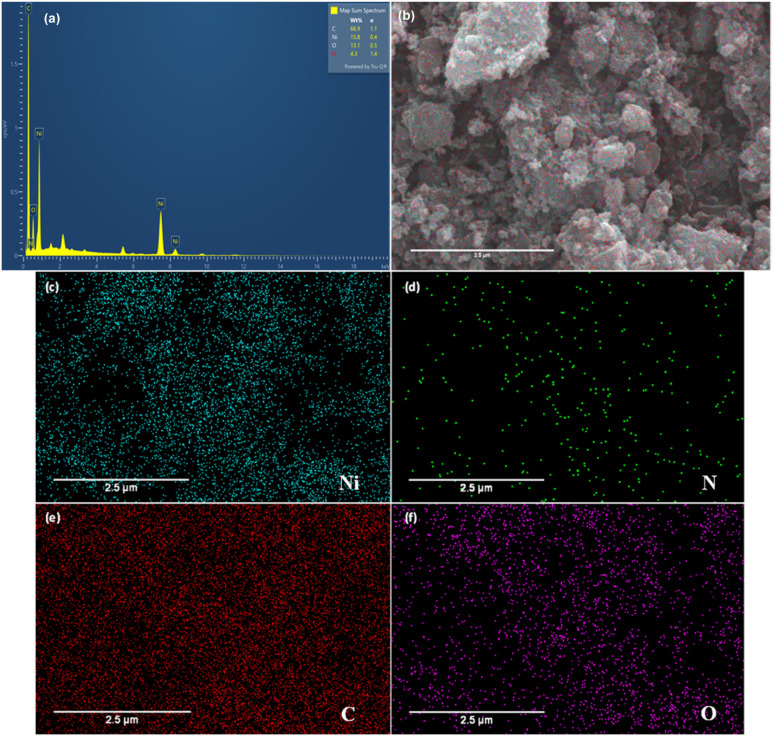
(a) EDX spectrum and (b–f) corresponding elemental mapping images of the Ni/PDC catalyst.

Energy-dispersive X-ray (EDX) analysis confirms the presence of C, Ni, O, and N in the composite. In addition, elemental mapping reveals that these elements are distributed throughout the material without noticeable phase separation, supporting the successful incorporation of nickel within the nitrogen-doped carbon matrix (see Fig. 6a–f).

### 3.4 Analysis of surface area

**Fig. 7:**
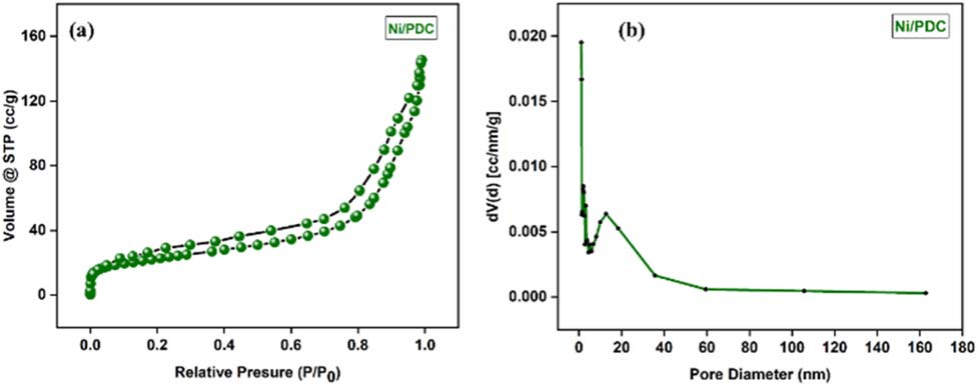
(a) BET surface area analysis and (b) pore size distribution of the Ni/PDC nanocomposite.

Based on the nitrogen adsorption–desorption study, the Ni/PDC catalyst shows a typical type IV isotherm with a noticeable hysteresis loop in the relative pressure (*P*/*P*_0_) region of 0.5–1.0 (Fig. 7), with the surface area of 80.9 m^2^ g^−1^. The BJH pore size distribution shows pore diameter of 9.56 nm which is typical of mesoporous materials according to IUPAC classification and having the pore volume of 0.219 cm^3^ g^−1^. The N2 sorption analysis shows that mesoporous nature with good surface area facilitate the active sites thereby increasing the catalytic activity. The porous network of the catalyst was also observed in TEM analysis.

### 3.5 X-ray photoelectron spectroscopy (XPS)

**Fig. 8:**
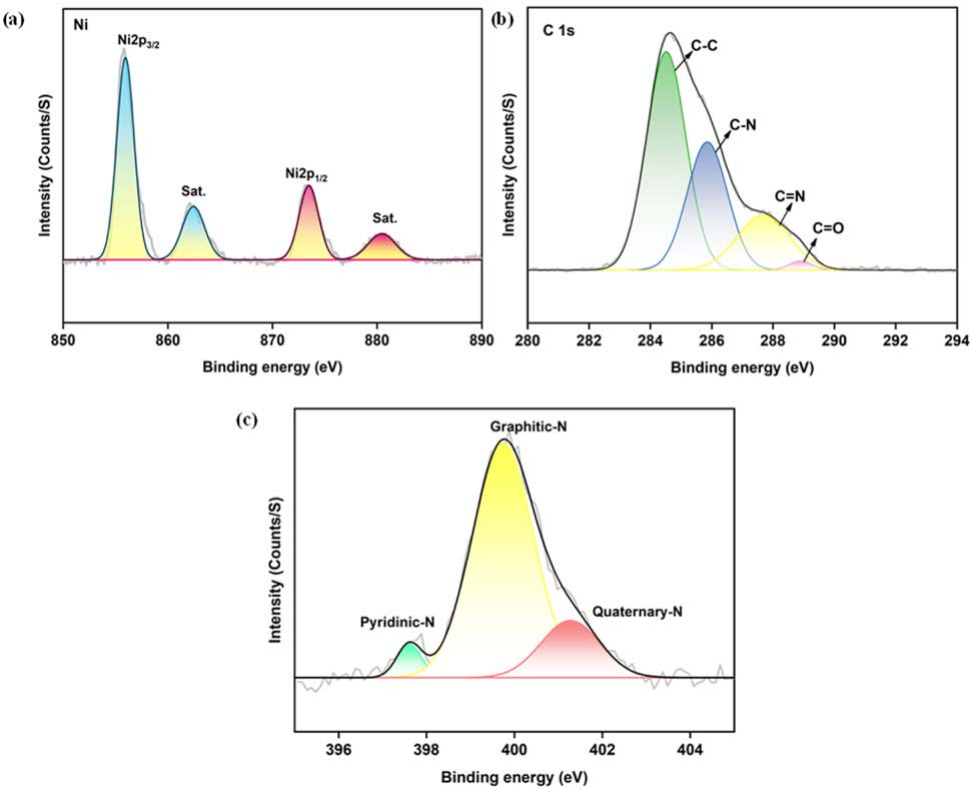
XPS analysis of Ni/PDC nanoparticles (a) Spectrum of Ni 2p (b) C 1s (c) N 1s.

X-ray photoelectron spectroscopy (XPS) was employed to investigate the surface elemental composition and chemical states of the Ni/PDC nanocomposite. The full survey spectrum(Fig. S1a, SI) confirms the successful incorporation of nickel nanoparticles onto the polyaniline-derived carbon support, validating the presence of Ni, C, N, and O components, which flawlessly corroborates the macroscopic elemental distribution observed in the EDX mapping. In the high-resolution Ni 2p spectrum (Fig. 8a), two notable peaks are observed at binding energies of 854.3 eV and 872.1 eV, which correspond to the Ni 2p_3/2_ and Ni 2p_1/2_ states, respectively, exhibiting a spin–orbit separation of approximately 27 eV. Furthermore, the presence of oxidized nickel compounds is identified by the distinct shake-up satellite peaks at 861.2 eV and 879.5 eV.^41,42^ As is typical for nickel-based catalysts exposed to air, these results indicate the presence of surface Ni^2+^ species resulting from the partial surface oxidation of the metallic nickel nanoparticles. Importantly, while XRD and SAED predominantly detected bulk metallic nickel (Ni^0^) the surface-sensitive nature of XPS reveals that these metallic cores are passivated by a thin, oxidized Ni^2+^ surface layer. The high-resolution C 1s spectrum (Fig. 8b) was deconvoluted into four components centered at 284.5, 285.8, 287.8, and 288.9 eV. These correspond to C–C/C=C, C–N, C=N and C=O bonds, respectively, confirming the successful formation of the nitrogen-doped carbon framework. To clarify the specific configurations of the nitrogen dopants, the high-resolution N 1s spectrum (Fig. 8c) was deconvoluted into three distinct peaks centered at 397.8 eV, 399.9 eV and 401.4 eV, which correspond to pyridinic-N, graphitic-N and quaternary-N, respectively. These distinct nitrogen configurations play targeted roles in the composite's catalytic performance. Specifically, pyridinic-N provides lone-pair electrons and defect sites that help anchor the Ni species and enhance metal-support interactions, while the dominant graphitic-N species substitutes carbon atoms within the framework to improve the intrinsic electrical conductivity and facilitate efficient charge transfer. Finally, the O 1s spectrum (Fig. S1b, see SI) was deconvoluted into two peaks at 531.7 eV and 536.2 eV, which are attributed to lattice oxygen/metal–oxygen species and surface-adsorbed hydroxyl groups or water molecules, respectively.^43,44^

### 3.6 Magnetic behavior of the Ni/PDC catalyst using a vibrating sample magnetometer (VSM)

**Fig. 9:**
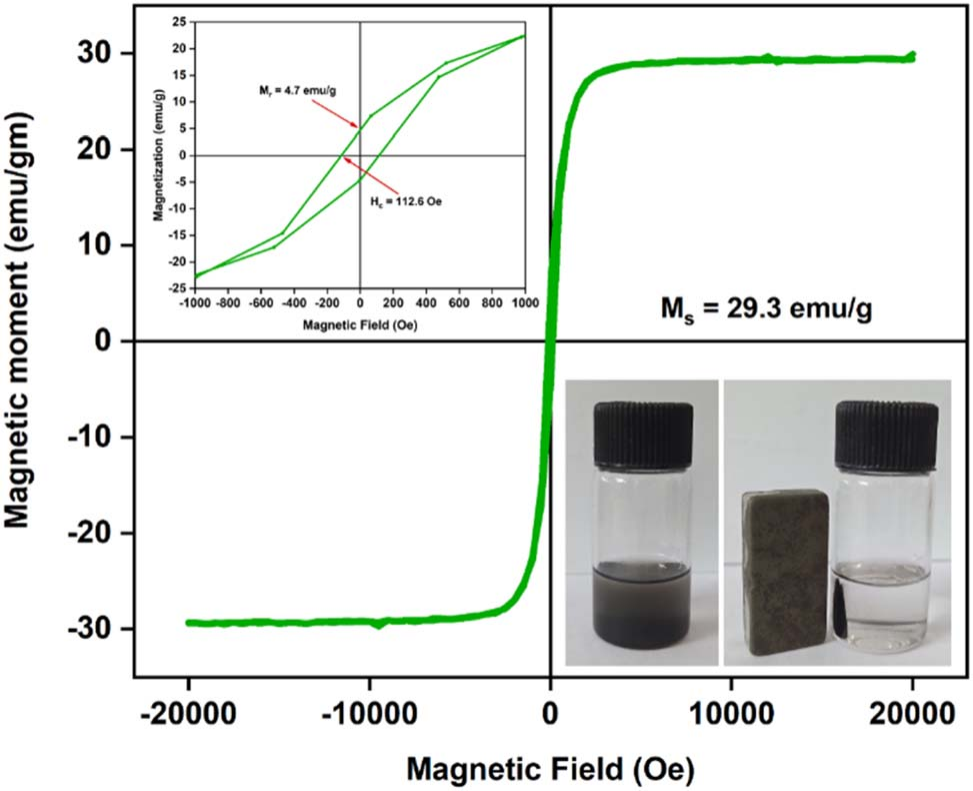
Magnetic hysteresis loop of the as-synthesized Ni/PDC.

The magnetic behavior of the as-synthesised Ni-PDC nanoparticles at room temperature was investigated using vibrating sample magnetometry (VSM). Fig. 9 displays the appropriate hysteresis loop. Soft ferromagnetic behavior is indicated by the distinctive S-shaped profile of the M–H curve. This ferromagnetic property is a direct functional consequence of the highly crystalline, fcc metallic Ni cores previously confirmed by XRD and TEM analysis. The hysteresis loop yielded the following magnetic parameters: a coercive field (Hc) of 112.6 Oe, a saturation magnetization (*M*_s_) of 29.3 emu g^−1^, and a remanent magnetization (*M*_r_) of 4.7 emu g^−1^. For effective magnetic separation and catalyst recovery following the reaction, the comparatively low coercivity and remanence imply simple magnetization and demagnetization.

### 3.7 Catalysis study

To spot an appropriate catalytic system, we started our investigation using 4-methoxybenzyl alcohol with Ni/PDC as the benchmark substrate for the catalytic oxidation of benzyl alcohol. At first, the synthesized Ni/PDC as the catalyst, molecular oxygen as a sole oxidant in acetonitrile at 70 °C. To our delight, the desired product (2a) was obtained in 82% yield (Table 1, entry 1). Further, we examine the various oxidants to improve the catalytic efficiency TBHP (in decane) rendered the desired product 2a in 76%, H_2_O_2_ and NaOCl delivered the desired product 2a in less yield (Table 1, entry 2–4). On screening the various solvents such as DMSO, DMF, methanol, ethanol and water yielded the desired product 2a in 53–78% yields (entries 5–9). Remarkably, while using the isopropanol as a solvent delivered the desired product 2a in maximum yield 93% (Table 1, entry 10). Other synthesized catalysts (Co/PDC, Mn/PDC) were also screened for the oxidation of benzyl alcohol; however, diminished yield was observed in compared with Ni/PDC (Table 1, entries 11 and12). Furthermore, the reaction was halted when the Ni precatalyst were employed instead of synthesized Ni/PDC (Table 1, entry 13). Supported Ni nanoparticles on conventional supports such as SiO_2_, Al_2_O_3_ and activated carbon (AC) were also prepared and evaluated under similar reaction conditions. These catalysts exhibited lower yields in benzaldehyde formation compared to the Ni/PDC catalyst (Table 1, entries 14–16). Crucially, the distinct drop-in catalytic activity observed for the nitrogen-free Ni/AC catalyst explicitly confirms that the incorporation of nitrogen into the carbon matrix is not merely structural. Instead, it plays a direct, promotional role in the catalytic process; as corroborated by our XPS analysis, the nitrogen species synergize with the Ni nanoparticles to enhance the overall catalytic performance. In the absence of catalyst and molecular oxygen, no desired product 2a was observed (Table 1, entries 17 and 18). Moreover, decrease the reaction temperature and shorter reaction time adversely affected the product yield (Table 1, entries 19–21). Furthermore, the influence of reaction time on catalytic performance was investigated under identical conditions. Increasing the reaction time to 24 h resulted in a significant decrease in benzaldehyde yield to 27% (Table 1, entry 22). This reduction is attributed to the overoxidation of benzaldehyde to benzoic acid, which was formed in 60% yield, as confirmed by NMR analysis. In contrast, the optimal reaction time of 6 h afforded a high benzaldehyde yield of 93%, indicating that prolonged reaction time promotes further oxidation of the desired product.

**Table 1:** Optimization of reaction condition for oxidation of benzyl alcohol^a^

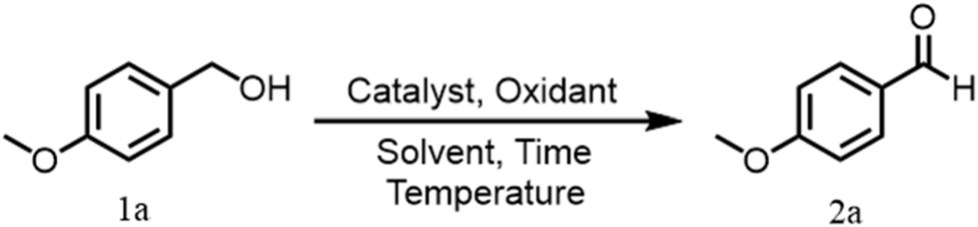				
Entry	Catalyst	Oxidant	Solvent	Yield^b^ (%)
1	Ni/PDC	O_2_	CH_3_CN	82
2	Ni/PDC	TBHP (in decane)	CH_3_CN	76
3	Ni/PDC	H_2_O_2_	CH_3_CN	51
4	Ni/PDC	NaOCl	CH_3_CN	28
5	Ni/PDC	O_2_	DMSO	53
6	Ni/PDC	O_2_	DMF	67
7	Ni/PDC	O_2_	MeOH	75
8	Ni/PDC	O_2_	EtOH	78
9	Ni/PDC	O_2_	Water	64
10	Ni/PDC	O_2_	Isopropanol	93
11	Co/PDC	O_2_	Isopropanol	71
12	Mn/PDC	O_2_	Isopropanol	68
13	Ni(NO_3_)_2_·6H_2_O	O_2_	Isopropanol	n.d
14	Ni/SiO_2_	O_2_	Isopropanol	69
15	Ni/Al_2_O_3_	O_2_	Isopropanol	56
16	Ni/AC	O_2_	Isopropanol	71
17	—	O_2_	Isopropanol	n.d
18	Ni/PDC	—	Isopropanol	n.d
19^c^	Ni/PDC	O_2_	Isopropanol	86
20^d^	Ni/PDC	O_2_	Isopropanol	38
21^e^	Ni/PDC	O_2_	Isopropanol	57
22^f^	Ni/PDC	O_2_	Isopropanol	27

a Reaction condition: benzyl alcohol 1a (0.5 mmol, 1 equiv.), oxidant (1.0 mmol, 2.0 equiv.), catalyst (15 mg), solvent (3 mL), temperature (70 °C) and time (6 h).

b Isolated yield.

c Reaction Proceeded at 50 °C.

d Reaction proceeded at room temperature.

e Reaction proceeded for 3 h.

f Reaction proceeded with 24 h. n.d-not detected.

### 3.8 CHEM21 green metrics

The findings of a systematic evaluation of the optimized catalytic protocol for benzyl alcohol oxidation using the CHEM21 green metrics toolkit are compiled in Table 2.^45–47^ Several previous studies have employed this toolkit to assess the sustainability and environmental impact of catalytic processes.^48–50^ The CHEM21 methodology's zero and first pass assessments have been carried out. The toolkit employs a traffic light method to classify processes: red highlights unacceptable features, amber suggests moderate concerns, and green indicates an acceptable process. The initial assessment focused on selectivity, yield, and conversion. The enhanced reaction performed very effectively, obtaining green flags for high yield (93%), conversion (94%), and selectivity (98.9%). Positive atom economy (A.E.), reaction mass efficiency (R.M.E.), and a relatively low process mass intensity (PMI), all of which are signs of material-efficient transformation, further reinforced the catalytic efficiency. In green chemistry, the choice of solvent is crucial. The CHEM21 solvent selection standards gave isopropanol, a renewable, low-toxicity, and biodegradable solvent, a green flag for conducting the process.

**Table 2:** CHEM21 green metrics using optimized method

Metric	Optimized reaction condition	Flag
Yield	93	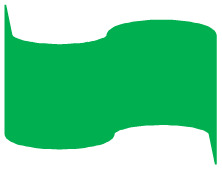
Conversion	94	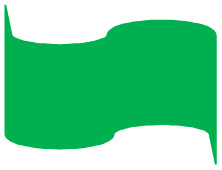
Selectivity	98.9	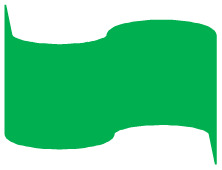
Atom economy	80	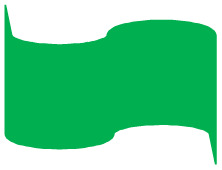
Reaction mass efficiency	91.6	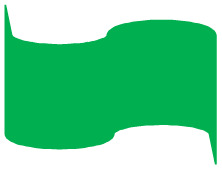
Process mass intensity	35.7	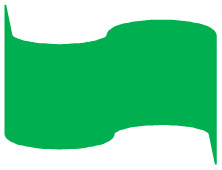
Solvent	Isopropanol	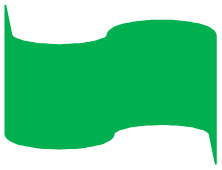
Catalyst	Yes	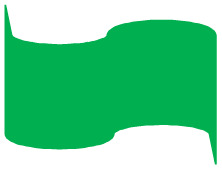
Catalyst recovery	Yes	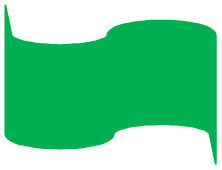
Element	Nickel	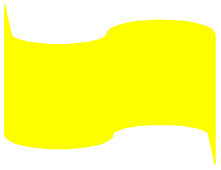
Reactor	Batch	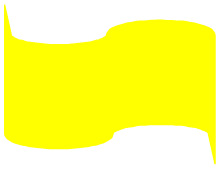
Workup	Column	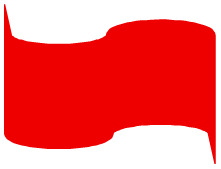
Energy	70 °C	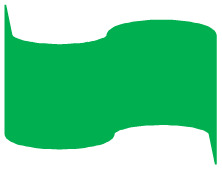
Health and safety	H315, H317, H319	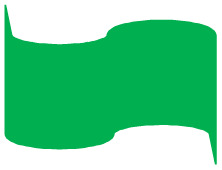

A heterogeneous, non-noble nickel catalyst employed in the process was effectively recovered and reused for seven cycles in a row with no activity loss. As a result, green flags received to both catalyst recovery and catalyst use. Due to global supply and geopolitical concerns, nickel obtains an amber flag under CHEM21 elemental sustainability criteria, despite being more affordable and abundant than noble metals.

Additionally, energy efficiency was assessed. Reactions carried out between 0 and 70 °C and at least 5 °C below the solvent boiling point are considered acceptable and given a green flag according to with CHEM21 rules. This requirement was satisfied because the oxidation was carried out at 70 °C, which is within the recommended temperature range. While column-based workup processes produced a red flag, which represented material and solvent usage during purification, the batch reactor format was given an amber flag. According to the CHEM21 evaluation criteria, the health and safety assessment revealed intermediate hazard ratings (H315, H317, and H319). (See the SI for detailed calculation data-excel sheets). Overall, the optimized protocol shows a positive green chemistry profile, despite the fact that several areas, such workup and elemental sustainability, require development. This is mainly due to the nickel heterogeneous catalyst's recyclability, high efficiency metrics, and usage of isopropanol as a green solvent.

**Fig. 10:**
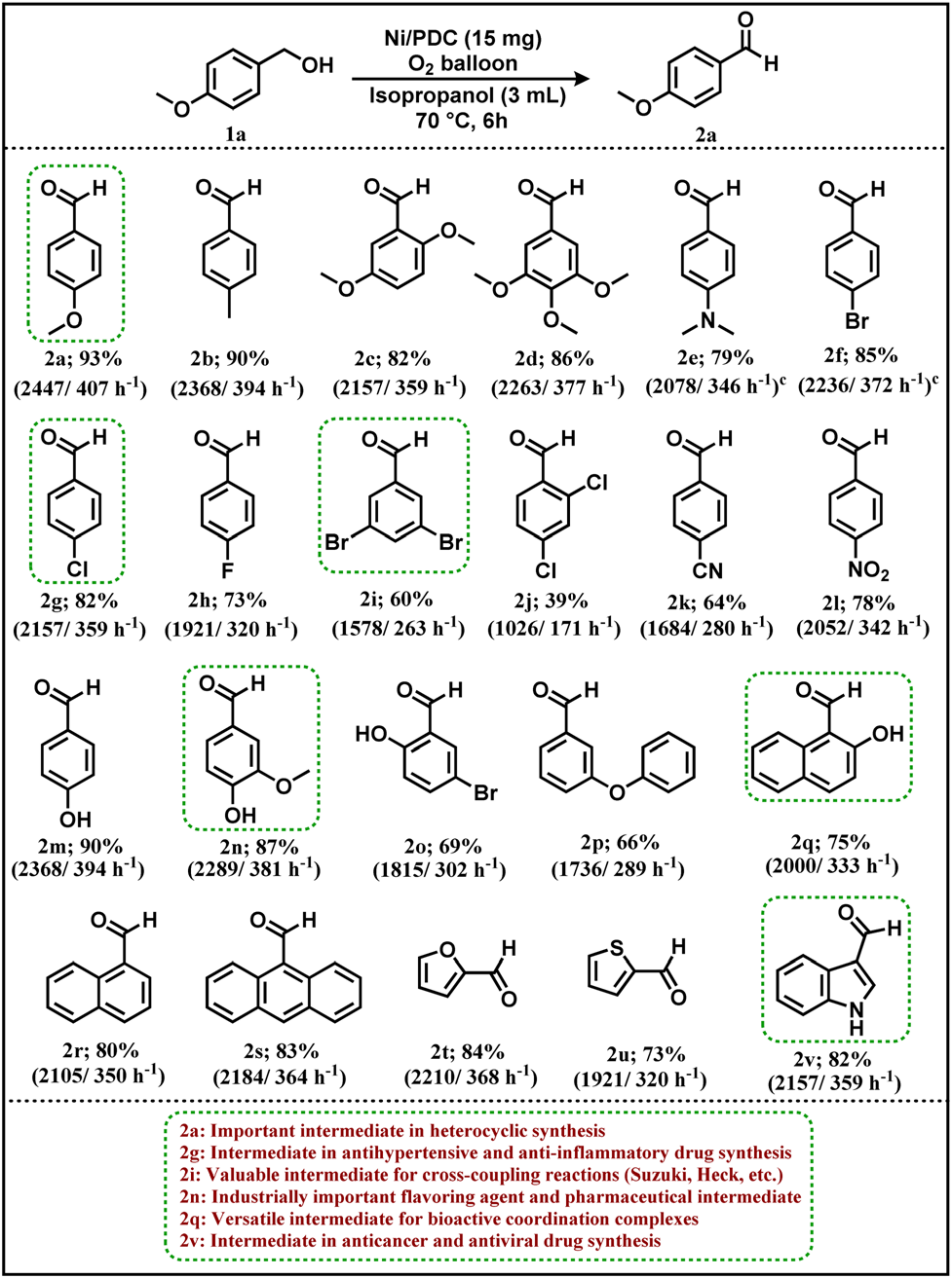
Substrate scope of benzyl alcohols.^*a*^ Reaction condition: benzyl alcohol 1a (0.5 mmol, 1 equiv.), Ni/PDC (15 mg), O_2_ balloon, Isopropanol (3 ml), 70 °C and 6 h.^*b*^ Isolated yield.^*c*^ Inside the parentheses is turnover number (TON)/turnover frequency (TOF).

Following the establishment of the ideal reaction conditions, a wide variety of benzyl alcohol substrates were investigated in order to look into the practical utility of the devised oxidation protocol, as shown in Fig. 10. Benzyl alcohols containing both electron-donating and electron-withdrawing substituents underwent an effortless transformation, yielding good to excellent yields (64–93%) of the corresponding benzaldehydes. Substrates containing alkyl, methoxy, hydroxy, amino, cyano, aldehyde, and halogen (F, Cl, and Br) functionalities were all well tolerated under the standard reaction conditions. Notably, benzyl alcohols possessing electron-rich substituents exhibited high reactivity. *Para*-methyl, *para*-methoxy, 2,5-dimethoxy, 1,2,3-trimethoxy, and *para*-dimethylamino substituted benzyl alcohols were efficiently converted to their respective aldehydes in excellent yields ranging from 82% to 93% (2a–2e). Benzyl alcohols substituted with mildly electron-withdrawing halogens at the *para* position also underwent clean oxidation, affording the desired products in very good yields (2f–2h). 4-Chlorobenzaldehyde (2g) is an important intermediate in the synthesis of pharmaceutical agents, heterocyclic compounds, and agrochemical products.^51^ Benzyl alcohols bearing multiple halogen substituents, such as 3,5-dibromo and 2,4-dichloro derivatives, were also efficiently oxidized under the optimized conditions to afford the corresponding aldehydes in good yields (2i, 2j). 3,5-Dibromobenzaldehyde (2i) is a versatile precursor for cross-coupling reactions and the construction of complex bioactive and functional organic molecules.^52^ Substrates containing moderately electron-withdrawing cyano groups (2k) as well as strongly electron-withdrawing nitro groups (2l) were successfully produced good to exceptional yields when oxidized to the respective aldehydes. The protocol demonstrated excellent functional group tolerance, as evidenced by the smooth oxidation of hydroxy-substituted benzyl alcohols, which furnished the desired aldehyde in 90% yield (2m). Importantly, the lignin-derived model compound vanillyl alcohol was effectively transformed into vanillin, highlighting the applicability of this methodology to biomass-related substrates (2n). Vanillin is an industrially important fine chemical widely used in the flavour and fragrance industry and also serves as a valuable intermediate in pharmaceutical and agrochemical synthesis.^53^ Sterically congested substrates were also compatible with the catalytic system. For instance, oxidation of 5-bromo-2-hydroxybenzyl alcohol proceeded efficiently to afford 5-bromo-2-hydroxybenzaldehyde, indicating that *ortho*-substitution does not adversely affect the catalytic performance (2o). Additionally, ether-containing benzyl alcohols such as 3-phenoxybenzyl alcohol were smoothly converted to the corresponding aldehyde, further demonstrating the versatility of the protocol (2p). In addition, polycyclic benzylic alcohols bearing hydroxyl functionality, such as 2-hydroxy-1-naphthylmethanol, were smoothly oxidized to afford 2-hydroxy-1-naphthaldehyde in good yield (2q). 2-Hydroxy-1-naphthaldehyde is a versatile synthetic intermediate extensively employed in the preparation of Schiff base ligands, bioactive coordination complexes, and functional organic materials.^54^ Furthermore, the reaction was compatible with various other polycyclic alcohols, delivering their corresponding aldehydes efficiently (2r and 2s).

To further assess the generality of the method, heteroaryl alcohols including furan-2-ylmethanol (2t), thiophen-2-ylmethanol (2u), and indole-3-methanol (2v) were subjected to the optimized conditions, affording the corresponding aldehydes in 84%, 73%, and 81% yields, respectively. Importantly, the developed protocol was successfully demonstrated on a gram scale. Oxidation of 4-methoxybenzyl alcohol (1a, 7.34 mmol, 1g) in the presence of 220 mg of Ni/PDC catalyst afforded the corresponding aldehyde (2a) in 77% isolated yield, highlighting the practical scalability of the catalytic system. In addition, a preliminary cost estimation revealed that the total material cost for the gram–scale reaction (1g scale) was approximately $2.10, corresponding to about $2.67 per gram of the isolated aldehyde product. This further highlight the economic viability of the developed Ni/PDC catalytic system. 4-Methoxybenzaldehyde (anisaldehyde) is widely utilized as a fragrance ingredient and as a key building block in the synthesis of heterocyclic and pharmaceutical compounds.^55^

**Scheme 2:**
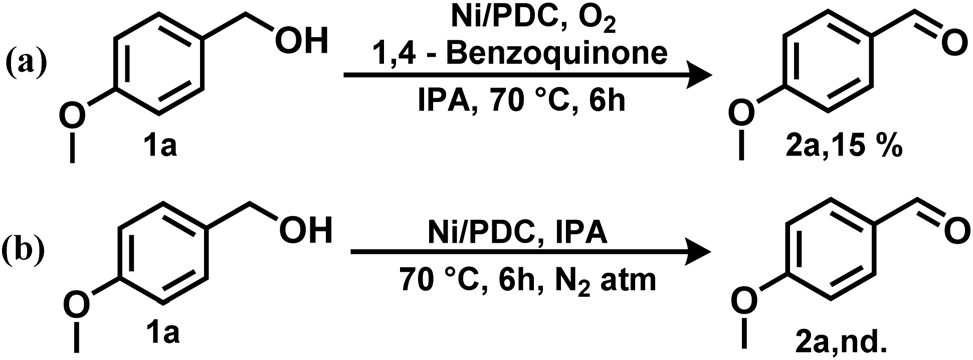
Control experiments for benzyl alcohol oxidation.

To elucidate the reaction pathway and identify the active species involved in the Ni/PDC catalyzed oxidation, control experiments were conducted (Scheme 2). When the oxidation of benzyl alcohol was performed under a nitrogen atmosphere instead of oxygen, no benzaldehyde product was detected (Scheme 2b). This confirms that molecular oxygen (O_2_) is the indispensable terminal oxidant for this catalytic system. Furthermore, to investigate the possible involvement of radical intermediates, the reaction was carried out in the presence of 1,4-benzoquinone (BQ), a well-known superoxide radical scavenger (Scheme 2a). The addition of BQ significantly suppressed the product yield to 15%. This strong inhibition indicates that reactive oxygen species, likely superoxide radicals (˙O_2_^−^), are generated by the activation of O_2_ on the catalyst surface and play a critical role in the catalytic cycle, presumably in the hydrogen abstraction or catalyst regeneration steps. Based on these experimental findings and previous literature, a refined mechanism is proposed.

**Scheme 3:**
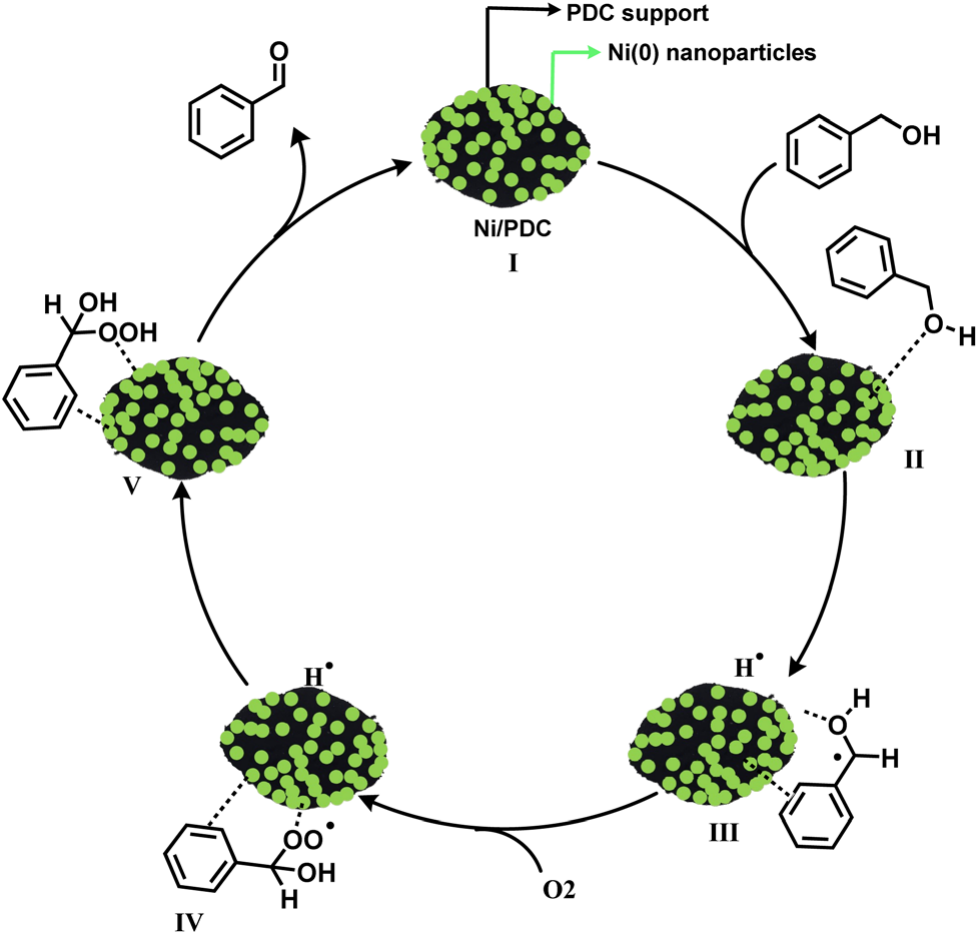
Plausible mechanism for the oxidation of benzyl alcohol to benzaldehyde.

Based on our preliminary experimental results and related reports,^56,57^ a radical-driven catalytic mechanism is proposed for the aerobic oxidation of benzyl alcohol over the Ni/PDC catalyst (Scheme 3). The catalytic cycle is initiated by the chemisorption of benzyl alcohol onto the active metallic nickel sites to form intermediate (INT II). This is followed by the abstraction of a benzylic hydrogen atom, generating a surface-bound benzyl radical species and a metal-hydride species (INT III). Subsequently, molecular oxygen (O_2_) reacts with the benzyl radical to form a peroxy radical intermediate (INT IV).

The formation of this crucial oxygen-centered radical intermediate is strongly supported by our control experiment, where the addition of a radical scavenger drastically reduced the reaction yield. This peroxy species then forms a hydroperoxide intermediate (INT V). Finally, the decomposition of INT V on the catalytic surface delivers the desired benzaldehyde product and water, thereby regenerating the active metallic nickel sites. The regenerated catalyst then re-enters the catalytic cycle to sustain continuous aerobic oxidation.

### 3.9 Recyclability study

**Fig. 11:**
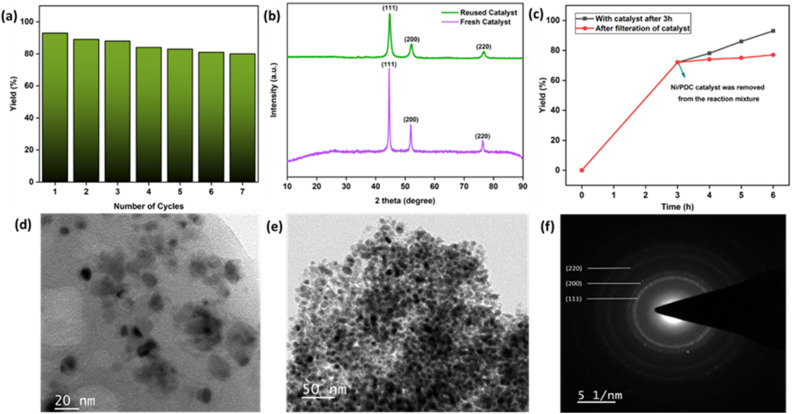
(a) Reusability of the Ni/PDC catalyst (b) fresh and reused PXRD (c) leaching experiment (d and e) TEM images (f) SAED pattern of reused Ni/PDC catalyst.

The ease of recovery and reusability of heterogeneous catalysts is a critical factor for sustainable applications. After the reaction, the magnetic Ni/PDC catalyst was easily extracted from the reaction mixture using an external magnet, washed multiple times with ethanol and water, dried, and reused in subsequent cycles. As shown in Fig. 11a, the catalyst exhibited outstanding stability over seven consecutive cycles, maintaining high catalytic activity with only a minor decrease in yield (from 93% to 80%).

To assess the structural integrity during reuse, the recovered catalyst was examined by XRD, TEM and SAED (Fig. 11b, d–f). The XRD pattern of the reused catalyst (Fig. 11b, green) shows diffraction peaks consistent with metallic Ni, with no significant narrowing or intensity increase compared to the fresh catalyst (Fig. 11b, purple). This confirms that the Ni nanoparticles do not undergo significant aggregation or re-precipitation into bulkier crystals during the reaction. The TEM and SAED images of the reused catalyst (Fig. 11d–f) further verify that the Ni nanoparticles remain well-dispersed and maintain their crystalline nature within the carbon framework. The minor yield decrease observed over seven cycles is likely attributed to minor surface passivation or structural evolution of the carbon support noted by the slight change in the amorphous XRD baseline which may partially block access to some active sites over time.

The heterogeneity of the system was confirmed *via* a hot filtration experiment (Fig. 11c). After 3 h of reaction (72% yield), the catalyst was magnetically removed, the resulting filtrate showed no further increase in yield, indicating that no active species remained in the solution. This was further corroborated by ICP-MS analysis of the reaction filtrate, which detected a negligible nickel concentration of only 7.221 ppb. These results underscore that the strong interaction between the Ni species and the polyaniline-derived carbon (PDC) support prevents metal leaching and ensures excellent recyclability for sustainable oxidation reactions.

**Fig. 12:**
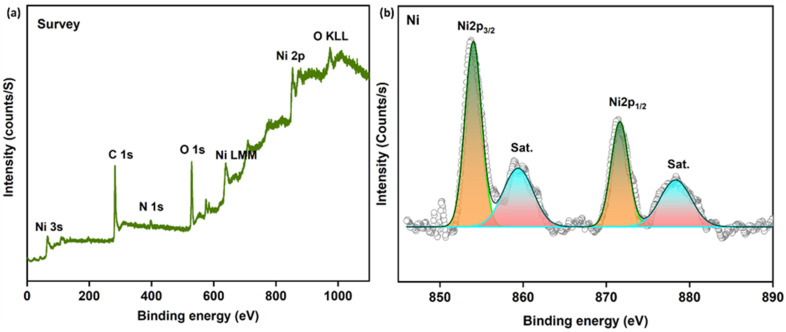
XPS analysis of the reused Ni/PDC catalyst: (a) survey spectrum (b) Ni 2p.

XPS was used to investigate the recycled Ni/PDC catalyst's surface composition (Fig. 12). After several catalytic cycles, the elemental composition does not change, as the survey spectrum demonstrates that Ni, C, N, and O elements are present. The coexistence of metallic Ni^0^ and Ni^2+^ species is confirmed by the high-resolution Ni 2p spectrum, which displays distinctive Ni 2p_3/2_ and Ni 2p_1/2_ peaks along with associated satellite characteristics. These nickel species are retained following recycling, indicating the catalyst's structural stability and supporting its long-term catalytic activity.

XPS analysis reveals that the atomic concentrations of the fresh and recycled catalysts are similar. The atomic concentration values obtained from XPS examination, which demonstrate no discernible change in surface composition after recycling, validate the stability of the Ni/PDC catalyst. There was a slight decrease in nickel concentration and a slight increase in surface carbon content, which might be explained by trace adsorption of organic molecules or slight surface modification during the catalytic process. Overall, the catalyst's stability under the reaction circumstances is demonstrated by the small shift in elemental composition (Table 3).

**Table 3:** Surface atomic composition (at%) of fresh and reused Ni/PDC catalysts determined by XPS

	Fresh catalyst (atom%)	Reused catalyst (atom%)
Ni 2p	8.5	8.1
C 1s	72.3	73.0
N 1s	12.4	11.9
O 1s	6.8	7.0

## 4 Conclusion

In summary, a magnetically recoverable Ni/PDC nanocatalyst was prepared using a polyaniline-derived carbon support, generating a nitrogen-rich mesoporous carbon framework with uniformly distributed nickel species. Structural and surface analyses confirmed the presence of both metallic Ni^0^ and Ni^2+^ species. Magnetic measurements indicated that the bulk Ni^0^ phase contributes to the ferromagnetic nature of the material, enabling convenient separation of the catalyst after reaction. The catalyst exhibited good activity for the aerobic oxidation of benzyl alcohol derivatives under mild and environmentally friendly conditions, affording high product yields for a range of substrates. The use of molecular oxygen as a green oxidant, together with a recyclable magnetic catalyst system, makes this protocol practically attractive. The catalyst maintained its performance over several reaction cycles, and its effectiveness was also demonstrated on a gram scale. These findings highlight the potential of nitrogen-doped carbon-supported nickel catalysts as cost-effective alternatives for sustainable oxidation transformations.

## Author contributions

K. Santhakumar: investigation, project administration, writing-review & editing, supervision. Dinesh babu Arumugam: conceptualization, methodology, writing – original draft.

## Conflicts of interest

The authors declare no conflicts of interest.

## Supplementary Material

RA-016-D6RA02052F-s001

## Data Availability

The data supporting this article have been included as part of the supplementary information (SI). Supplementary information: spectral data, NMR spectra of synthesized compounds and excel sheet of CHEM21 green metrics toolkit. See DOI: https://doi.org/10.1039/d6ra02052f.
