# Supplementary material for: Efficient aerobic oxidation of benzyl alcohols using a magnetically recoverable Ni/nitrogen-containing carbon nanocomposite
Source: RSC Adv. 2026 May 21;16(28):25806–18. doi: 10.1039/d6ra02052f (PMC13195421; doi:10.1039/d6ra02052f)
Supplement: RA-016-D6RA02052F-s001 [file RA-016-D6RA02052F-s001.pdf]

Supplementary Information (SI) for RSC Advances.
This journal is © The Royal Society of Chemistry 2026

Supporting Information

Efficient Aerobic Oxidation of Benzyl Alcohols Using a Magnetically Recoverable Ni/Nitrogen-Containing Carbon Nanocomposite

Arumugam Dinesh Babu [a] and K. Santhakumar *[a]

[a]Department of Chemistry, School of Advanced Sciences, Vellore Institute of Technology, Vellore - 632014, India.

Email: ksanthakumar@vit.ac.in

Table of contents

1. XPS spectra	S3
2. NMR Spectral data	S3
3. NMR spectra of synthesized compounds	S7
4. Zero Pass of optimized condition CHEM21 green metrics toolkit	S29
5. First Pass of optimized condition CHEM21 green metrics toolkit	S30
6. References	S32

1. XPS spectra

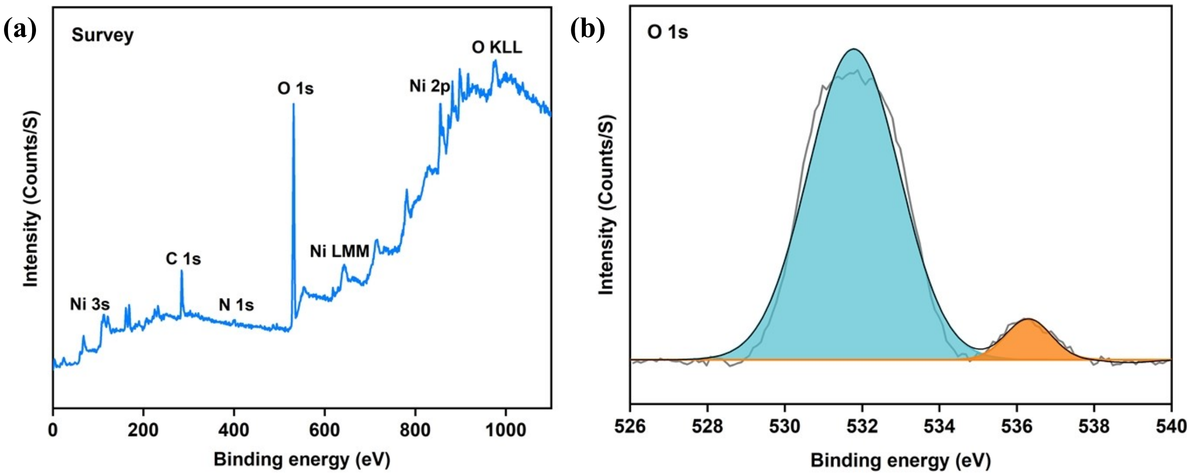

Fig. S1 (a) XPS survey and (b) O1s of Ni/PDC catalyst.

1. NMR Spectral data

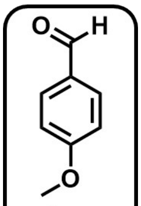
2a

4-methoxybenzaldehyde (2a): ^{1}H NMR (400 MHz, $CDCl_3$) δ 9.89 (s, 1H), 7.85 (d, *J* = 8.4 Hz, 2H), 7.01 (d, *J* = 8.4 Hz, 2H), 3.90 (s, 3H). **^{13}C NMR (101 MHz, $CDCl_3$)** δ 190.87, 164.63, 132.02, 129.98, 114.33, 55.61.[1]

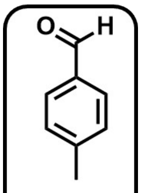
2b

4-methylbenzaldehyde (2b): ^{1}H NMR (400 MHz, $CDCl_3$) δ 9.96 (s, 1H), 7.78 (d, *J* = 8.0 Hz, 2H), 7.33 (d, *J* = 7.8 Hz, 2H), 2.44 (s, 3H). **^{13}C NMR (101 MHz, $CDCl_3$)** δ 192.94, 145.56, 134.08, 129.12, 129.05, 129.00, 128.94, 128.89, 22.53.[1]

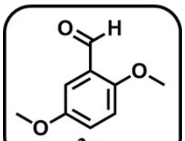
2c

2,5-dimethoxybenzaldehyde (2c): ^{1}H NMR (400 MHz, $CDCl_3$) δ 10.45 (s, 1H), 7.33 (d, *J* = 3.3 Hz, 1H), 7.14 (dd, *J* = 9.1, 3.3 Hz, 1H), 6.95 (d, *J* = 9.0 Hz, 1H), 3.90 (s, 3H), 3.81 (s, 3H). **^{13}C NMR (101 MHz, $CDCl_3$)** δ 189.63, 156.74, 153.61, 124.93, 123.54, 113.36, 110.40, 56.18, 55.84.[2]

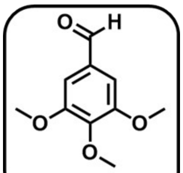
2d

3,4,5-trimethoxybenzaldehyde (2d): ^{1}H NMR (400 MHz, $CDCl_3$) δ 9.88 (t, *J* = 1.9 Hz, 1H), 7.14 (d, *J* = 2.1 Hz, 2H), 3.94 (d, *J* = 2.6 Hz, 9H). **^{13}C NMR (101 MHz, $CDCl_3$)** δ 191.08, 153.66, 143.60, 131.72, 106.71, 61.01, 56.28.

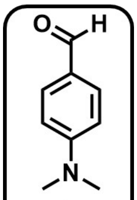
2e

4-(dimethylamino)benzaldehyde (2e): ^{1}H NMR (500 **MHz, CDCl$_3$)** δ 9.74 (s, 1H), 7.73 (d, *J* = 8.9 Hz, 2H), 6.70 (d, *J* = 8.7 Hz, 2H), 3.08 (s, 6H).**^{13}C NMR (101 MHz, CDCl$_3$)** δ 190.4, 154.4, 132.0, 125.1, 111.1, 40.1.[3]

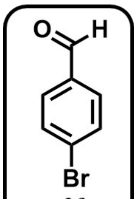

2f

4-bromobenzaldehyde (2f): ^{1}H NMR (400 MHz, CDCl$_3$) δ 9.98 (s, 1H), 7.76 (d, *J* = 8.5 Hz, 2H), 7.69 (d, *J* = 8.5 Hz, 2H). **^{13}C NMR (101 MHz, CDCl$_3$)** δ 191.14, 135.07, 132.47, 131.00, 129.82.[3]

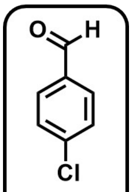

2g

4-chlorobenzaldehyde (2g): ^{1}H NMR (400 MHz, CDCl$_3$) δ 9.99 (s, 1H), 7.83 (d, *J* = 8.4 Hz, 2H), 7.53 (d, *J* = 8.2 Hz, 2H). **^{13}C NMR (101 MHz, CDCl$_3$)** δ 190.93, 141.00, 134.71, 130.94, 129.49.[1]

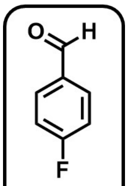

2h

4-fluorobenzaldehyde (2h): ^{1}H NMR (400 MHz, CDCl$_3$) δ 9.94 (s, 1H), 7.89 (dd, *J* = 8.8, 5.4 Hz, 2H), 7.18 (t, *J* = 8.6 Hz, 2H). **^{13}C NMR (101 MHz, CDCl$_3$) δ** 190.60, 167.78, 165.23, 132.28, 132.19, 116.43, 116.21.[4]

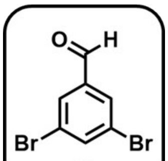

2i

3,5-dibromobenzaldehyde (2i): ^{1}H NMR (400 MHz, CDCl$_3$) δ 9.91 (s, 1H), 7.94 (d, *J* = 1.8 Hz, 2H), 7.93 – 7.91 (m, 1H). **^{13}C NMR (101 MHz, CDCl$_3$)** δ 189.22, 139.60, 138.89, 131.89, 131.22, 123.95, 123.16.

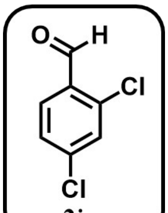

2j

2,4-dichlorobenzaldehyde (2j): ^{1}H NMR (400 MHz, CDCl$_3$) δ 10.43 (s, 1H), 7.89 (d, *J* = 8.3 Hz, 1H), 7.50 (s, 1H), 7.40 (ddd, *J* = 8.3, 2.0, 0.9 Hz, 1H). **^{13}C NMR (101 MHz, CDCl$_3$)** δ 188.55, 141.13, 138.56, 130.94, 130.48, 130.34, 127.98.

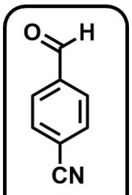

2k

4-formylbenzonitrile (2k): ^{1}H NMR (400 MHz, CDCl$_3$) δ 10.12 (s, 1H), 8.03 – 8.00 (m, 2H), 7.89 – 7.82 (m, 2H). **^{13}C NMR (101 MHz, CDCl$_3$)** δ 190.65, 138.75, 132.92, 129.91, 117.73, 117.62.[4]

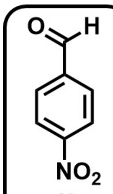

2l

4-nitrobenzaldehyde (2l): ^{1}H NMR (400 MHz, $CDCl_3$) δ 10.17 (s, 1H), 8.41 (d, *J* = 8.3 Hz, 2H), 8.09 (d, *J* = 8.3 Hz, 2H). **^{13}C NMR (101 MHz, $CDCl_3$)** δ 190.32, 140.05, 130.51, 124.34.[3]

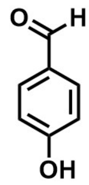

2m

4-hydroxybenzaldehyde (2m): ^{1}H NMR (400 MHz, $CDCl_3$) δ 9.89 (s, 1H), 7.87 – 7.83 (m, 2H), 7.03 – 6.99 (m, 2H), 6.46 (s, 1H). **^{13}C NMR (101 MHz, $CDCl_3$)** δ 191.36, 161.68, 132.58, 129.82, 116.05.[5]

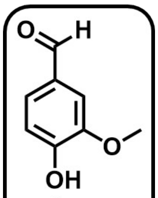

2n

4-hydroxy-3-methoxybenzaldehyde (2n): ^{1}H NMR (400 MHz, $CDCl_3$) δ 9.84 (s, 1H), 7.44 (dt, *J* = 6.8, 0.9 Hz, 2H), 7.06 (dd, *J* = 8.6, 1.1 Hz, 1H), 6.50 – 6.34 (m, 1H), 3.97 (d, *J* = 1.9 Hz, 3H). **^{13}C NMR (101 MHz, $CDCl_3$)** δ 191.02, 151.78, 147.21, 129.84, 127.59, 114.45, 108.82, 56.13.[6]

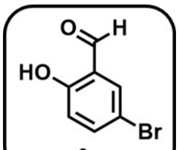

2o

5-bromo-2-hydroxybenzaldehyde (2o): ^{1}H NMR (400 MHz, $CDCl_3$) δ 10.94 (s, 1H), 9.85 (s, 1H), 7.68 (d, *J* = 2.2 Hz, 1H), 7.61 (dd, *J* = 8.9, 2.4 Hz, 1H), 6.91 (d, *J* = 8.9 Hz, 1H). **^{13}C NMR (101 MHz, $CDCl_3$)** δ 195.47, 160.58, 139.73, 135.66, 121.75, 119.84, 111.39.

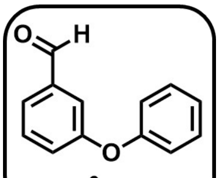

2p

3-phenoxybenzaldehyde (2p): 1H NMR (500 MHz, CDCl3) δ 9.97 (s, 1H), 7.62 (d, J = 7.5 Hz, 1H), 7.55 – 7.46 (m, 2H), 7.42 – 7.37 (m, 2H), 7.31 (dt, J = 8.0, 1.3 Hz, 1H), 7.22 – 7.16 (m, 1H), 7.07 (d, J = 8.2 Hz, 2H). **^{13}C NMR (101 MHz, $CDCl_3$)** δ 191.66, 158.43, 156.22, 138.09, 130.46, 130.07, 124.72, 124.60, 124.21, 119.51, 118.15.

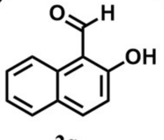

2q

2-hydroxy-1-naphthaldehyde (2q): ^{1}H NMR (400 MHz, $CDCl_3$) δ 13.16 (s, 1H), 10.80 (s, 1H), 8.33 (d, *J* = 8.5 Hz, 1H), 7.97 (d, *J* = 9.1 Hz, 1H), 7.79 (dd, *J* = 8.1, 1.3 Hz, 1H), 7.64 – 7.58 (m, 1H), 7.43 (t, *J* = 7.7 Hz, 1H), 7.13 (d, *J* = 9.1 Hz, 1H). **^{13}C NMR (101 MHz, $CDCl_3$)** δ 193.31, 164.97, 139.19, 132.91, 129.51, 129.15, 127.83, 124.53, 119.21, 118.62, 111.30.

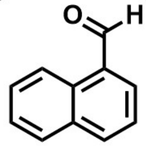

2r

1-naphthaldehyde (2r): 1H NMR (500 MHz, CDCl3) δ 10.32 (s, 1H), 9.26 (d, *J* = 8.6 Hz, 1H), 7.97 (d, *J* = 8.4 Hz, 1H), 7.88 – 7.81 (m, 2H), 7.67 – 7.61 (m, 1H), 7.56 – 7.47 (m, 2H). **^{13}C NMR (101 MHz, $CDCl_3$)** δ 193.60, 136.71, 135.33, 133.74, 131.41, 130.55, 129.10, 128.51, 126.99, 124.90.[1]

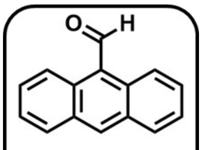

2s

anthracene-9-carbaldehyde (2s): ^{1}H NMR (500 MHz, $CDCl_3$) δ 11.48 (s, 1H), 8.96 (d, *J* = 9.0 Hz, 2H), 8.61 (s, 1H), 8.02 (d, *J* = 8.5 Hz, 2H), 7.69 – 7.64 (m, 2H), 7.56 – 7.51 (m, 2H). **^{13}C NMR (101 MHz, $CDCl_3$)** δ 192.99, 135.22, 132.11, 131.04, 129.28, 129.11, 125.67, 124.66, 123.52.[3]

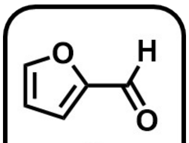

2t

furan-2-carbaldehyde (2t): ^{1}H NMR (400 MHz, $CDCl_3$) δ 9.67 (s, 1H), 7.71 (s, 1H), 7.27 (d, *J* = 3.6 Hz, 1H), 6.66 – 6.58 (m, 1H). **^{13}C NMR (101 MHz, $CDCl_3$)** δ 177.94, 152.97, 148.13, 121.12, 112.62.[3]

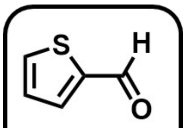

2u

thiophene-2-carbaldehyde (2u): ^{1}H NMR (400 MHz, $CDCl_3$) δ 9.95 (s, 1H), 7.82 – 7.75 (m, 2H), 7.25 – 7.20 (m, 1H). **^{13}C NMR (101 MHz, $CDCl_3$)** δ 183.09, 144.01, 136.43, 135.20, 128.37.[1]

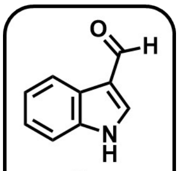

2v

1H-indole-3-carbaldehyde (2v): ^{1}H NMR (400 MHz, DMSO) δ 9.94 (s, 1H), 8.29 (s, 1H), 8.10 (d, *J* = 7.9 Hz, 1H), 7.52 (d, *J* = 8.3 Hz, 1H), 7.29 – 7.20 (m, 2H). **^{13}C NMR (101 MHz, DMSO)** δ 185.39, 139.00, 137.62, 124.63, 123.89, 122.56, 121.28, 118.63, 112.93.

2. NMR Spectra of synthesized compounds

^{1}H NMR spectra of product 2a

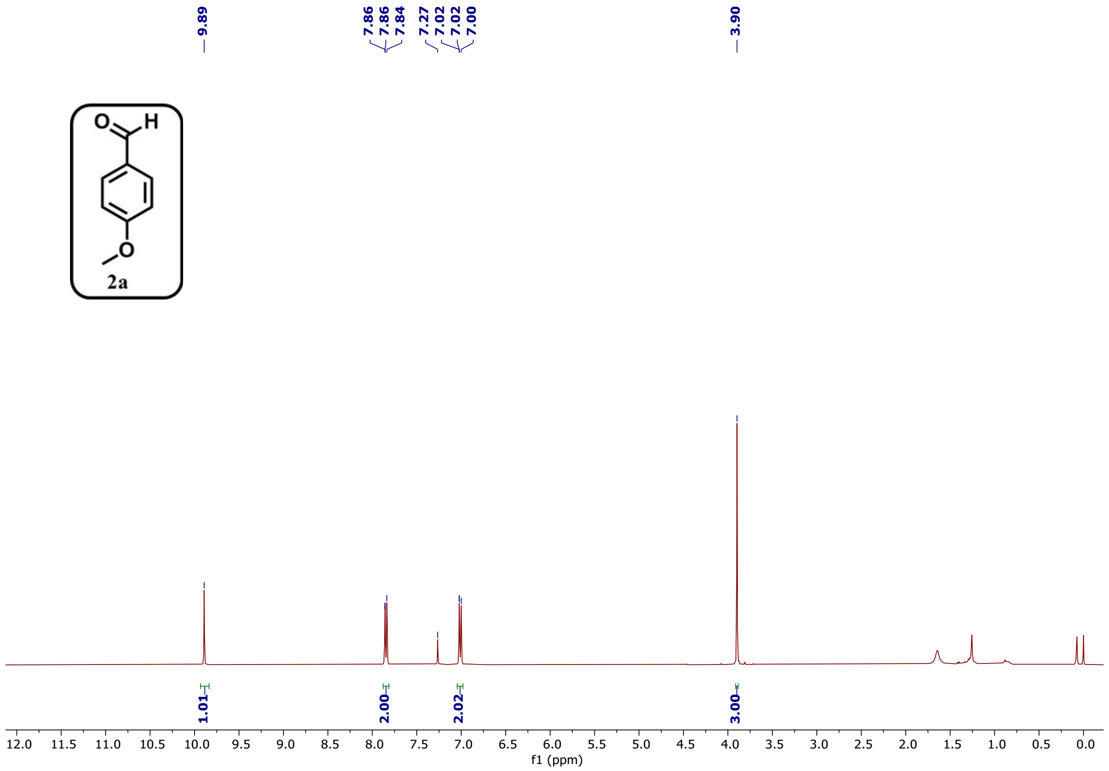

^{13}C NMR spectra of product 2a

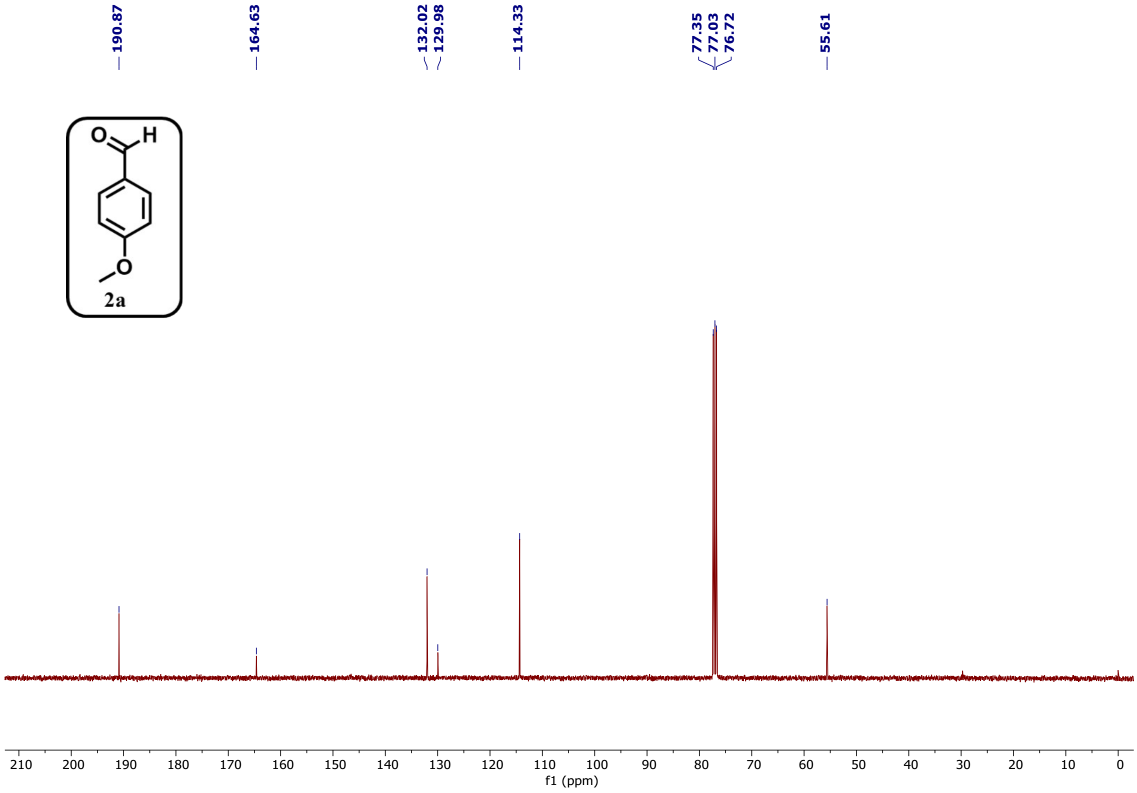

^{1}H NMR spectra of product 2b

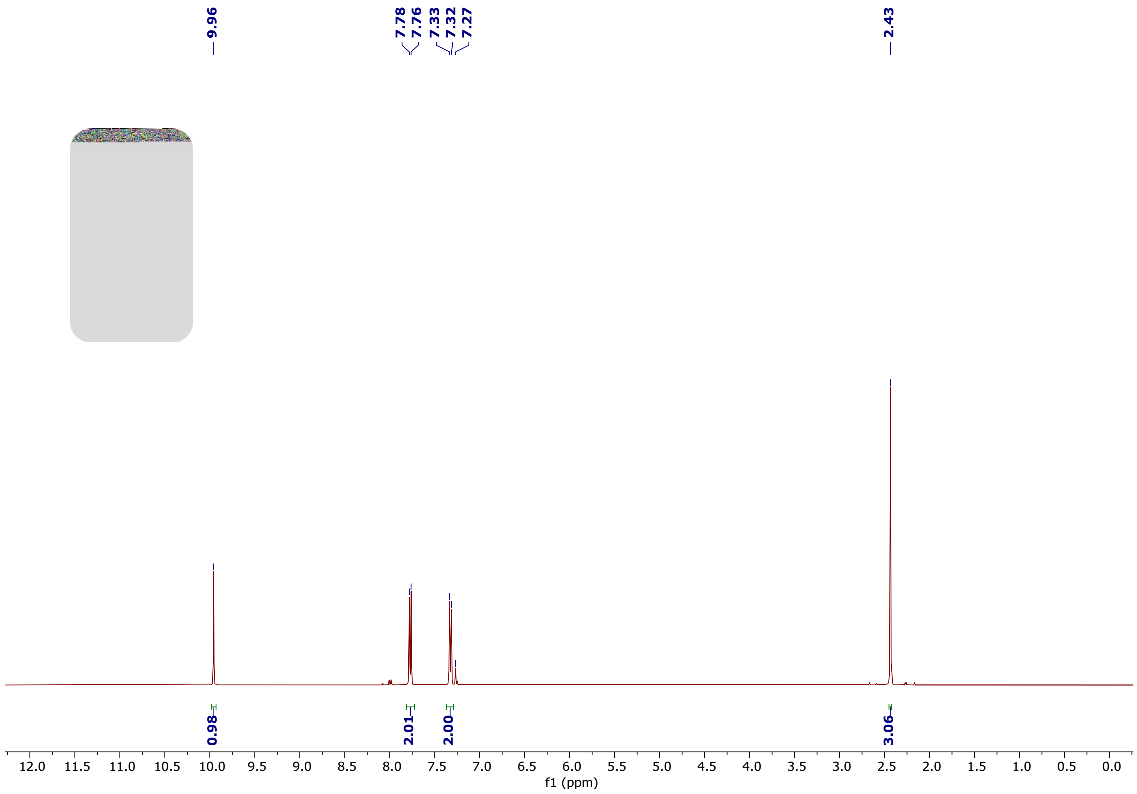

^{13}C NMR spectra of product 2b

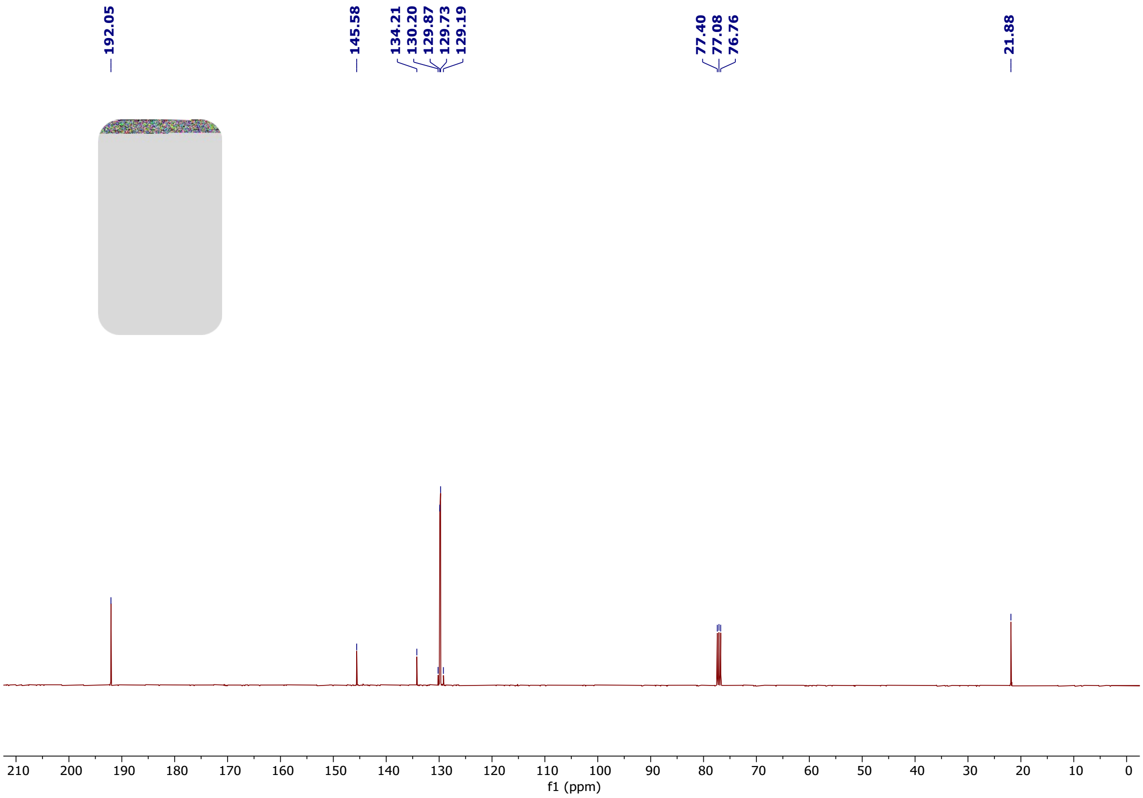

^{1}H NMR spectra of product 2c

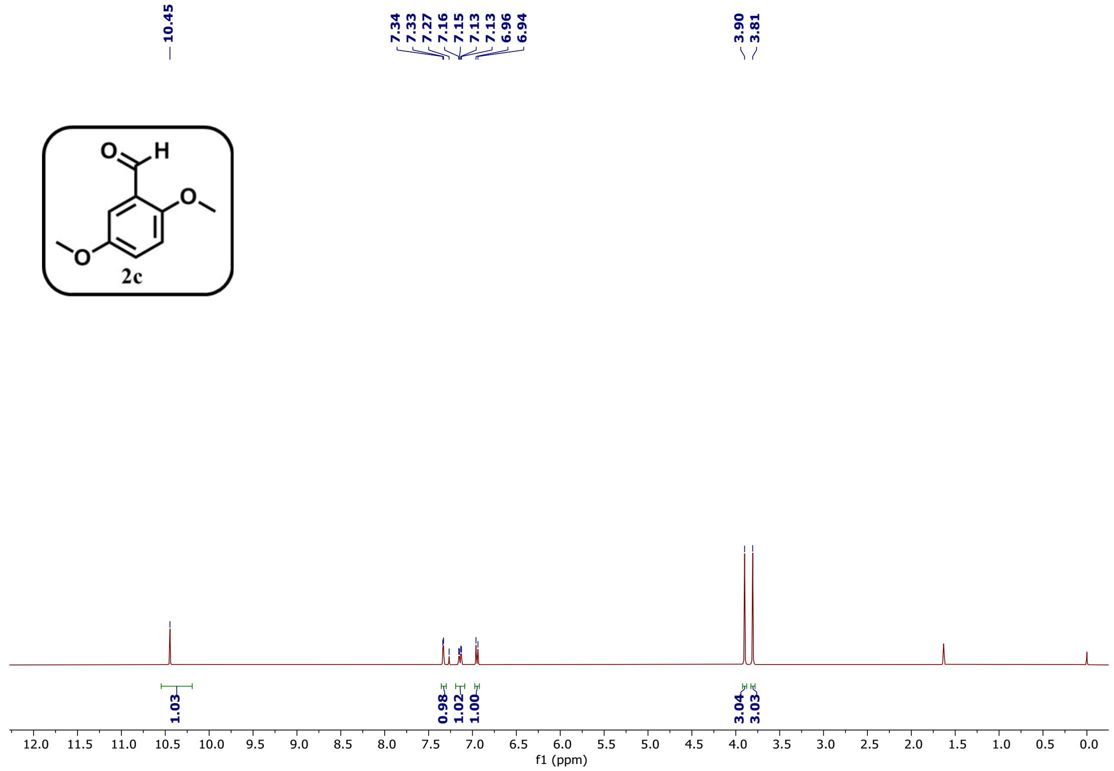

^{13}C NMR spectra of product 2c

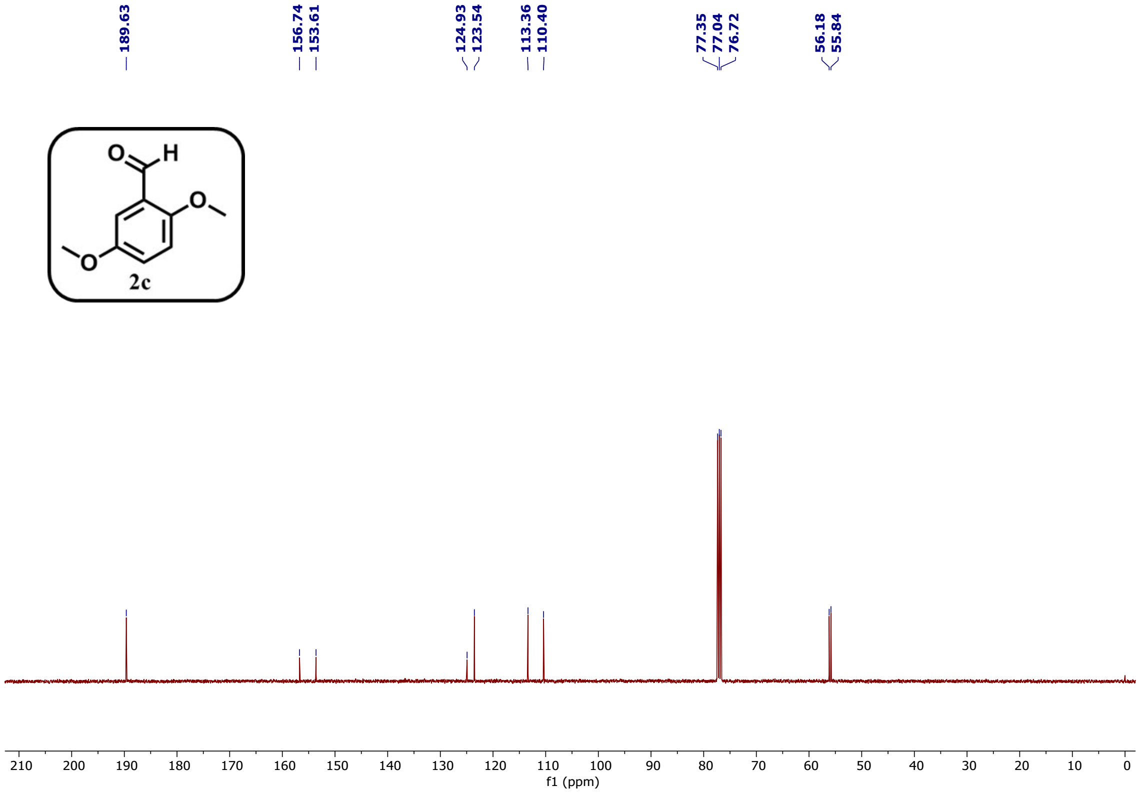

^{1}H NMR spectra of product 2d

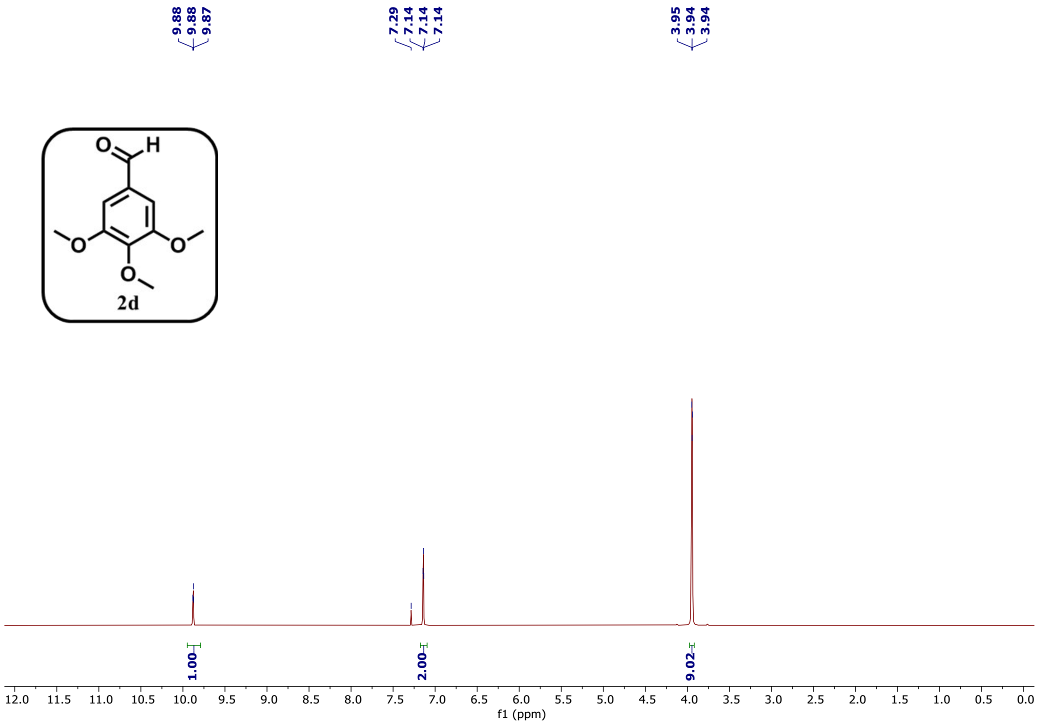

^{13}C NMR spectra of product 2d

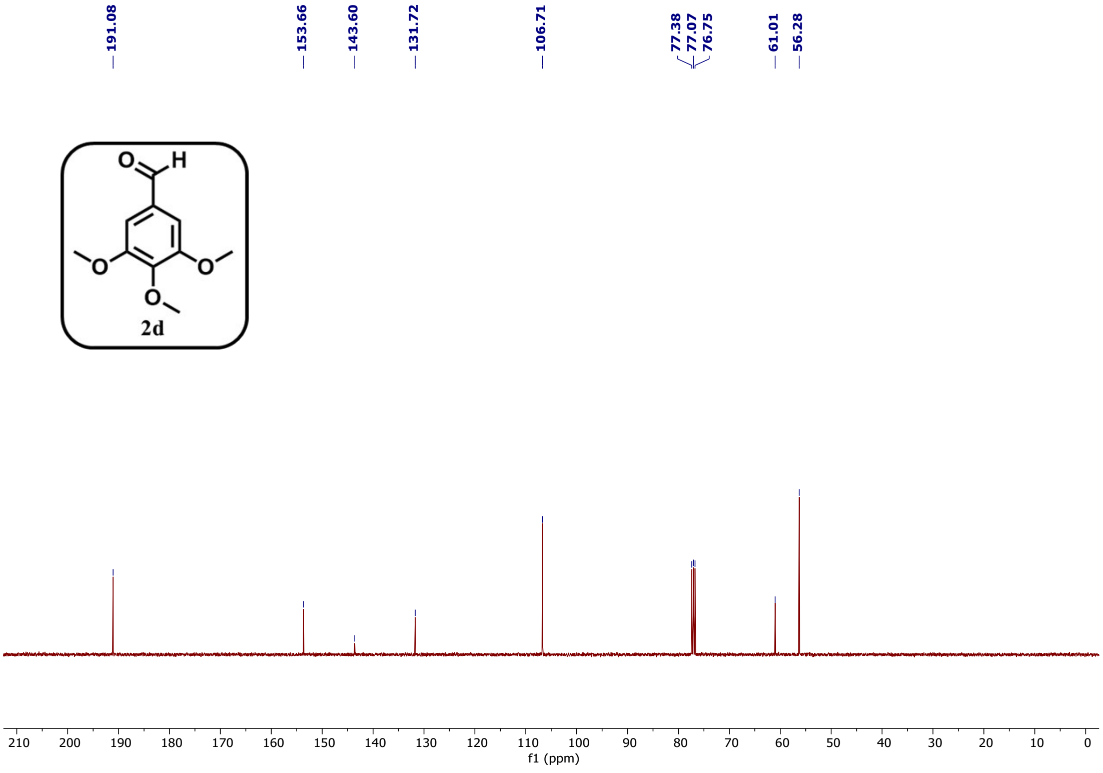

^{1}H NMR spectra of product 2e

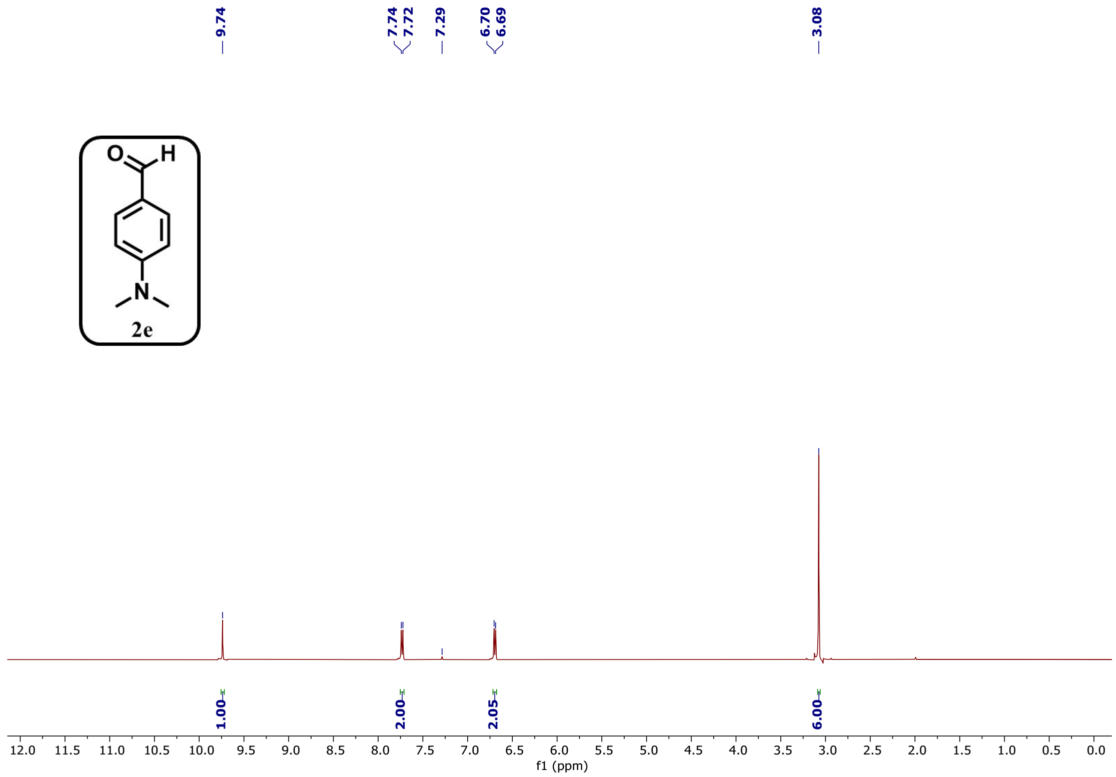

^{13}C NMR spectra of product 2e

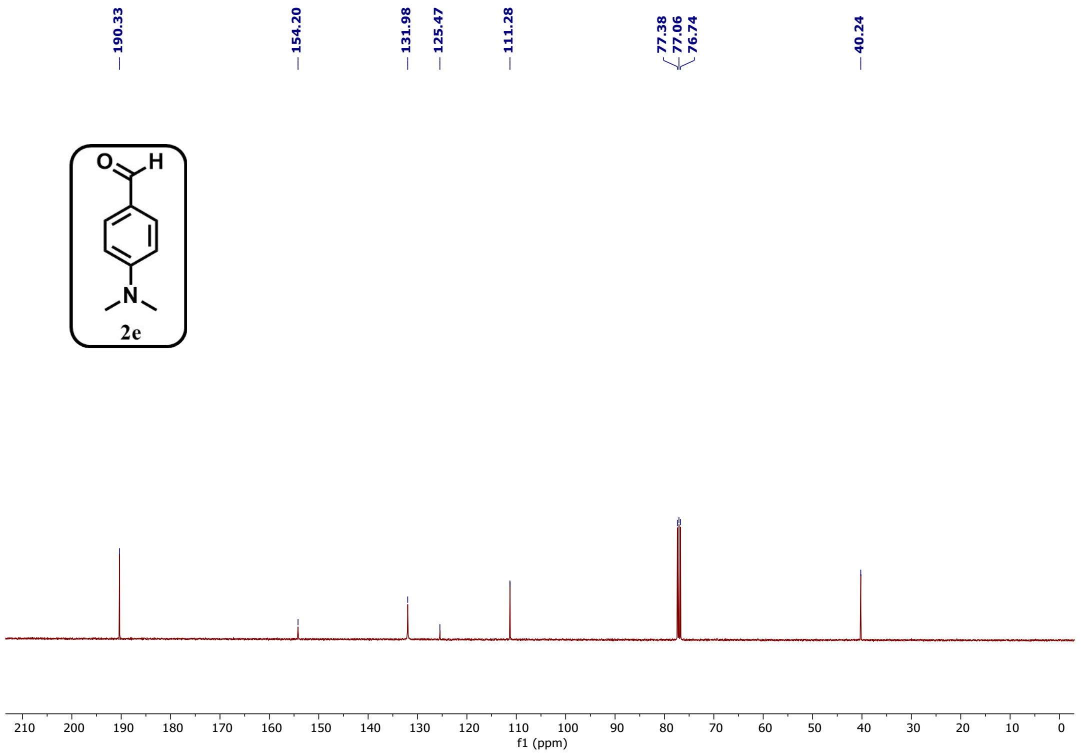

^{1}H NMR spectra of product 2f

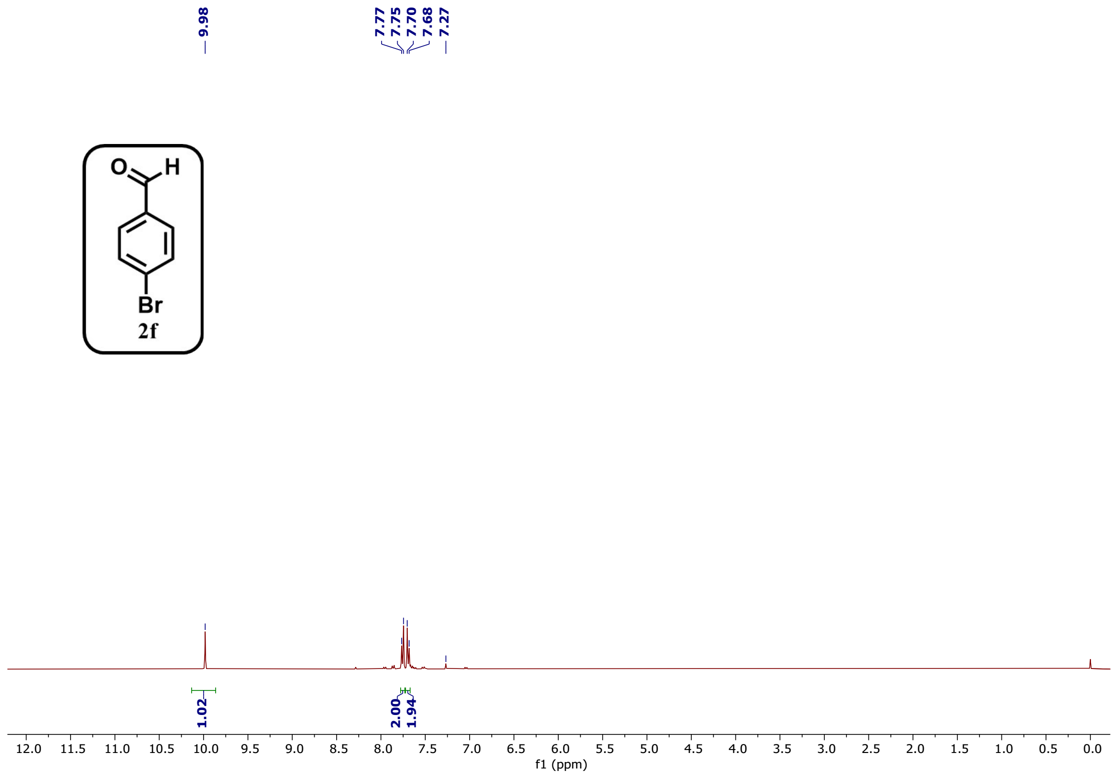

^{13}C NMR spectra of product 2f

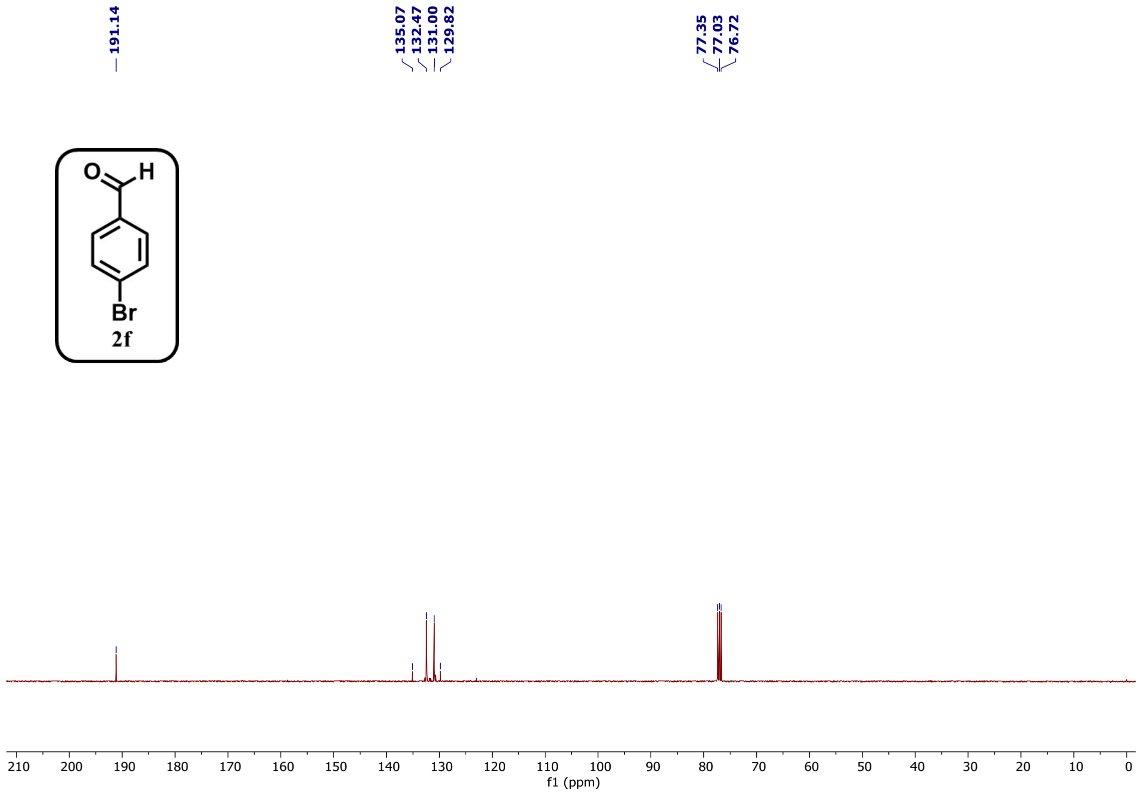

^{1}H NMR spectra of product 2g

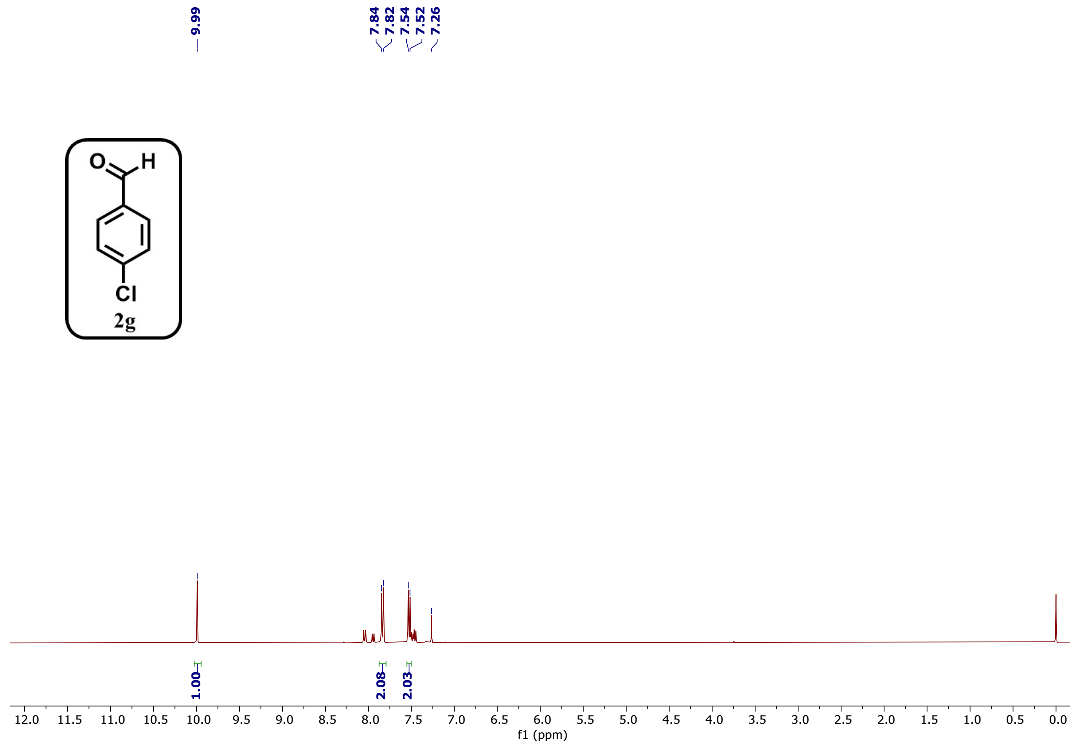

^{13}C NMR spectra of product 2g

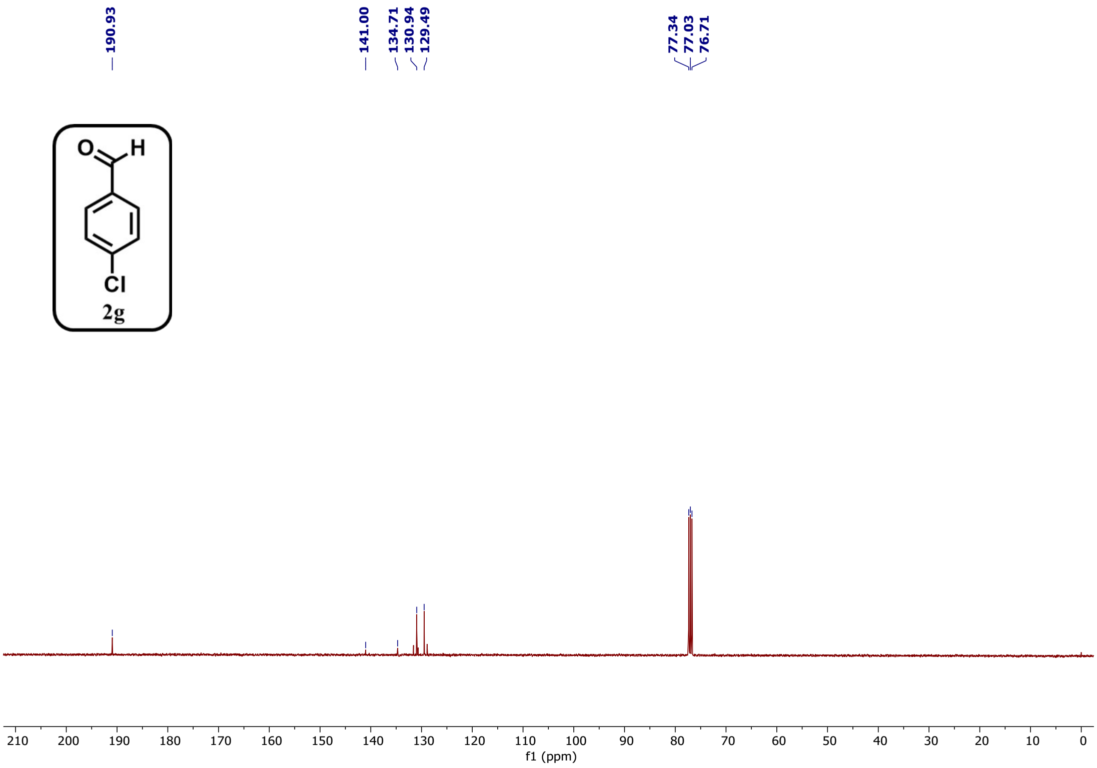

^{1}H NMR spectra of product 2h

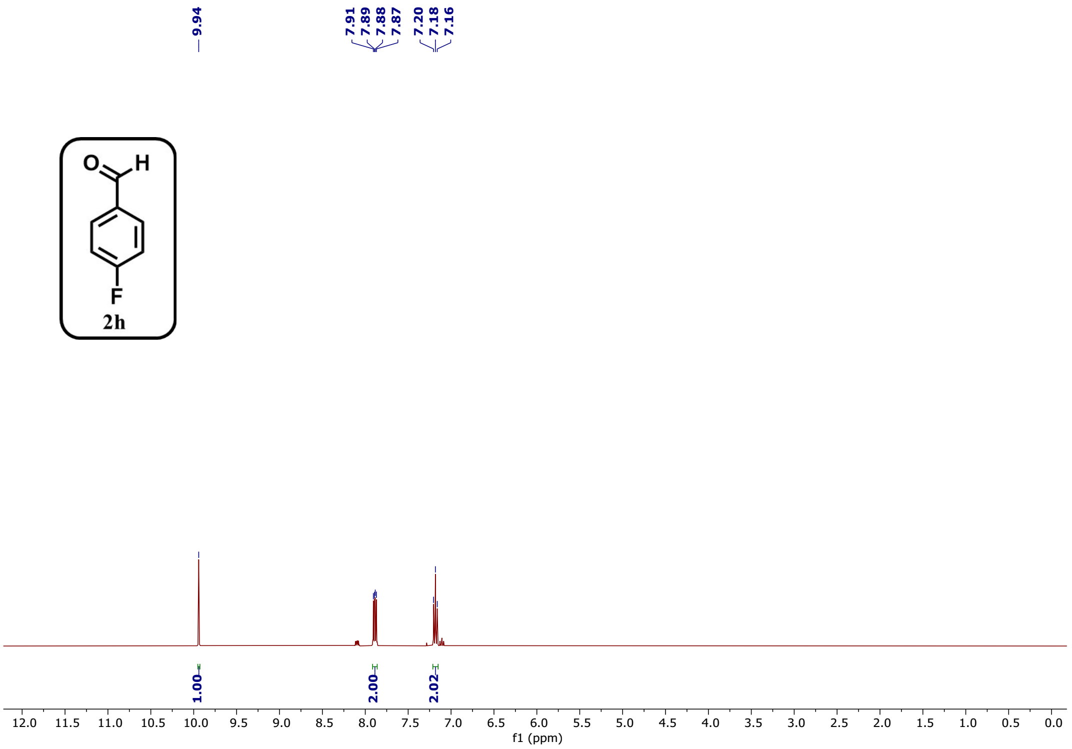

^{13}C NMR spectra of product 2h

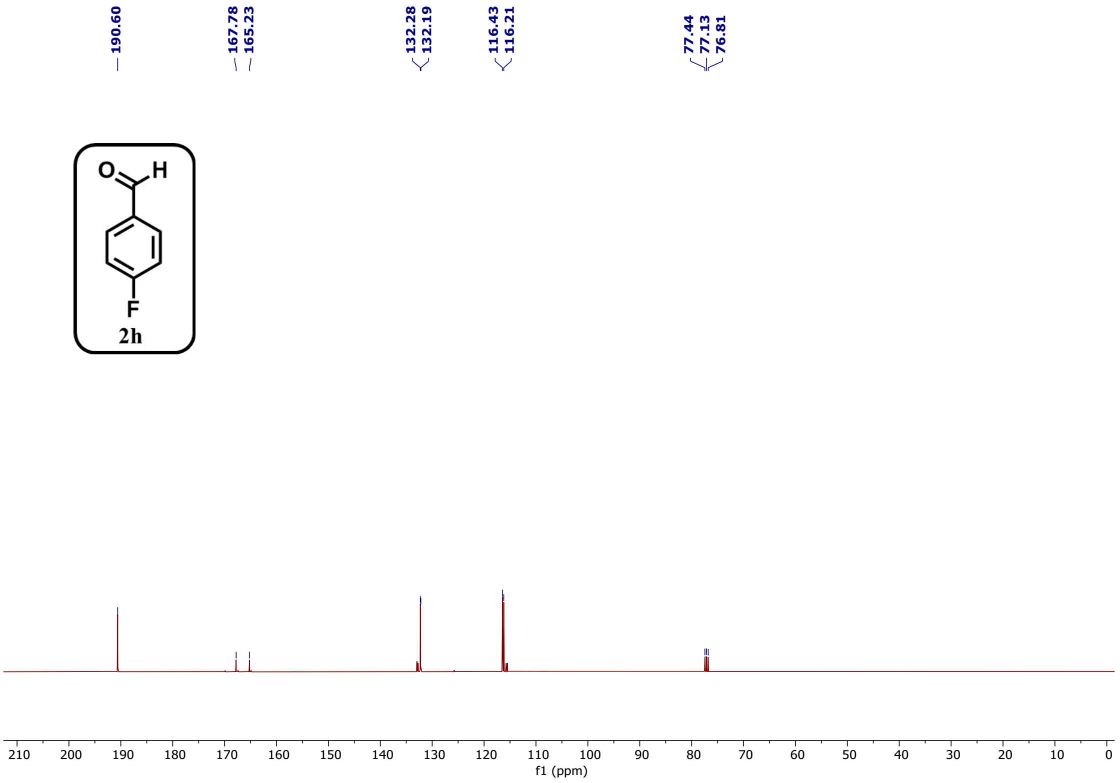

^{1}H NMR spectra of product 2i

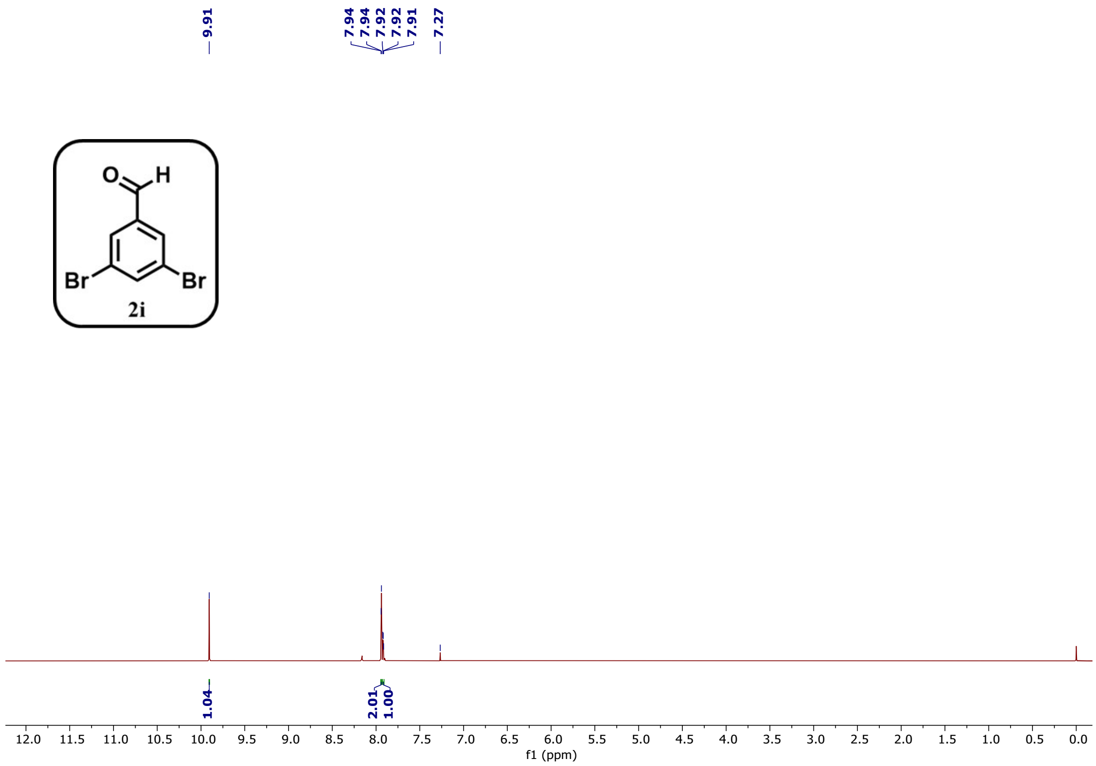

^{13}C NMR spectra of product 2i

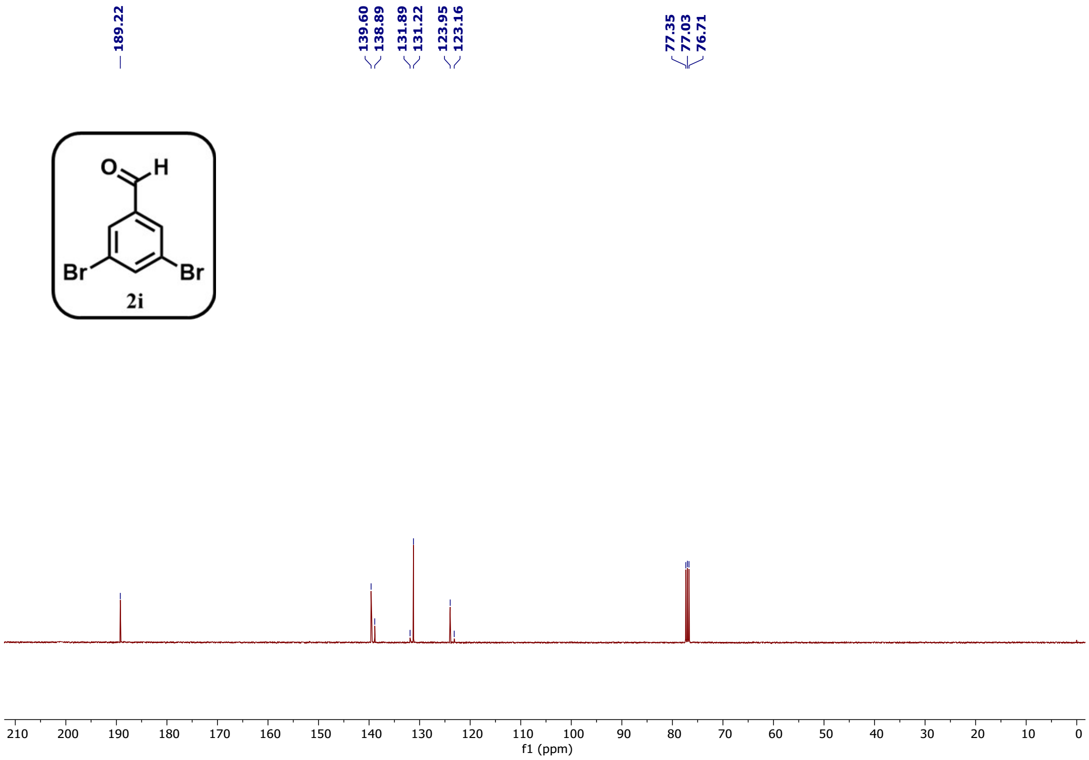

^{1}H NMR spectra of product 2j

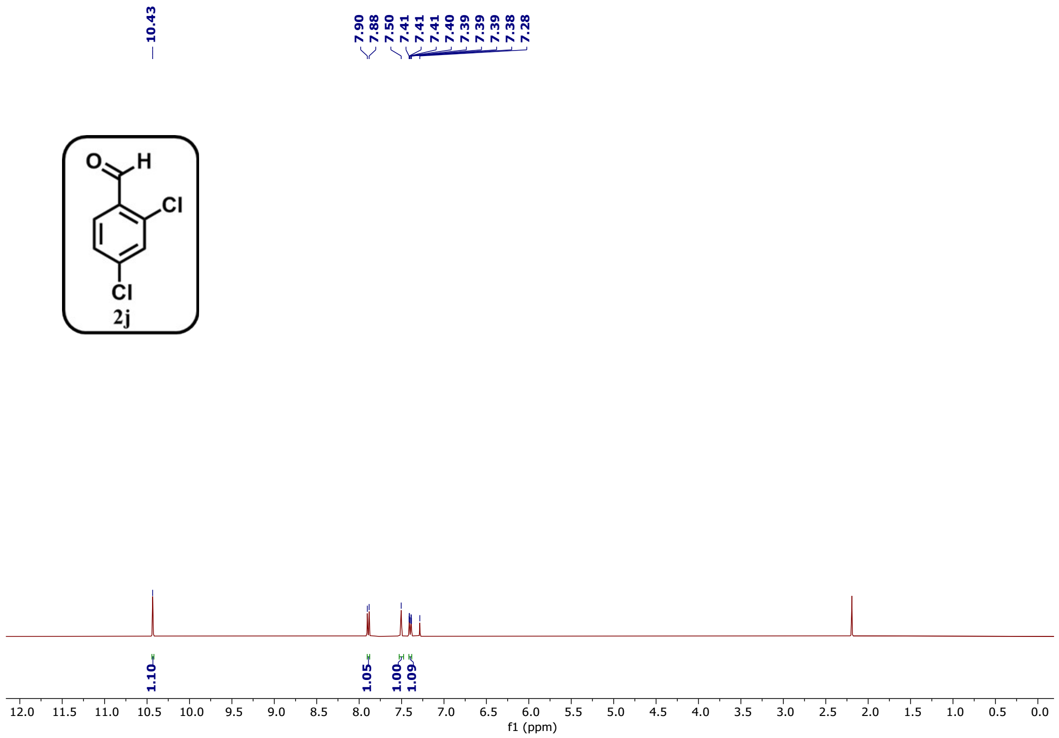

^{13}C NMR spectra of product 2j

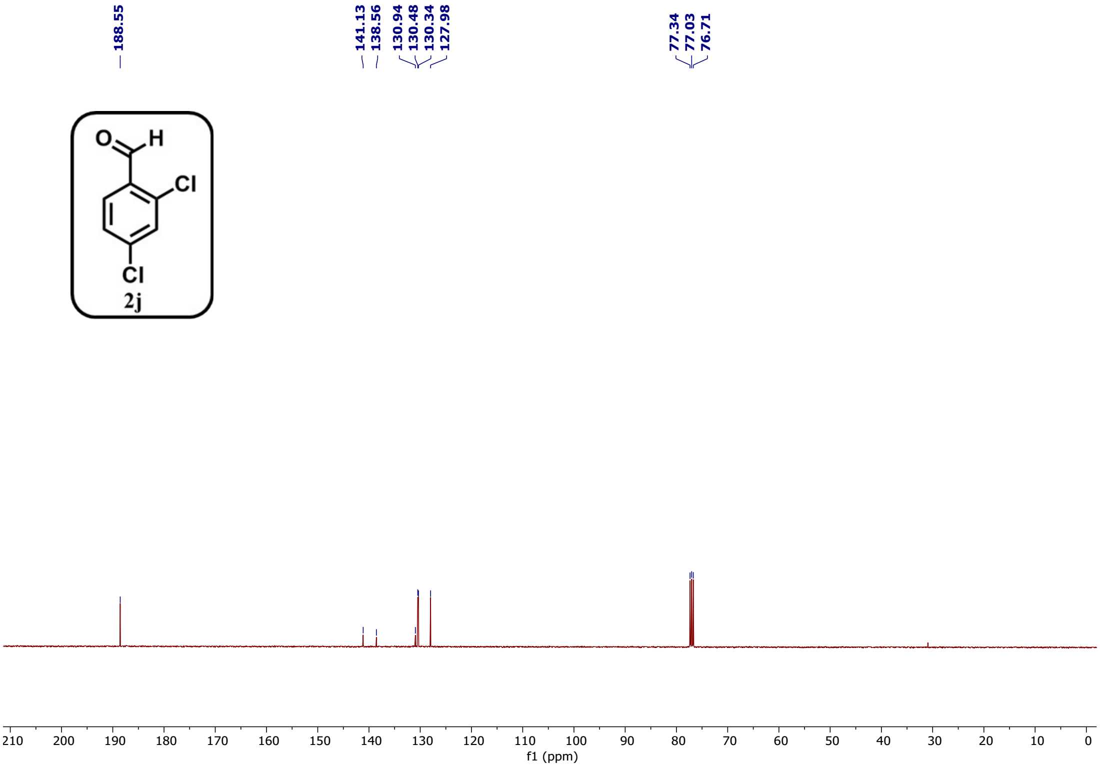

^{1}H NMR spectra of product 2k

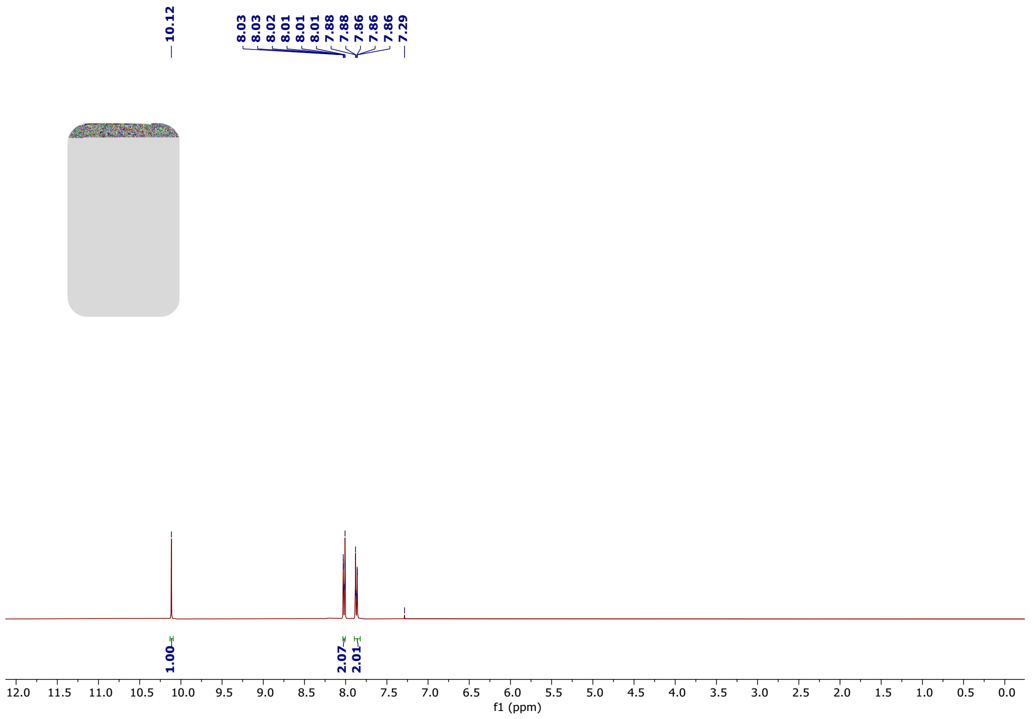

^{13}C NMR spectra of product 2k

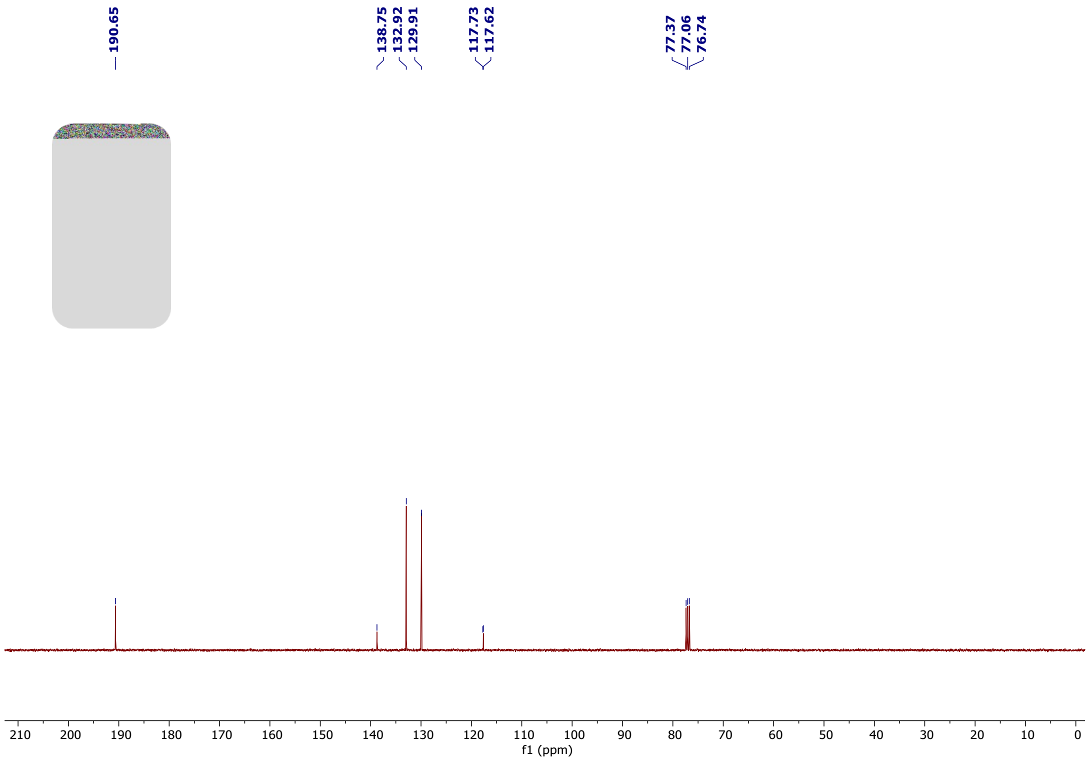

^{1}H NMR spectra of product 2l

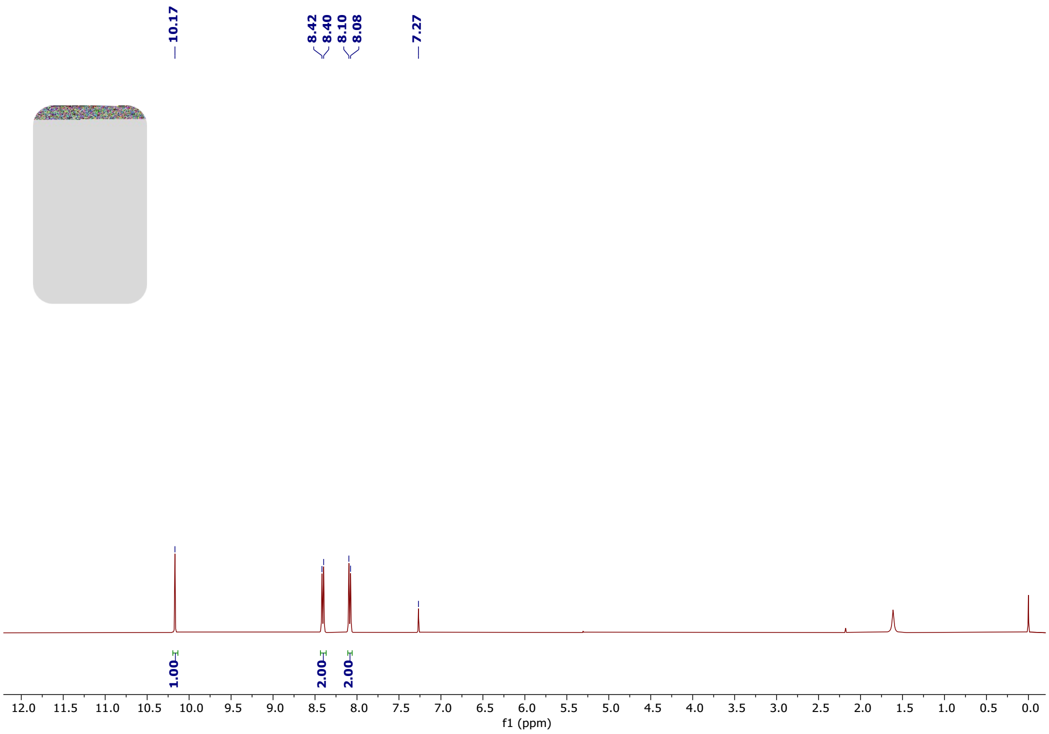

^{13}C NMR spectra of product 2l

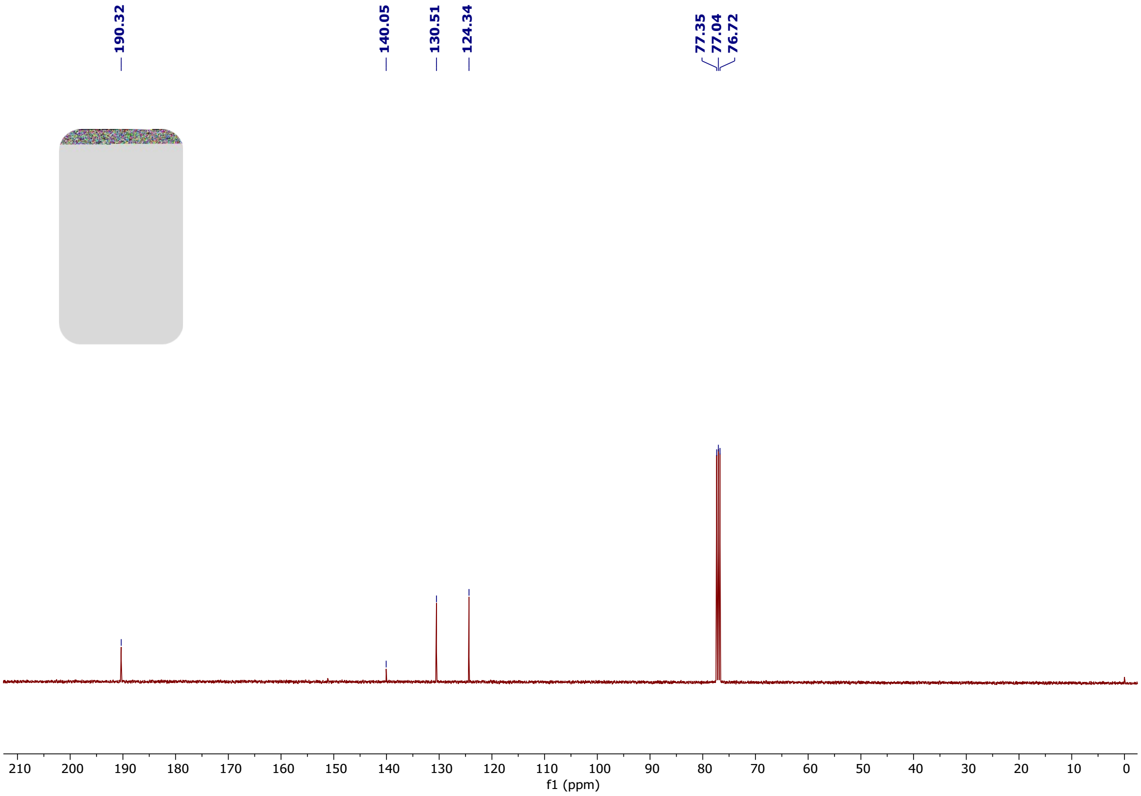

^{1}H NMR spectra of product 2m

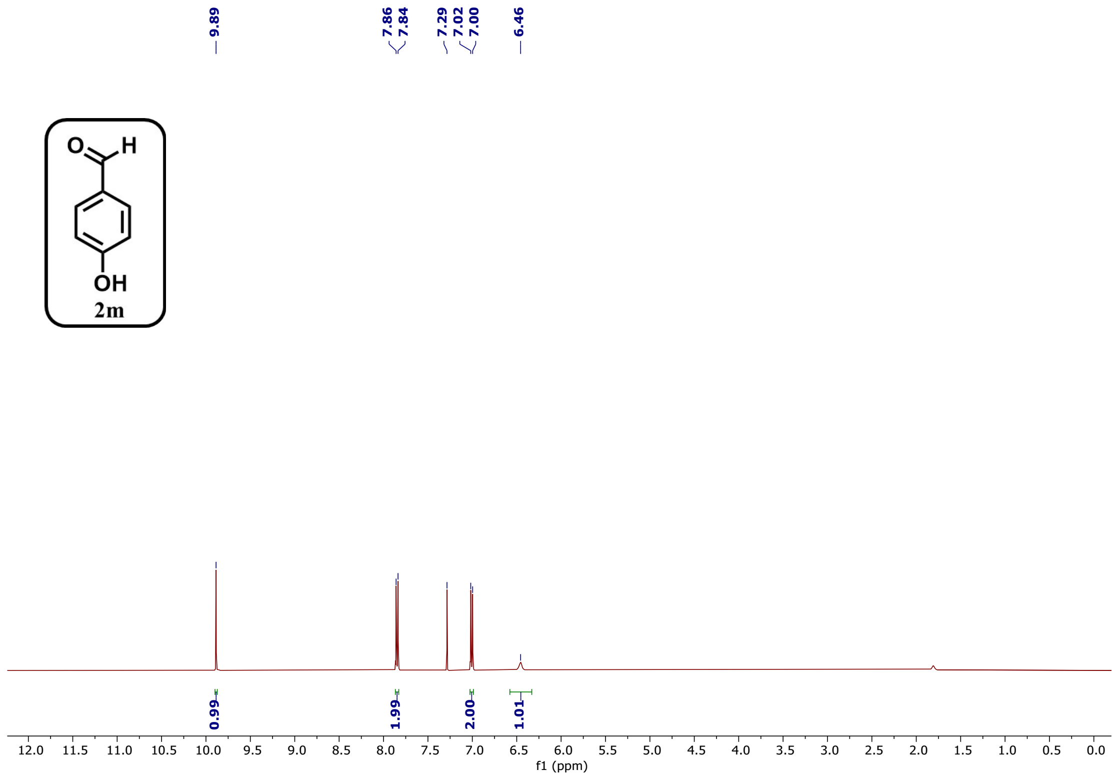

^{13}C NMR spectra of product 2m

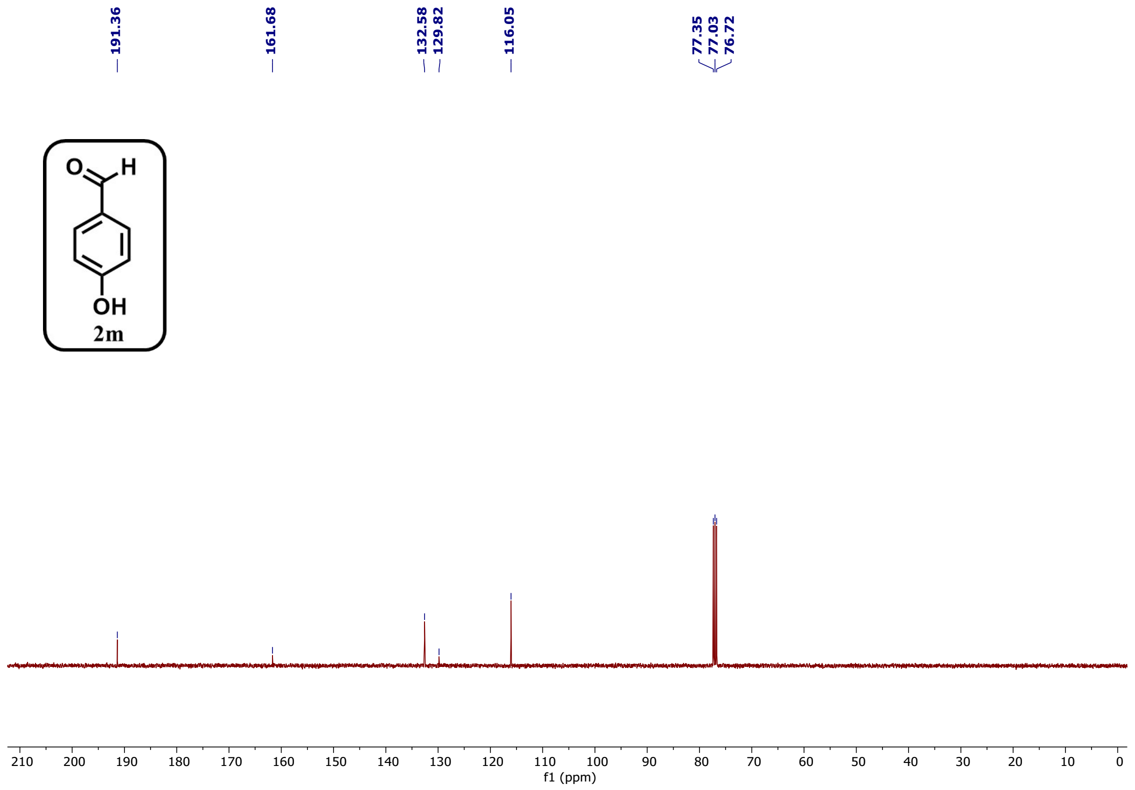

^{1}H NMR spectra of product 2n

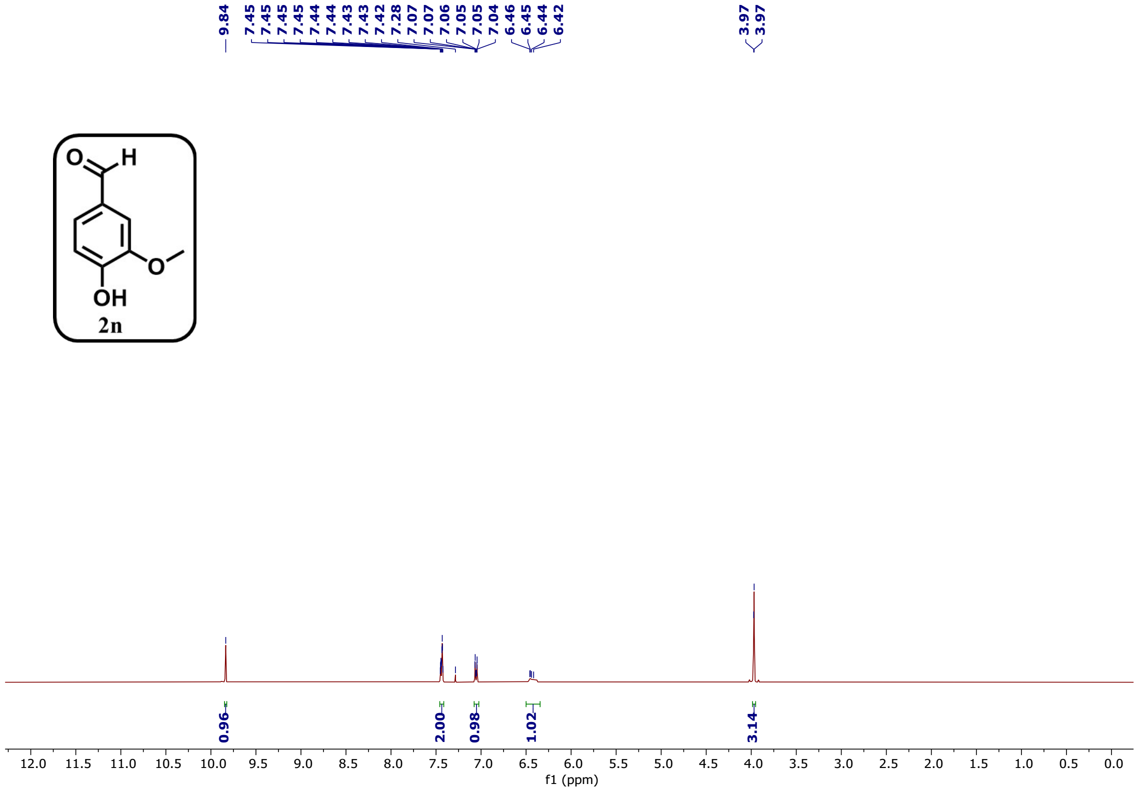

^{13}C NMR spectra of product 2n

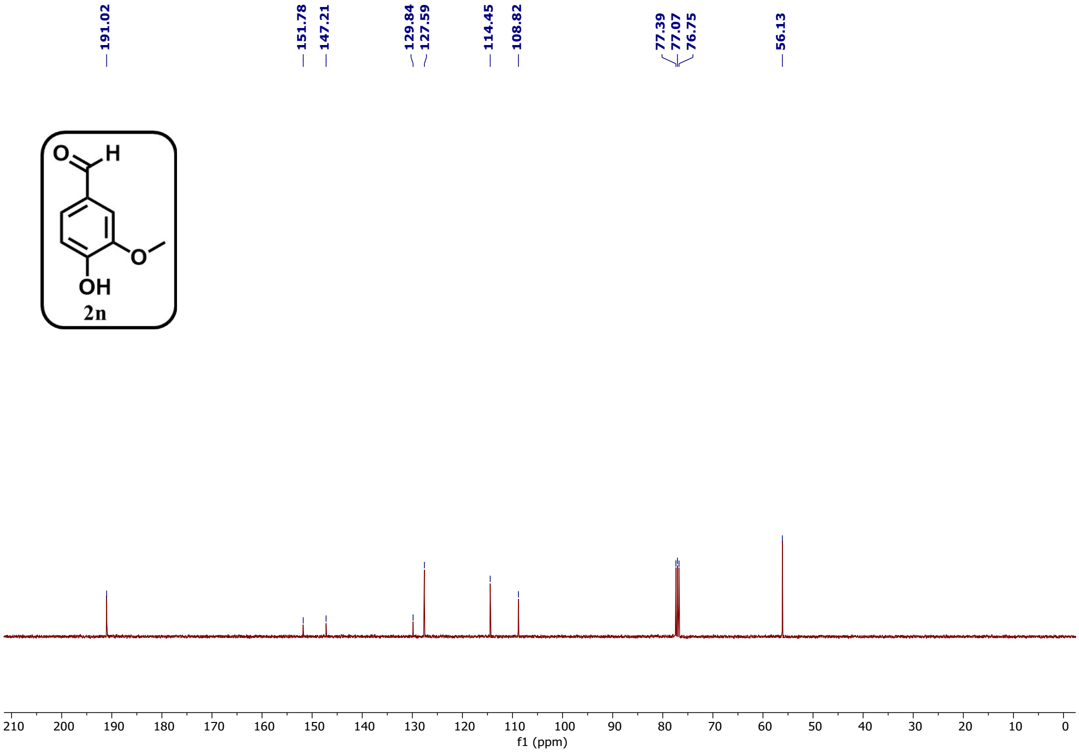

^{1}H NMR spectra of product 2o

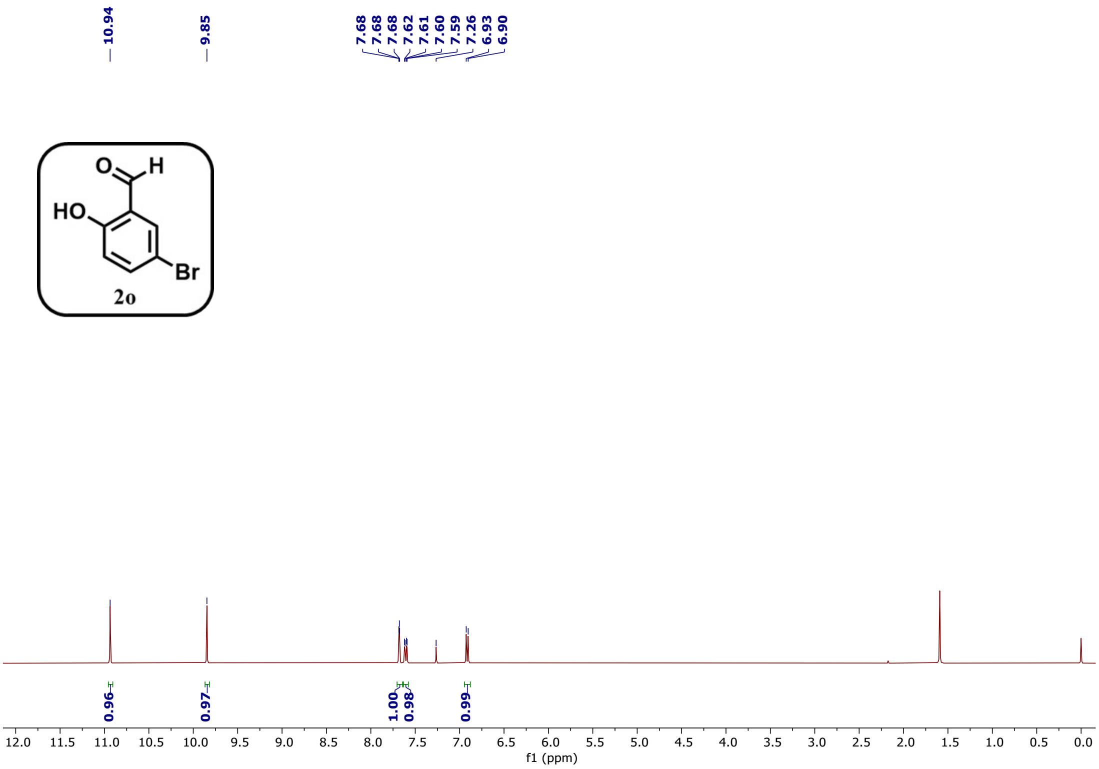

^{13}C NMR spectra of product 2o

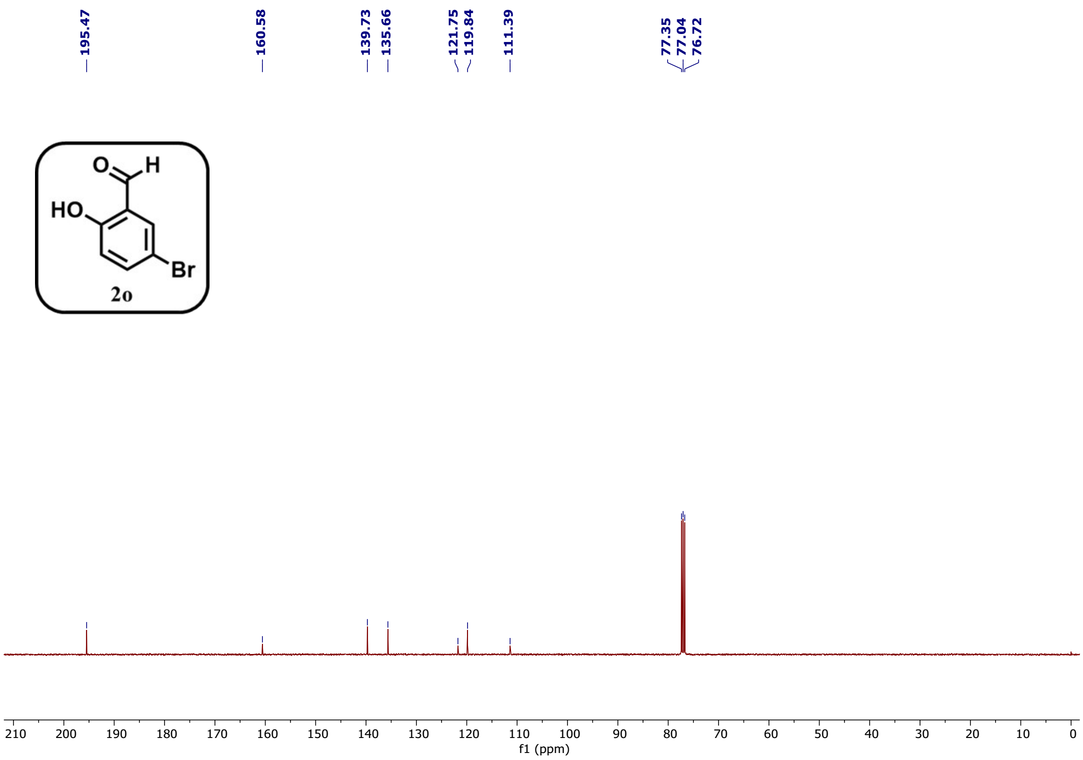

^{1}H NMR spectra of product 2p

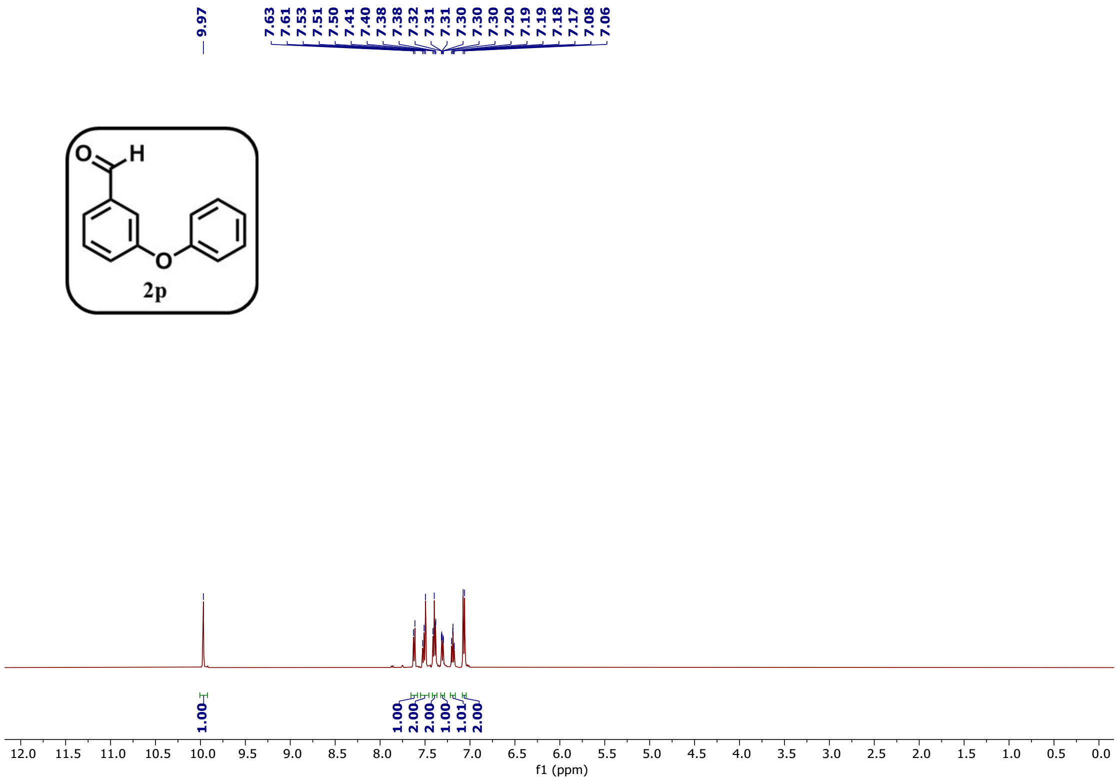

^{13}C NMR spectra of product 2p

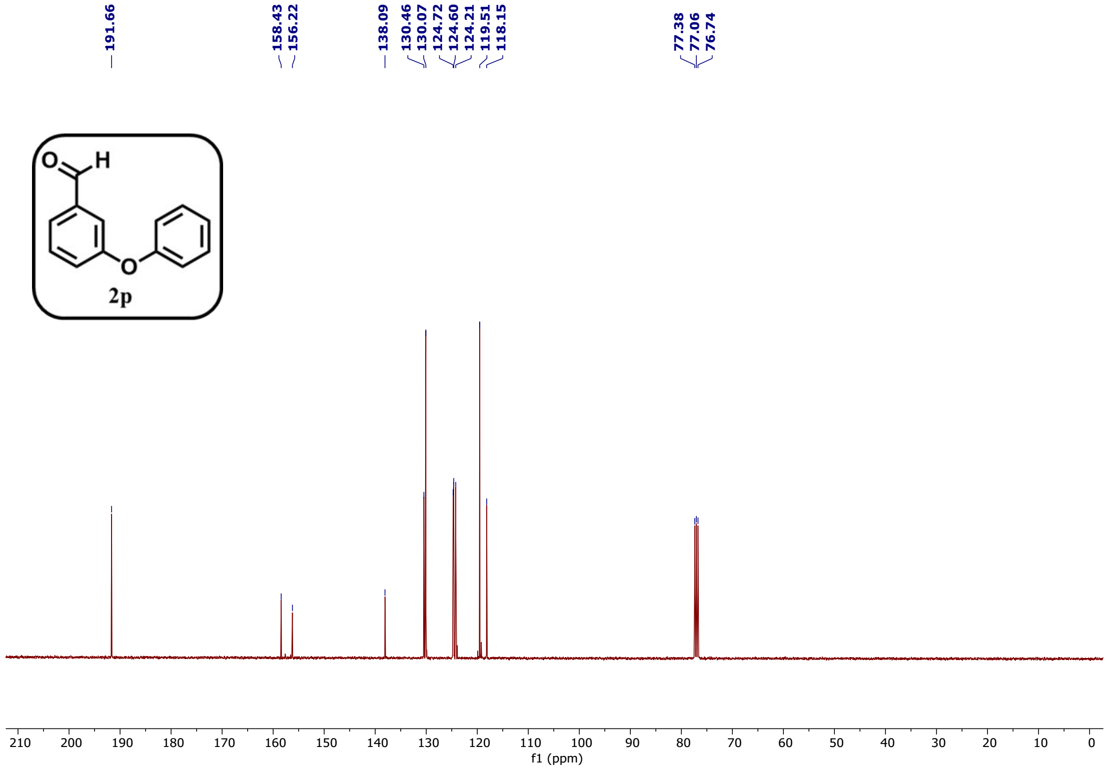

^{1}H NMR spectra of product 2q

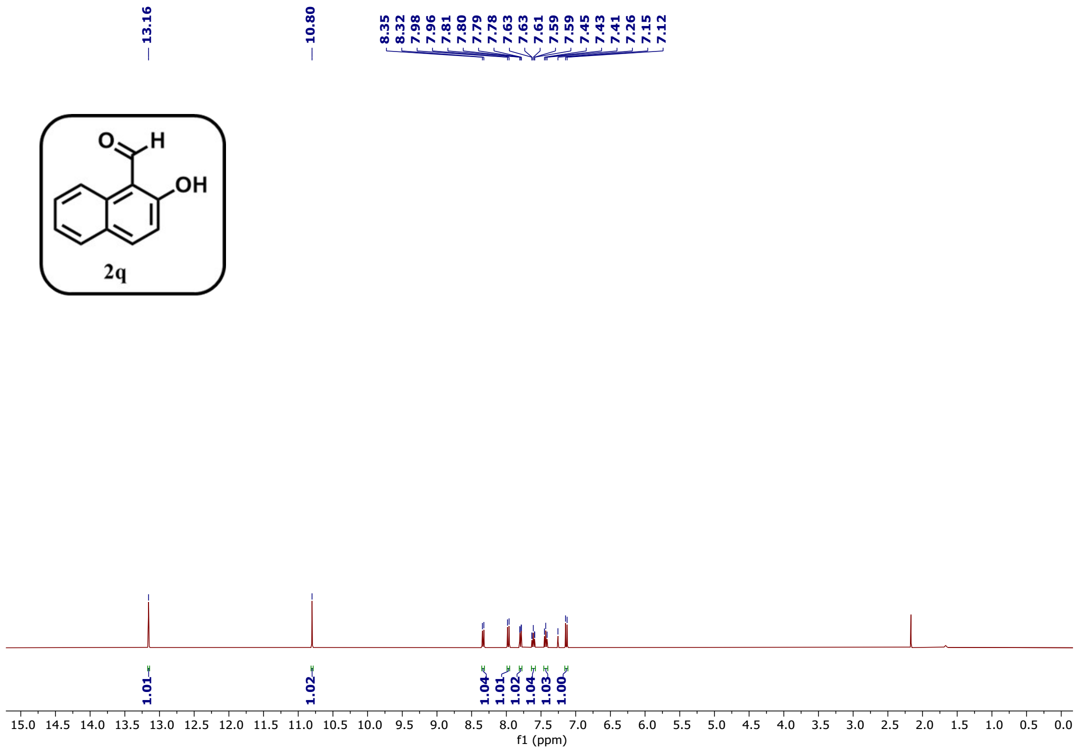

^{13}C NMR spectra of product 2q

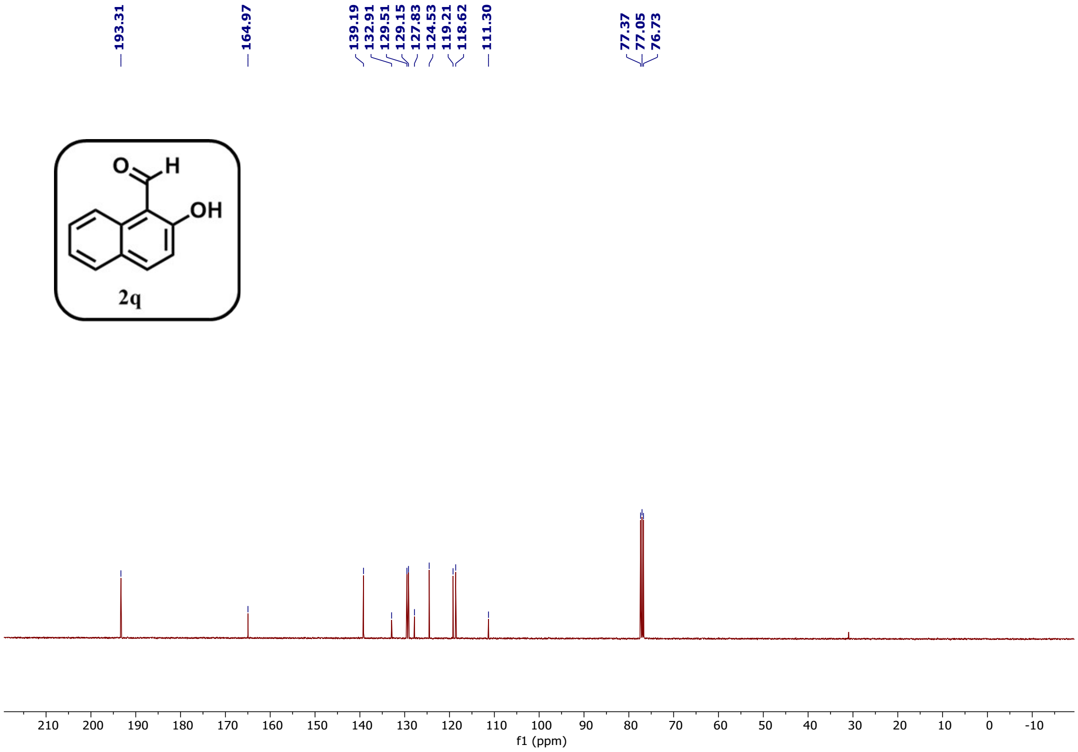

^{1}H NMR spectra of product 2r

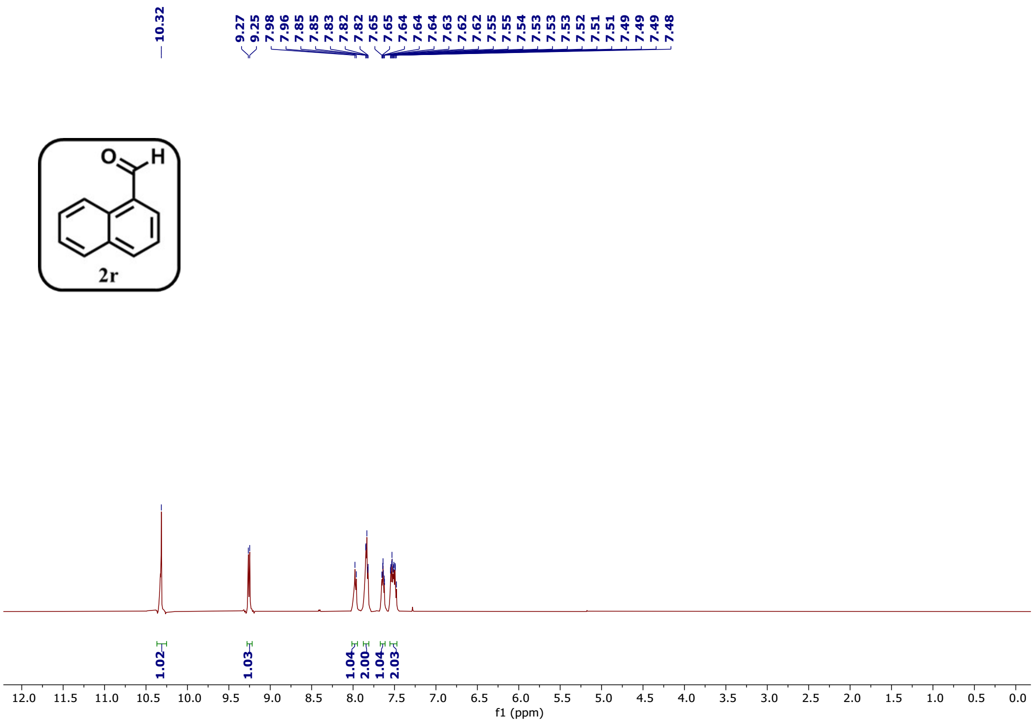

^{13}C NMR spectra of product 2r

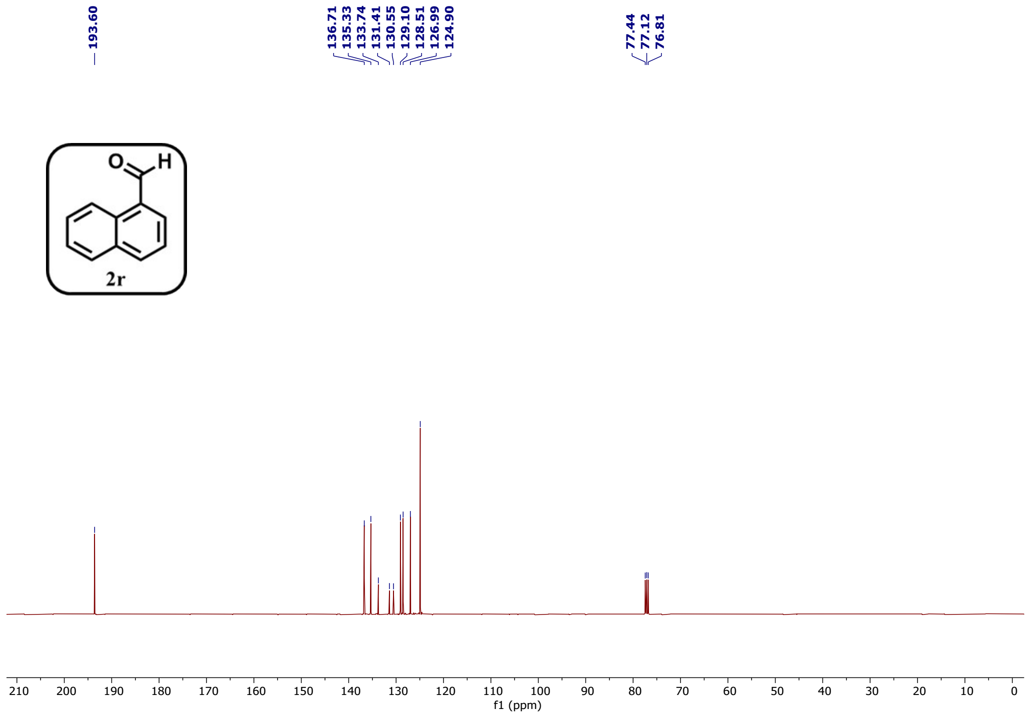

^{1}H NMR spectra of product 2s

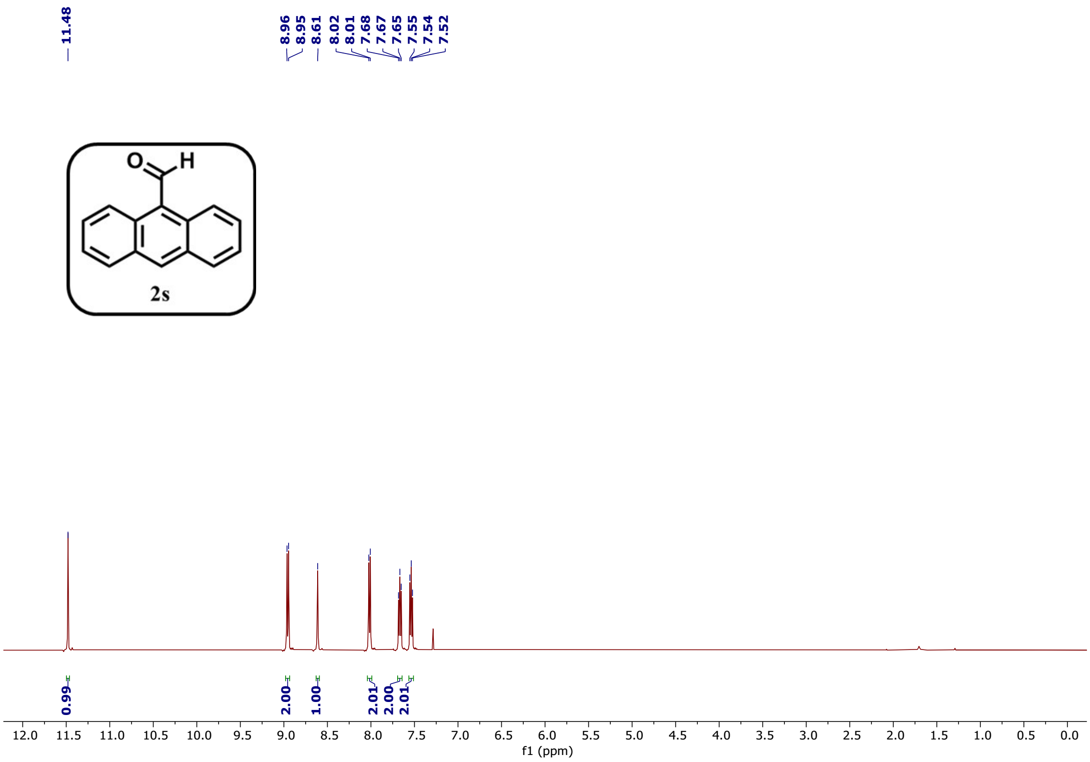

^{13}C NMR spectra of product 2s

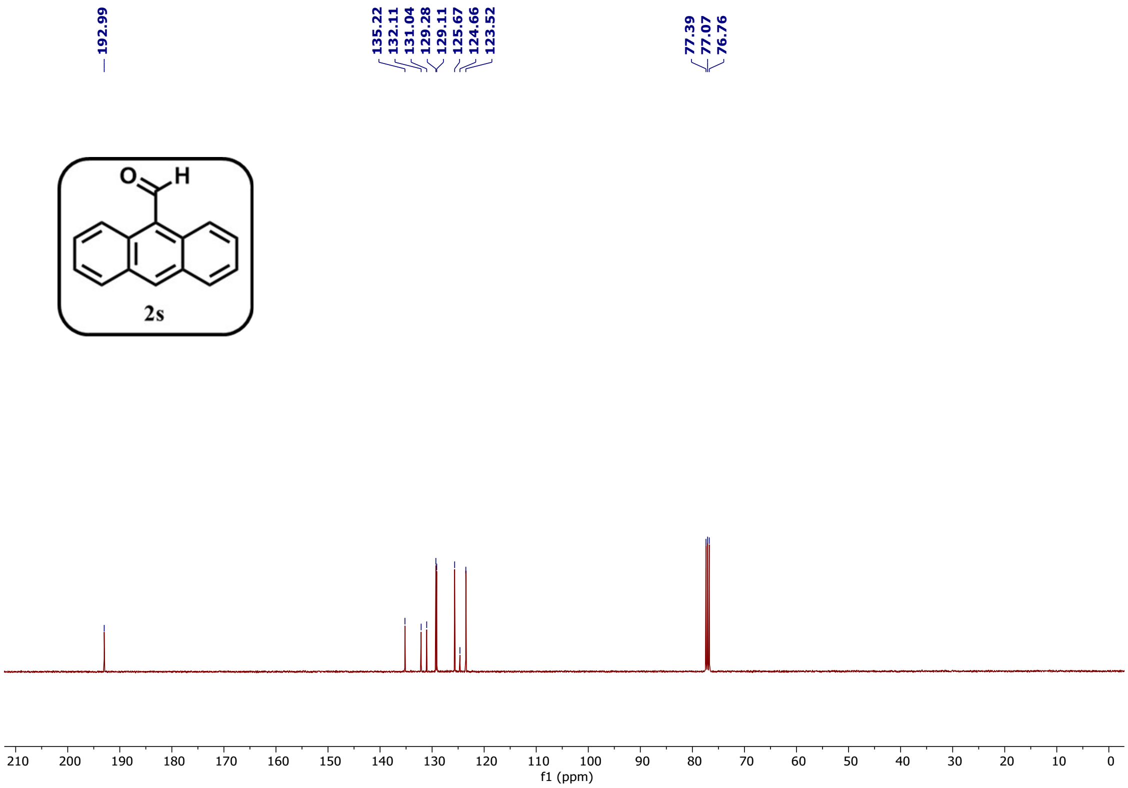

^{1}H NMR spectra of product 2t

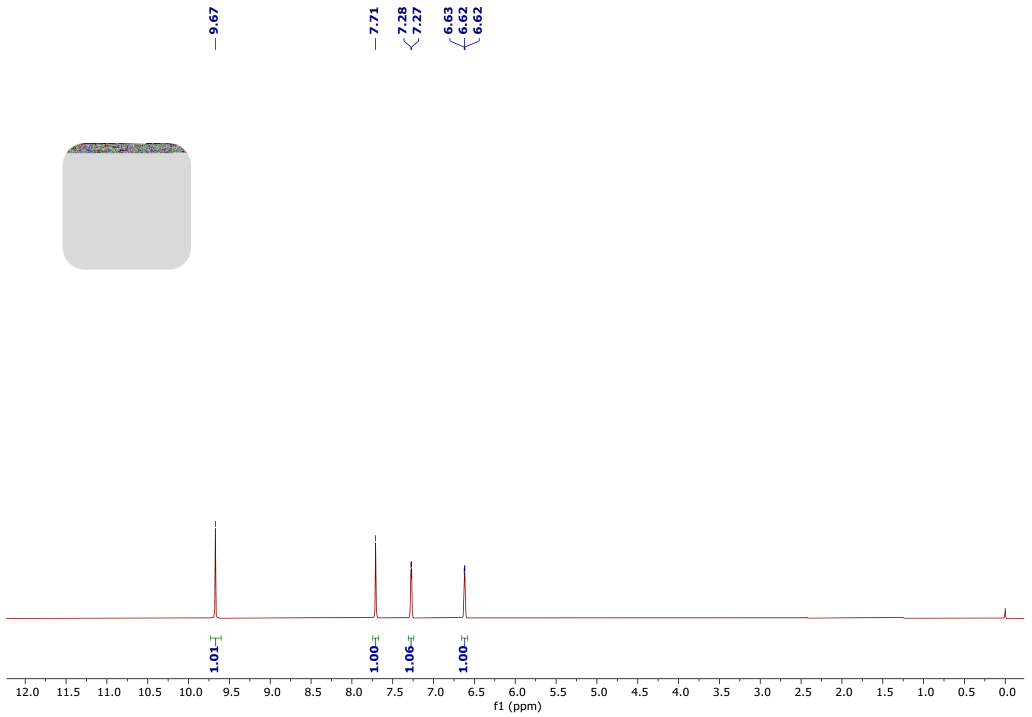

^{13}C NMR spectra of product 2t

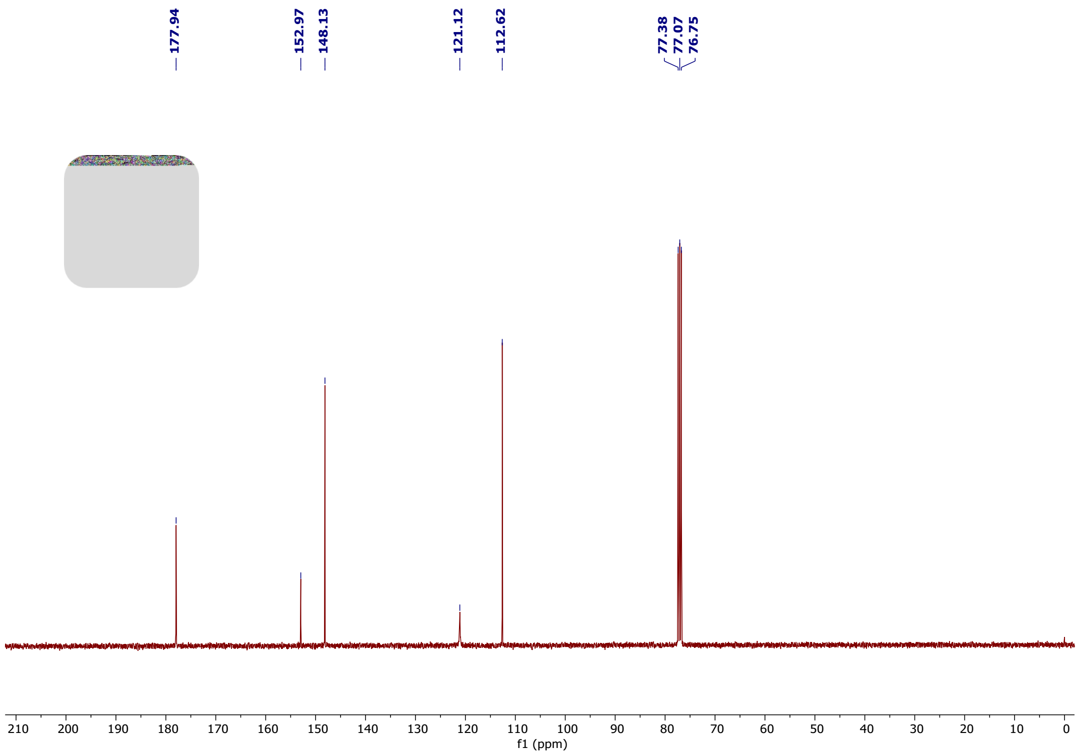

^{1}H NMR spectra of product 2u

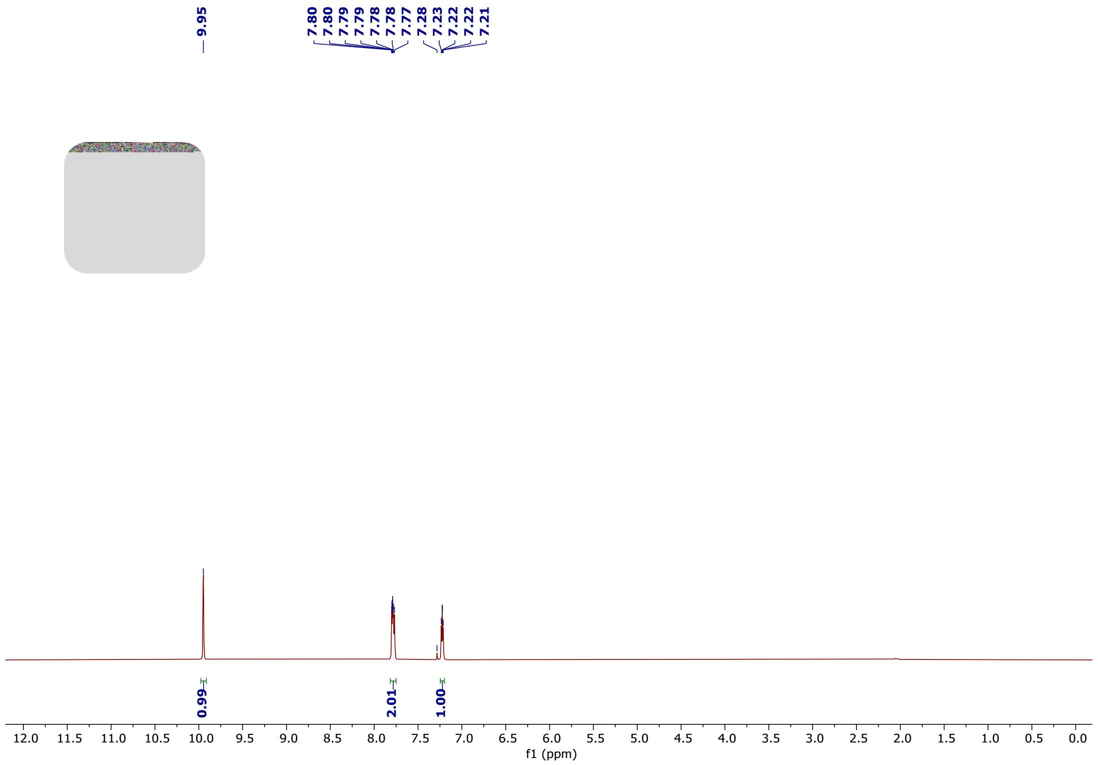

^{13}C NMR spectra of product 2u

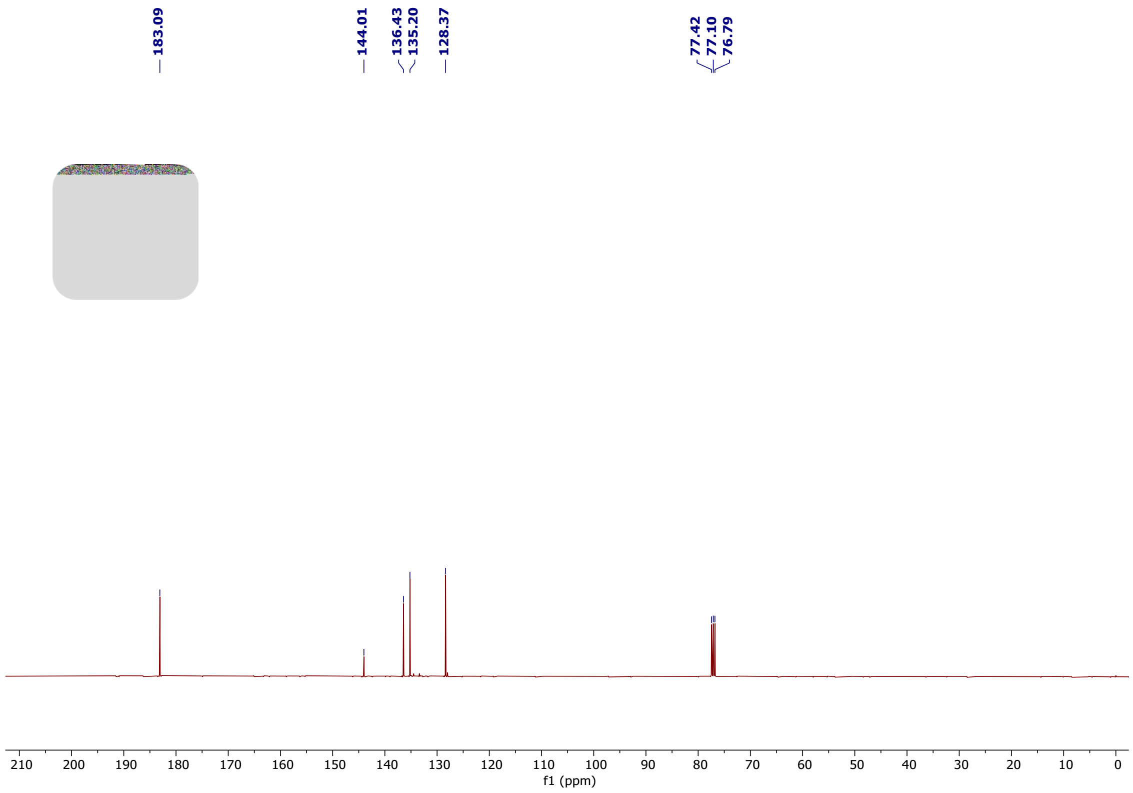

^{1}H NMR spectra of product 2v

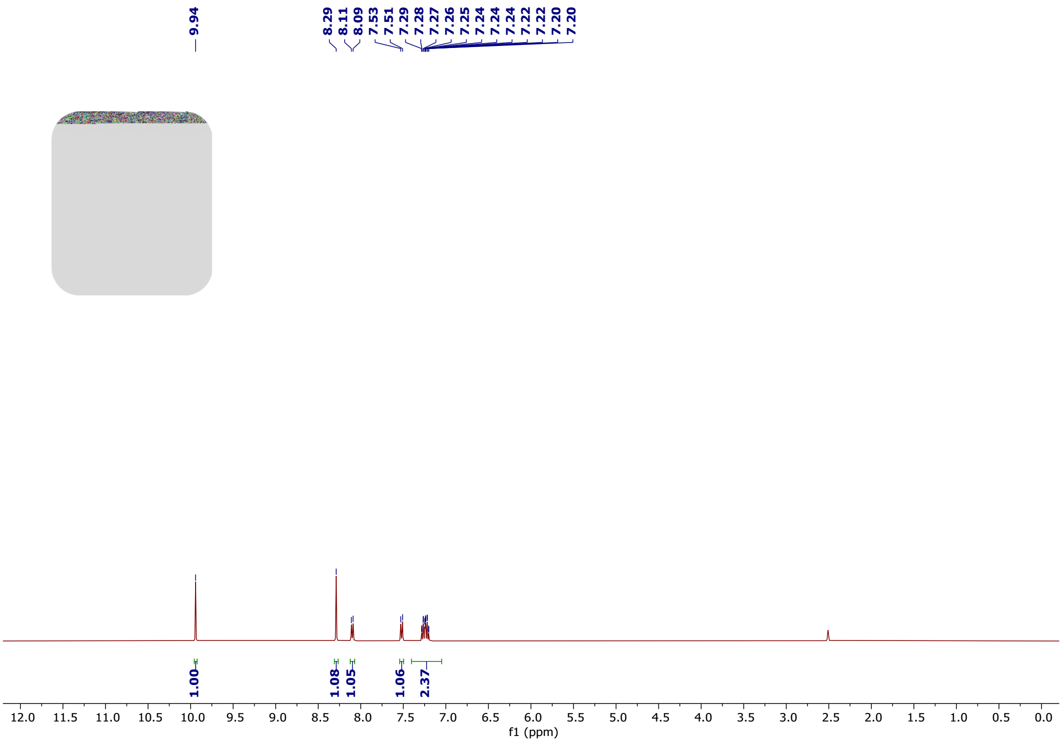

^{13}C NMR spectra of product 2v

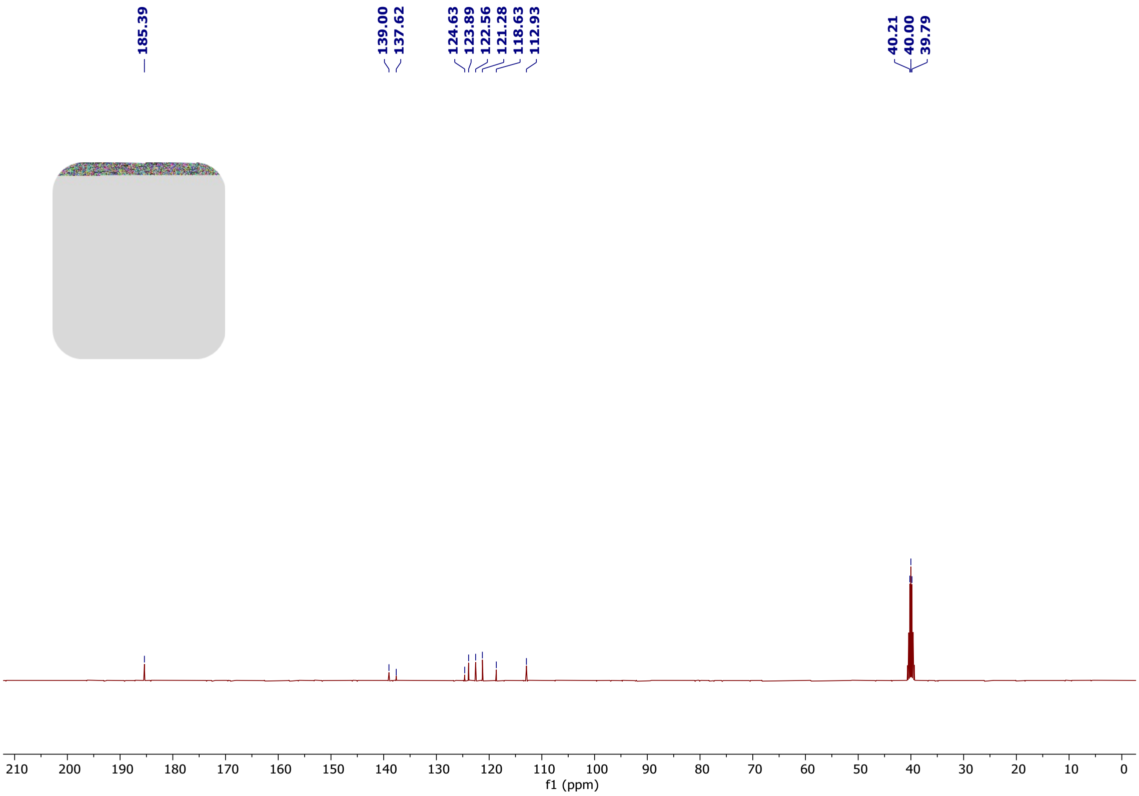

^{1}H NMR spectra of 4-methoxy benzoic acid

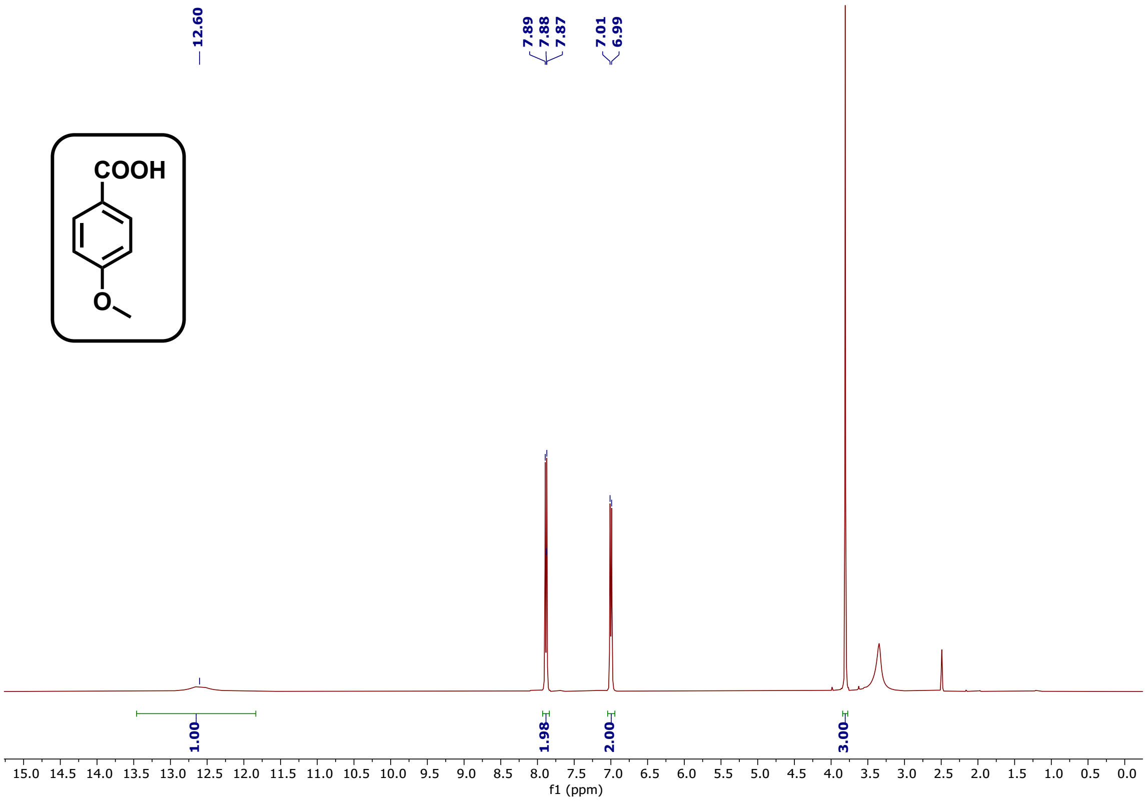

^{13}C NMR spectra of 4-methoxy benzoic acid

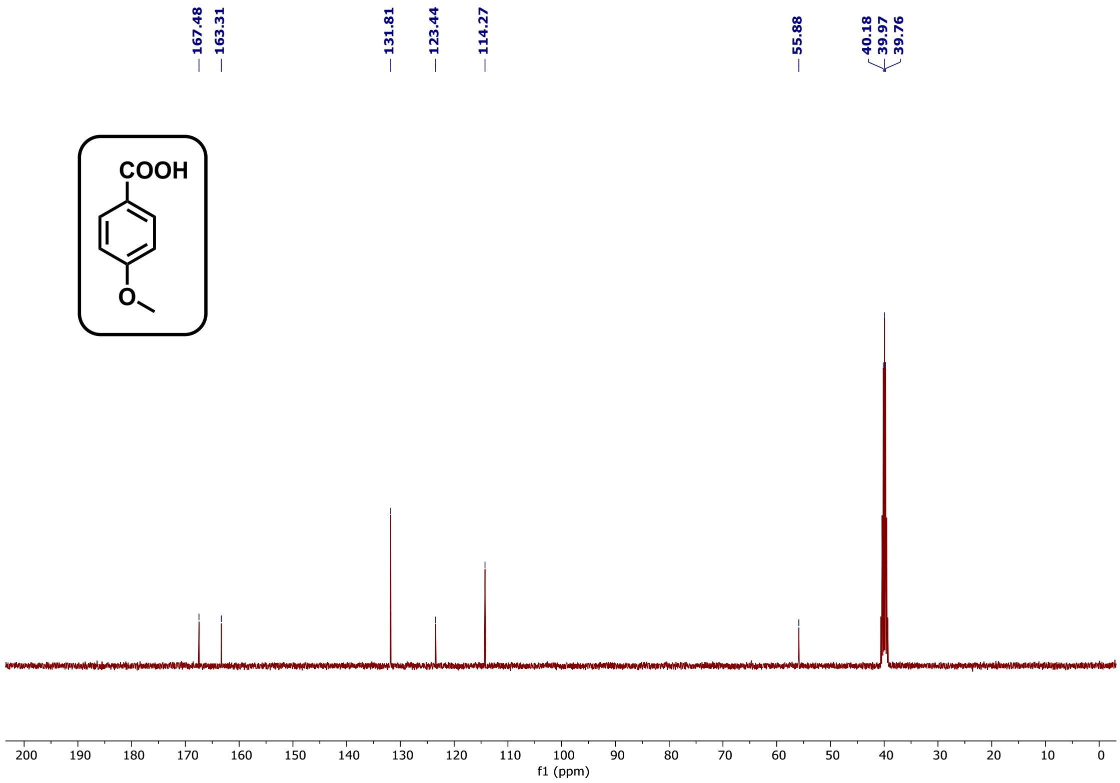

3. Zero Pass CHEM21 green metrics toolkit

Supplementary Information: Appendix 2 **Summary of Zero Pass Metrics Toolkit**

Yield, conversion, selectivity, AE, RME

Reactant (Limiting Reactant First)	Mass (g)	MW	Mol	Catalyst	Mass (g)	Reagent	Mass (g)	Reaction solvent	Volume (cm^3)	Density (g ml^{-1})	Mass (g)	Work up chemical	Mass (g)	Workup solvent	Volume (cm3)	Density (g ml^{-1})	Mass (g)
4-methoxy benzyl alcoho	0.050	138.16	0.00	Ni	0.02			Isopropanol	2.00	0.79	1.57						0.00
Oxygen		32.00	0.00								0.00						0.00
Total	0.05	170.16			0.02		0.00				1.57		0.00				0.00

$$AE = \frac{molecular\ weight\ of\ product}{total\ molecualr\ weight\ of\ reactants} \times 100$$

$$RME = \frac{mass\ of\ isolated\ product}{total\ mass\ of\ reactants} \times 100$$

Metric	Value	Flag	
Yield	93.0	●	93.0
Conversion	94.0	●	94.0
Selectivity	98.9	●	98.9
AE	80.0		
RME	91.6		

	mass	mw	mol
Product	0.046	136.150	0.0003364

	mass
Unreacted limiting reactant	0.003

Solvents (Zero Pass)

Highly hazardous solvents (Red flag for any of the following)	List Highly Hazardous Solvents Below
Et_2O, Benzene, CCl_4, chloroform, DCE, nitromethane, CS_2, HMPA	None

Health and Safety (Zero Pass)

Health & safety (Red flag for any of the following)		List substances plus the red flagged H-codes below
Highly explosive	H200, H201, H202, H203	None
Explosive thermal runaway	H240	None
Fatally toxic	H300, H310, H330	None
Mutagenic	H350	None
Repro-toxic	H360	None
Serious environmental implications	H420	None

4. First Pass CHEM21 green metrics toolkit

Supplementary Information: Appendix 2 — Summary of First Pass Metrics Toolkit

Yield, AE, RME, MI/PMI and OE

Reactant (Limiting Reactant First)	Mass (g)	MW	Mol	Catalyst	Mass (g)	Reagent	Mass (g)	Reaction solvent	Volume (cm^3)	Density ($g\ ml^{-1}$)	Mass (g)	Work up chemical	Mass (g)	Workup solvent	Volume (cm3)	Density ($g\ ml^{-1}$)	Mass (g)
4-methoxy benzyl alcohol	0.050	138.16	0.000	Ni	0.02			Isopropanol	2.00	0.79	1.57						0.00
Oxygen		32.00	0.00								0.00						0.00
Total	0.05	170.16			0.02		0.00				1.57		0.00				0.00

$$RME = \frac{mass\ of\ isolated\ product}{total\ mass\ of\ reactants} \times 100$$

$$AE = \frac{molecular\ weight\ of\ product}{total\ molecualr\ weight\ of\ reactants} \times 100$$

$$mass\ intensity = \frac{total\ mass\ in\ a\ process\ or\ process\ step}{mass\ of\ product}$$

$$OE = \frac{RME}{AE} \times 100$$

Metric	Value	Flag	
Yield	93.0		93.0
Conversion	94.0		94.0
Selectivity	98.9		98.9
AE	80.0		
RME	91.6	OE	114.5
PMI total	35.7		
PMI Reaction	35.7		
PMI reactants, reagents, catlyst	1.4		
PMI reaction solvents	34.3		
PMI Workup	0.0		
PMI Workup chemical	0.0		
PMI workup solvents	0.0		

	Mass	MW	Mol
Product	0.046	136.15	0.0003
	mass		
Unreacted limiting reactant	0.003		

Solvents (First Pass)

		List solvents below
**Preferred solvents**	**water,** EtOH, nBuOH, AcOipr, AcOnBu, PhOMe, MeOH, tBuOH, BnOH, ethylene glycol, acetone, MEK, MIBK, **AcOEt,** sulfolane	i-PrOH
**Problematic solvents:** (acceptable only if substitution does not offer advantages)	DMSO, cyclohexanone, DMPU, AcOH, Ac2O, Acetonitrile, AcOMe, THF, heptane, Me-cyclohexane, toluene, xylene, MTBE, **cyclohexane**, chlorobenzene, formic acid, pyridine, Me-THF	
**Hazardous solvents**: These solvents have significant health and/or safety concerns.	dioxane, pentane, TEA, diisopropyl ether, DME, DCM, DMF, DMA, NMP, methoxyethanol, hexane	
**Highly hazardous solvents:** The solvents which are agreed not to be used, even in screening	Et_2O, Benzene, CCl_4, chloroform, DCE, nitromethane, CS_2, HMPA	

Catalyst/enzyme (First Pass)

		Tick
Catalyst or enzyme used, **or** reaction takes place without **any** catalyst/reagents.	Green Flag	*
Use of stoichiometric quantities of reagents	Amber Flag	
Use of reagents in excess	Red Flag	

		Tick
Facile recovery of catalyst/enzyme	Green Flag	*
catalyst/enzyme not recovered	Amber Flag	

Critical elements

Supply remaining	Flag colour	Note element
5-50 years	Red Flag	
50-500 years	Amber Flag	Ni
+500 years	Green Flag	

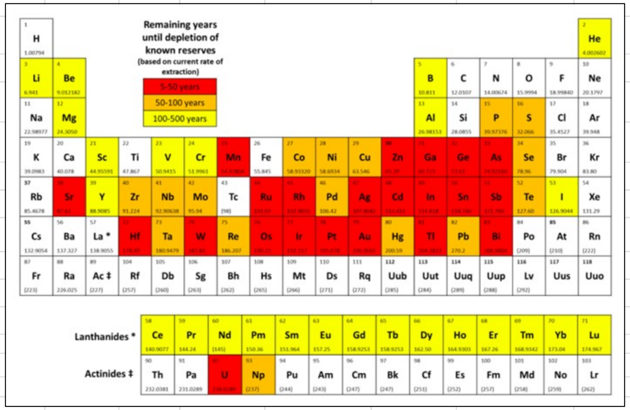

Energy (First Pass)

		Tick
Reaction run between 0 to 70°C	Green Flag	*
Reaction run between -20 to 0 or 70 to 140°C	Amber Flag	
Reaction run below -20 or above 140°C	Red Flag	

		Tick
Reaction run at reflux	Red Flag	
Reaction run 5°C or more below the solvent boiling point	Green Flag	*

Batch/flow

		Tick
Flow	Green Flag	
Batch	Amber Flag	*

Work Up

		List
quenching<br>filtration<br>centrifugation<br>crystallisation<br>Low temperature distillation/evaporation/ sublimation (< 140 °C at atmospheric	Green Flag	
solvent exchange, quenching into aqueous solvent	Amber Flag	
chromatography/ion exchange<br>high temperature<br>multiple recrystallisation	Red Flag	Column Chromatography

Health & safety

	Red Flag	Amber Flag	Green Flag	List substances and H-codes	List substances and H-codes	List substances and H-codes
**Highly explosive**	H200, H201, H202, H203	H205, H220, H224	If no red or amber flagged H codes present then green flag	None	None	**Methoxy benzyl alcohol:** H315, H-317, H319
**Explosive thermal runaway**	H230, H240, H250	H241				
**Toxic**	H300, H310, H330	H301, H311, H331,				
**Long Term toxicity**	H340, H350, H360, H370, H372	H341, H351, H361, H371, H373				
**Environmental implications**	H400, H410, H411, H420	H401, H412				

Use of chemicals of environmental concern

		List substances of very high concern
Chemical identified as Substances of Very High Concern by ChemSec which are utilised	Red Flag	None

5. Reference

1. Liang, D.; Yan, J.; Yin, X.; Wang, Y.; Du, J.; Qian, J.; He, M.; Zhou, W. J. C. S.; Technology, Effects of doping metal on the catalytic performance of manganese-based layered double hydroxides in the aerobic oxidation of alcohols. *Catal. Sci. Technol.* **2025,** *15* (19), 5876-5885.
2. Love, B. E. J. O. P.; International, P., Facile Preparation of 3, 6-Dimethoxy-2-nitrobenzaldehyde. *Org. Prep. Proced. Int.* **2024,** *56* (3), 302-306.
3. Liu, X.; Xia, Q.; Zhang, Y.; Chen, C.; Chen, W. J. T. J. o. O. C., Cu-NHC-TEMPO catalyzed aerobic oxidation of primary alcohols to aldehydes. *J. Org. Chem.* **2013,** *78* (17), 8531-8536.
4. Dutta, M.; Bora, J.; Kalita, A. j.; Suleman, A.; Chutia, U.; Chetia, B. J. A. A. N. M., Ag-Decorated NiFe2O4 Magnetic Nanocomposites as Catalysts for the Synthesis of 5-Substituted 1 H-Tetrazoles and for the Selective Oxidation of Benzyl Alcohol to Benzaldehyde. *ACS Appl. Nano Mater.* **2025,** *8* (47), 22670-22682.
5. Kitt, M. I.; Amir, E.; Sloane, E. R.; Fraser, D. G.; Cerritelli, J. E.; Sabanos, C. S.; McNeely, J. H.; Snyder, J. K.; Doerrer, L. H.; Beeler, A. B. J. A. C., Chemoselective aerobic oxidation of alcohols utilizing a vanadium (V) catalyst. *ACS Catal.* **2024,** *14* (7), 4799-4806.
6. Geng, L.; Zheng, B.; Wang, X.; Zhang, W.; Wu, S.; Jia, M.; Yan, W.; Liu, G. J. C., Fe3O4 nanoparticles anchored on carbon serve the dual role of catalyst and magnetically recoverable entity in the aerobic oxidation of alcohols. *ChemCatChem* **2016,** *8* (4), 805-811.
